# Minimal vs Specialized Exercise Equipment for Pulmonary Rehabilitation: A Randomized Clinical Trial

**DOI:** 10.1001/jamanetworkopen.2025.26616

**Published:** 2025-08-12

**Authors:** Claire M. Nolan, Callum Glen, Jessica A. Walsh, Suhani Patel, Ruth E. Barker, Oliver Polgar, Nannette Spain, Hannah Littlemore, Peter Jung, George D. Edwards, Timothy O. Jenkins, Jennifer Harvey, Karen Ingram, Christopher Newby, Michael Steiner, Rebecca Wilson, Wei Gao, Francesca Fiorentino, Emeka Chukwusa, Peter May, Matthew Maddocks, William D. C. Man

**Affiliations:** 1 Department of Health Sciences, College of Health, Medicine, and Life Sciences, Brunel University of London, Uxbridge, Middlesex, UK; 2 Harefield Respiratory Research Group, Royal Brompton and Harefield Hospitals, Guy’s and St Thomas’ National Health Service Foundation Trust, Harefield, Middlesex, UK; 3 Biostatistics and Health Informatics, Institute of Psychiatry, Psychology, and Neuroscience, King’s College London, London, UK; 4 King’s Clinical Trials Unit, King’s College London, London, UK; 5 Sydney School of Health Sciences, Faculty of Medicine and Health, The University of Sydney, Camperdown, New South Wales, Australia; 6 National Heart and Lung Institute, Imperial College London, London, UK; 7 Health Innovation Wessex, Southampton, Hampshire, UK; 8 MISTER Patient and Public Involvement Group, Royal Brompton and Harefield Hospitals, Guy’s and St Thomas’ National Health Service Foundation Trust, Harefield, Middlesex, UK; 9 Harefield Pulmonary Rehabilitation Unit, Royal Brompton and Harefield Hospitals, Guy’s and St Thomas’ National Health Service Foundation Trust, Harefield, Middlesex, UK; 10 Research Support Service, School of Medicine, University of Nottingham, Nottingham, UK; 11 Department of Respiratory Services, Leicester Biomedical Research Centre, National Institute for Health and Care Research, University of Leicester, Leicester, UK; 12 Cicely Saunders Institute of Palliative Care, Policy & Rehabilitation, King’s College London, London, UK; 13 Department of Epidemiology and Health Statistics, School of Public Health, Jiangxi Medical College, Nanchang University, Nanchang, China; 14 Nightingale-Saunders Clinical Trials and Epidemiology Unit, Kings Clinical Trials Unit, King’s College London, London, UK; 15 Faculty of Life Sciences and Medicine, King’s College London, London, UK

## Abstract

**Importance:**

Pulmonary rehabilitation (PR) improves exercise tolerance, symptom burden, and health-related quality of life for people with chronic respiratory conditions. However, demand for PR outstrips supply. Traditionally, PR has been delivered using specialist, gym-based exercise equipment.

**Objective:**

To investigate whether PR using minimal equipment (PR-min) is noninferior to PR using specialist gym exercise equipment (PR-gym).

**Design, Settings, and Participants:**

This parallel, 2-group, assessor- and statistician-blinded, noninferiority randomized clinical trial compared PR-min with PR-gym. Eligible participants were people with chronic respiratory disease referred for PR to the Regional Pulmonary Rehabilitation Unit in northwest London, UK. Recruitment occurred from October 15, 2018, to December 21, 2021, with a final follow-up to December 14, 2022. Randomization was by an independent web-based system using minimization with 1:1 allocation. Data analysis was performed from May 2023 to January 2025.

**Interventions:**

Both PR programs comprised 2 in-person, outpatient supervised sessions per week for 8 weeks. PR-min used minimal equipment (eg, walking circuit and body weight exercises), whereas PR-gym used specialist exercise equipment (eg, treadmills and weights machines).

**Main Outcomes and Measures:**

The primary outcome was change in incremental shuttle walk (ISW) distance after PR (ie, at 8 weeks; with a predefined noninferiority margin of −24 m). Secondary outcomes included dyspnea, health-related quality of life, costs, and adverse events.

**Results:**

A total of 436 participants (median [IQR] age, 71.7 [63.2-77.7] years; 239 [54.8%] male) were enrolled, with 218 randomized to PR-min and 218 to PR-gym. At 8 weeks, PR-min (n = 136) and PR-gym (n = 130) demonstrated significant improvements in ISW distance with no significant between-group difference in ISW distance change (mean, 1.7 m; 1-sided 97.5% CI lower bound, −16.8), which was within the −24-m noninferiority margin. The intention-to-treat analysis and a robust range of sensitivity analyses all demonstrated that PR-min was noninferior to PR-gym. Similar findings were observed for dyspnea and health-related quality of life. No excess adverse events or costs were seen with intervention.

**Conclusions and Relevance:**

This randomized clinical trial found that PR-min demonstrated noninferiority to PR-gym for exercise capacity, dyspnea, and health-related quality of life. PR-min can expand the number of settings where PR can be provided, thus improving patient accessibility.

**Trial Registration:**

isrctn.org Identifier: ISRCTN16196765

## Key Points

**Question:**

Is outpatient pulmonary rehabilitation delivered using minimal equipment noninferior to pulmonary rehabilitation delivered using specialist gym exercise equipment?

**Findings:**

In this randomized clinical trial of 436 individuals, participants gained significant improvements in exercise capacity, breathlessness, and health-related quality of life with pulmonary rehabilitation delivered using minimal equipment that was noninferior to pulmonary rehabilitation delivered using specialist gym exercise equipment.

**Meaning:**

The results of this study suggest that pulmonary rehabilitation using minimal equipment produces similar benefits to pulmonary rehabilitation using specialist gym exercise equipment, which may expand the number of settings where pulmonary rehabilitation can be provided, potentially improving patient accessibility.

## Teaser

This randomized clinical trial of people with chronic respiratory disease referred for pulmonary rehabilitation examines whether an outpatient, in-person supervised pulmonary rehabilitation program delivered using minimal equipment was noninferior to an in-person supervised pulmonary rehabilitation program delivered using specialist gym exercise equipment.

## Introduction

Many individuals with chronic respiratory conditions report dyspnea and exercise intolerance despite optimal pharmacotherapy. Pulmonary rehabilitation (PR), an interdisciplinary program of exercise training and education, is widely recommended^1,2,3,4^ because it improves exercise tolerance, symptom burden, and health-related quality of life.^5^ Use of specialist exercise equipment, such as treadmills, cycle ergometers, and fixed-weight machines, has been considered the standard model of delivering PR.^6^ Advantages include teaching proper form and action, ability to train individual muscles, and facilitating exercise prescription and progression.^7^ However, routine access to specialist gym-based exercise equipment is unfeasible for some services, and the limited global capacity of PR is partly attributable to a paucity of infrastructure and funding.^8^ There is increasing interest in PR delivered using minimal exercise equipment because this might expand the number and flexibility of settings that could deliver PR, including nonmedical facilities and patients’ homes.

The relative efficacy of PR using minimal exercise equipment remains uncertain. A recent meta-analysis^9^ comparing exercise training delivered using minimal or specialist equipment demonstrated no between-group difference in exercise capacity among participants with chronic obstructive pulmonary disease (COPD). However, the analysis only included 5 small trials (n = 164), described as low quality. Of these trials, only 1 (n = 12 per arm) incorporated aerobic and resistance exercise training, considered core components of PR.^10^ Given the paucity of data around the efficacy and magnitude of benefit associated with PR delivered using minimal equipment, we aimed to test whether an outpatient, in-person supervised PR program delivered using minimal equipment (PR-min) was noninferior to the reference standard of an in-person supervised PR program delivered using specialist gym exercise equipment (PR-gym).

## Methods

The study was approved by the Health Research Authority and London Camden and Kings Cross Research Ethics Committee and prospectively registered. Written informed consent was obtained from all participants. The trial protocol has been previously published,^11^ and the version approved by the ethics committee is available in Supplement 1. We report following the Consolidated Standards of Reporting Trials (CONSORT) reporting guideline,^12^ and additional information is reported in Supplement 1. The statistical analysis plan is available in Supplement 2.

### Study Design and Participants

The study was a parallel, 2-group, assessor- and statistician-blinded, noninferiority, individually randomized clinical trial of PR-min and PR-gym. Potential participants were 18 years or older, living with chronic respiratory disease, and referred for PR to the Regional Pulmonary Rehabilitation Unit in northwest London, UK. Exclusion criteria were contraindication to moderate intensity physical exercise (eg, unstable cardiovascular disease), progressive cancer or neurologic disorder with expected life expectancy of less than 12 months, completed PR within previous 12 months, or unable to provide informed consent. Recruitment occurred from October 15, 2018, to December 21, 2021, with a final follow-up to December 14, 2022.

### Randomization and Masking

Eligible participants were randomized at the individual level with a 1:1 allocation ratio, using an independent web-based system, to receive the intervention (PR-min) or control (PR-gym). Randomization by minimization was used to balance previous PR completion, multiple deprivation index,^13^ and physical frailty.^14^ Owing to the nature of the interventions, participants and intervention supervisors were not blinded. However, all postintervention assessments were performed by a researcher blinded to group allocation. The trial statistician (C.G.) was blinded to group allocation during analysis. Randomization and masking are described in in eAppendix 1 in Supplement 3.

### Intervention

Both PR-min and PR-gym comprised an 8-week outpatient exercise and multidisciplinary self-management education program, with 2 supervised sessions each week, delivered according to national quality standards by the same health care team.^2,3,4^ Each supervised session, delivered by qualified health care professionals, involved 1 hour of individualized, progressive aerobic and resistance training plus 45 minutes of education.

PR-gym was considered the standard clinical practice.^2,3,4^ For the exercise training component, participants underwent an individually prescribed and progressive program using gym-based equipment, such as treadmills, cycle ergometers, specialist lower limb resistance equipment (eg, leg press fixed-weight machine), and free weights. PR-min involved an individually prescribed and progressive training program using minimal equipment, such as walking circuit with stopwatch, body weight exercises, portable pedals or steppers, and elastic resistance bands. Both programs are described in eAppendix 1 in Supplement 3 and in the trial protocol in Supplement 1.^11^ Because this trial was a pragmatic clinical trial (designed to reflect typical clinical care settings), we allowed participants to switch to the alternative arm if a justifiable explanation was provided to the clinical team.

### Outcome Measures

Outcome measures were recorded at baseline assessment (visit 1), after PR at 8 weeks (visit 2), and after PR at 12 months (visit 3). The primary outcome was change in exercise capacity (incremental shuttle walk [ISW] test^15^) after PR (visit 1 to visit 2). The major secondary outcomes were change in dyspnea (Chronic Respiratory Questionnaire [CRQ]–Dyspnea),^16^ health-related quality of life (CRQ^16^), and adverse and serious adverse events. Additional secondary outcomes were change in ISW distance 12 months after PR (visit 1 to visit 3) and isometric quadriceps strength (quadriceps maximum voluntary contraction [QMVC]^17^). The EuroQol 5-Dimension 5-Level (EuroQol-5D-5L) survey^18^ and the modified Client Service Receipt Inventory^19^ were performed at visits 1, 2, and 3 to estimate costs and cost-effectiveness. Participant self-reported rating of change in their condition and PR program satisfaction (Global Rating of Change Questionnaire^20^) as well as adherence and compliance were measured at visit 2. More detail is given in eTable 1 in Supplement 3.

### Sample Size

At the time of trial design, the minimum important difference of the ISW distance was considered to be 47.5 m by international technical standards.^21,22^ The noninferiority margin was defined as half the known minimum important difference of the ISW distance (ie, 0.5 × 48.0 = 24.0 m) using the fixed-margin method with a preserved effect of 50% as recommended by previous guidance, including from the US Food and Drug Administration.^23,24^ Previous audit data from the regional PR center showed the ISW distance change SD with PR was 67 m. If there is truly no difference between PR-min and PR-gym, a minimum of 246 patients (123 in each group) was required to achieve 80% certainty that the lower limit of a 1-sided 97.5% CI (or equivalently a 95% 2-sided CI) would be above the noninferiority limit of −24.0 m. We anticipated a 32% rate of dropout from PR.^11^ Taking into account dropout, the original recruitment target was 362 patients (181 patients per group).

During the COVID-19 pandemic, the study was halted for 13 months (February 2020 to March 2021). At the time of the suspension, 74 participants had been randomized but were unable to commence the intervention or attend any research assessments due to the visiting restrictions. An amendment to increase the sample size from 362 to 436 was approved on February 24, 2021. The impact of the pandemic on the trial is described in eAppendix 1 in Supplement 3.

### Economic Analysis

Health care costs were derived from National Health Service tariffs and the Personal Social Services Research Unit database,^25,26^ adjusted to the year 2022. All costs are given in pound sterling (GBP). At the time of writing, 1 GBP was approximately equal to 1.35 US dollars. Effects on costs and quality-adjusted life years (QALYs) were estimated using seemingly unrelated regressions with age, sex, Charlson Comorbidity Index score, baseline EuroQol-5D-5L, and baseline log-transformed costs as variables, using multiple imputation by chained equations and nonparametric bootstrapped estimates,^27^ with Stata code provided by Mutubuki et al.^28^ Incremental cost-effectiveness ratios and the probability of cost-effectiveness at willingness-to-pay thresholds from £20 000 to £40 000 per QALY were estimated.^29^ Further detail is given in eAppendix 1 in Supplement 3.

### Statistical Analysis

The main statistical analyses estimated the difference in mean primary and secondary outcomes between participants randomized to PR-gym and PR-min by intention-to-treat principle from visit 1 to 2 (primary end point) and visit 1 to 3 (secondary end point). Group differences were compared using 1-sided, independent-sample *t* tests or nonparametric equivalent as a primary analysis and a linear mixed model adjusting for age, trial arm, baseline Medical Research Council (MRC) dyspnea score, previous PR completion, multiple deprivation index, and physical frailty as a secondary analysis. One-sided significance level was set at *P* < .025.

In addition to the primary analysis, the effect of treatment receipt (per-protocol analysis: participants complied with the protocol and did not switch trial arms) and a complier-average causal-effect (CACE) were estimated. Sensitivity analyses included (1) generalized estimating equation-based analysis to estimate the treatment effects, adjusting for imbalance if the baseline group difference in the percentage of smokers, participants younger than 70 years, or women was greater than 20%; (2) primary COPD diagnosis; (3) participants in the upper quartile for baseline ISW distance; and (4) complete case. More details are given in eAppendix 1 in Supplement 3. Data analysis was performed from May 2023 to January 2025.

## Results

**Figure 1.**
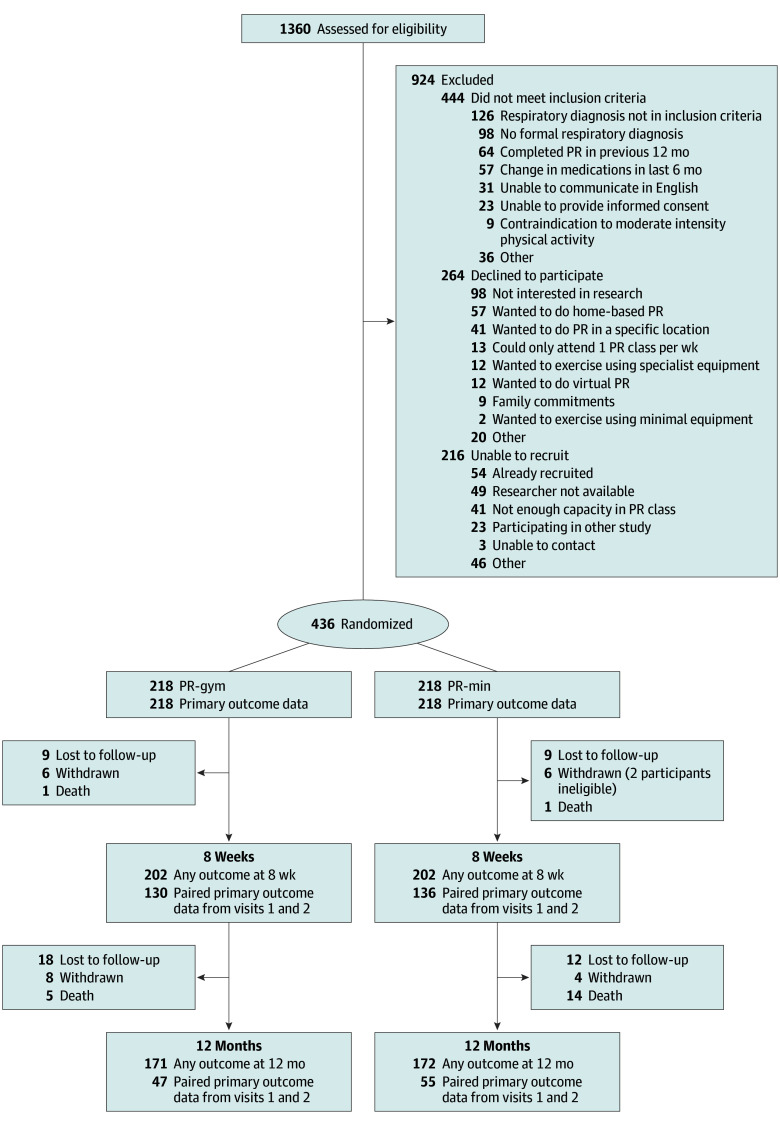
Consolidated Standards of Reporting Trials Flowchart Data are reported for those who returned any outcome data at research visits as well as those who provided primary outcome data. The primary outcome was the incremental shuttle walk test, which is a face-to-face supervised test. This test was not possible for a proportion of patients due to COVID-19 restrictions. PR indicates pulmonary rehabilitation; PR-gym, PR using specialist gym exercise equipment; PR-min, PR using minimal equipment.

A total of 1360 people were assessed for eligibility, of whom 436 (median [IQR] age, 71.7 [63.2-77.7] years; 239 [54.8%] male and 197 [45.2%] female) consented to participate, with 218 randomized to the PR-min arm and 218 to the PR-gym arm (Figure 1). Baseline characteristics are reported in Table 1. Participants were diagnosed with COPD (273 [62.6%]), interstitial lung disease (66 [15.1%]), asthma (61 [14.0%]), or bronchiectasis (36 [8.3%]). Participants had a median (IQR) body mass index (calculated as weight in kilograms divided by height in meters squared) of 26.8 (23.3-32.0), MRC dyspnea score of 3.1 (1.0, 4.0), and ISW distance of 260.0 (155.0-370.0) m. Research assessments, in-person or remote, were performed in 404 (92.7%) and 343 (78.7%) participants at visits 2 and 3, respectively, with similar attrition rates in PR-min and PR-gym (Figure 1).

**Table 1.** Baseline Characteristics of the Study Participants^a^

Characteristic	PR-min (n = 218)	PR-gym (n = 218)
Minimization criteria		
Previous PR completion	89 (40.8)	88 (40.4)
Multiple Deprivation Index decile, median (IQR)^b^	7.0 (5.0-9.0)	6.0 (4.0-9.0)
Frail (SPPB<10)	137 (62.8)	137 (62.8)
Sex		
Male	126 (57.8)	113 (51.8)
Female	92 (42.2)	105 (48.2)
Age, mean (SD), y	69.6 (11.0)	70.5 (10.5)
Weight, mean (SD), kg	76.4 (20.6)	77.1 (20.6)
BMI, mean (SD)	27.7 (6.9)	28.4 (6.9)
Smoking status		
Current	34 (15.6)	23 (10.6)
Previous	138 (63.3)	148 (67.9)
Never	46 (21.1)	47 (21.6)
Pack-year history, median (IQR)	25.0 (3.0-45.0)	25.0 (3.0-43.0)
Primary respiratory diagnosis		
COPD	137 (62.8)	136 (62.4)
ILD	34 (15.6)	32 (14.7)
Asthma	33 (15.1)	28 (12.8)
Bronchiectasis	14 (6.4)	22 (10.1)
Spirometry, mean (SD)		
FEV_1_, L	1.46 (0.64)	1.46 (0.63)
FEV_1_, % predicted	60.4 (23.5)	61.4 (24.8)
FVC, L	2.57 (0.90)	2.51 (0.80)
FVC, % predicted	84.8 (23.9)	83.9 (25.9)
Long-term oxygen therapy	11 (5.0)	9 (4.1)
Ambulatory oxygen therapy	15 (6.9)	23 (10.6)
Exacerbations requiring medication change in the past year, median (IQR)	1.0 (1.0-2.0)	1.0 (1.0-2.0)
Participants with at least 1 respiratory hospitalization in the past year	48 (22.0)	66 (30.3)
Charlson Comorbidity Index, median (IQR)^c^	1.0 (1.0-2.0)	1.0 (1.0-2.0)
MRC dyspnea score, mean (SD)^c^	3.2 (1.0)	3.1 (1.0)
MRC dyspnea score		
1	4 (1.8)	5 (2.3)
2	59 (27.1)	58 (26.6)
3	69 (31.7)	82 (37.6)
4	70 (32.1)	49 (22.5)
5	16 (7.3)	24 (11.0)
SPPB, mean (SD)^b^	9.9 (2.4)	9.6 (2.8)
ISW distance, median (IQR), m^b^	260.0 (160.0-380.0)	250.0 (150.0-360.0)
CRQ scores, mean (SD) ^b^		
Total	3.9 (1.0)	3.9 (1.1)
Dyspnea	.9 (1.1)	2.9 (1.1)
Fatigue	3.4 (1.3)	3.5 (1.3)
Emotion	4.5 (1.3)	4.5 (1.3)
Mastery	4.6 (1.4)	4.7 (1.4)
QMVC, mean (SD), kg^b^	28.4 (11.7)	26.8 (9.8)
EuroQol-5D-5L Utility Index score, mean (SD)^b^	0.8 (0.2)	0.7 (0.2)
EuroQol-5D-5L visual analog scale score, mean (SD)^b^	63.7 (19.8)	61.3 (19.3)

Abbreviations: BMI, body mass index (calculated as weight in kilograms divided by height in meters squared); COPD, chronic obstructive pulmonary disease; CRQ, Chronic Respiratory Questionnaire; EuroQol-5D-5L, EuroQol 5-Dimension 5-Level; FEV_1_, forced expiratory volume in 1 second; FVC, forced vital capacity; ILD, interstitial lung disease; ISW, incremental shuttle walk; MRC, Medical Research Council; PR, pulmonary rehabilitation; PR-gym, PR using specialist gym exercise equipment; PR-min, PR using minimal equipment; QMVC, isometric quadriceps maximum voluntary contraction; SPPB, short physical performance battery.

^a^ Data are reported as number (percentage) of participants unless otherwise indicated.

^b^ Higher score is better.

^c^ Higher score is worse.

### Primary Outcome

Primary outcome data were available for 266 participants (Figure 1). Missing data and dropouts were not associated with baseline age, sex, diagnosis, exacerbation frequency, smoking history, frailty, index of multiple deprivation, or trial group and were considered missing at random.

**Figure 2.**
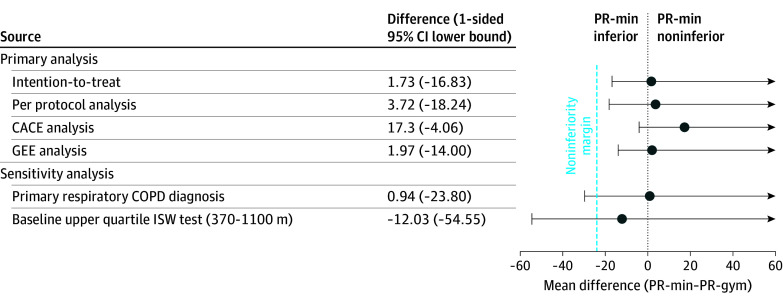
Noninferiority Graphs of the Primary and Sensitivity Analyses at Visit 2 CACE indicates complier average causal effects; COPD, chronic obstructive pulmonary disease; GEE, generalized estimating equation; ISW, incremental shuttle walk; PR, pulmonary rehabilitation; PR-gym, PR using specialist gym exercise equipment; PR-min, PR using minimal equipment.

After PR, there were significant improvements in ISW distance in both trial arms but no significant between-group difference in ISW distance change (mean, 1.7 m; 1-sided 97.5% CI lower bound, −16.8) (Table 2). The result remained after adjusting for age, trial arm, baseline MRC dyspnea score, previous PR completion, Multiple Deprivation Index, and short physical performance battery (eTable 2 in Supplement 3). PR-min was noninferior but not superior to PR-gym because the 97.5% CI lower bound did not cross the noninferiority margin of −24.0 m (*P* = .003) (Figure 2). The result remained similar in the per-protocol analysis, CACE analysis, and generalized estimating equation (Figure 2). Generalized estimating equation data are reported in eTable 3 in Supplement 3.

**Table 2.** Change in Primary Outcome (ISW Test) Between Visits 1 and 2^a^

Variable	ISW distance, m		
	Within group difference (95% CI)		Between group difference (1-sided 97.5% CI lower bound)
	PR-min	PR-gym	
Intention-to-treat analysis	24.7 (10.0 to 39.5)	23.0 (11.9 to 34.2)	1.7 (−16.8)
Per-protocol analysis	25.9 (6.0 to 45.8)	22.2 (20.3 to 34.1)	3.7 (−18.2)
CACE analysis	38.6 (21.0 to 56.2)	8.5 (−9.7 to 26.7)	30.1 (−0.5)
Sensitivity analysis of primary outcome (intention-to-treat analysis)			
Participants with primary respiratory diagnosis of COPD	24.9 (5.9 to 43.8)	24.0 (8.6 to 39.3)	0.9 (−23.8)
Baseline upper quartile ISW distance (range, 370-1100 m)	19.6 (−14.4 to 53.5)	31.6 (−6.9 to 56.3)	−12.0 (−54.6)

Abbreviations: CACE, complier average causal effects; COPD, chronic obstructive pulmonary disease; ISW, incremental shuttle walk; PR, pulmonary rehabilitation; PR-gym, PR using specialist gym exercise equipment; PR-min, PR using minimal equipment.

^a^ Generalized estimating equation–based analysis data are presented in eTable 3 in Supplement 3.

The sensitivity analysis of participants with a primary respiratory diagnosis of COPD demonstrated that PR-min was noninferior to PR-gym (n = 89 in the PR-min arm and 76 in the PR-gym arm; mean, 0.9 m; 1-sided 97.5% CI lower bound, −23.8) (Figure 2). However, for participants in the upper quartile of ISW distance at baseline (370-1100 m), noninferiority could not be demonstrated (Figure 2).

### Secondary Outcomes

At visit 2, PR-min was noninferior but not superior to PR-gym for CRQ (eFigure 1 in Supplement 3). For QMVC, for which there were more missing data, noninferiority was not demonstrated (eFigure 1 in Supplement 3).

In response to the Global Rating of Change Questionnaire, a total of 151 of 182 participants (83.0%) and 128 of 173 participants (73.5%) in PR-min and PR-gym arms, respectively, reported feeling “much better” or “a little better” after PR. Similarly, 174 of 181 (96.1%) and 165 of 173 (95.4%) in PR-min and PR-gym arms, respectively, reported being “very satisfied” or “satisfied” with their respective program. At visit 3, PR-min was noninferior but not superior to PR-gym for ISW distance and CRQ (eFigure 2 in Supplement 3) when analyzed in the same way as for the primary end point. For QMVC, noninferiority of PR-min to PR-gym was not demonstrated. The between-group difference in secondary outcomes and trend of change in ISW distance, CRQ-Total and CRQ-Dyspnea scores, and QMVC according to trial arm is given in Table 3 and eFigure 3 in Supplement 3.

**Table 3.** Between-Group Difference in Secondary Outcomes Between Visits 1 and 2

Outcome	Between-group difference, mean (95% CI)	Noninferiority margin
CRQ-Total^a^	0.2 (−0.3 to 0.0)	−0.25
CRQ-Dyspnea^a^	0.3 (−0.0 to 0.5)	−0.25
CRQ-Fatigue^a^	0.1 (−0.1 to 0.3)	−0.25
CRQ-Emotion^a^	0.1 (−0.1 to 0.3)	−0.25
CRQ-Mastery^a^	0.2 (−0.03 to 0.4)	−0.25
QMVC, kg^a^	−1.2 (−3.0 to 0.7)	−1.25

Abbreviations: CRQ, Chronic Respiratory Questionnaire; QMVC, isometric quadriceps maximum voluntary contraction.

^a^ Higher score is better.

### Economic Evaluation

PR-min was associated with lower costs (mean, –£218; 95% CI, −£1640 to £1205) and more QALYs (mean, 0.002; 95% CI, −0.005 to 0.009) at visit 2, but both estimates had a high level of uncertainty. At visit 3, participants in the PR-min arm had higher costs (mean, £1399; 95% CI, −£5913 to £8710) and more QALYs (mean, 0.003; 95% CI, −0.040 to 0.046), again with high levels of uncertainty. Additional details are given in eAppendix 3 and eTables 4 to 6 in Supplement 3.

### Intervention Adherence 

For the intention-to-treat analysis, the mean (SD) number of PR sessions attended (of a possible 16) was 8.9 (0.4) in the PR-min arm and 8.3 (0.4) in the PR-gym arm. Of the 404 participants who attended visit 2, 289 (71.5%) completed PR (attended ≥8 sessions and visit 2) with those allocated to PR-min and PR-gym attending a mean (SD) of 12.2 (0.3) and 11.8 (0.3) sessions, respectively.

As a pragmatic trial, we permitted individual participants to move arms in discussion with the clinical team. After randomization, we observed that 111 participants switched to receive the alternative arm (eTable 8 in Supplement 3). Of these, most made this decision based on practical reasons (location and scheduling), but 29 participants in the PR-min arm switched due to a preference for PR-gym, whereas 2 participants in PR-gym switched due to preference for PR-min. Baseline characteristics of switchers and nonswitchers are detailed in eTable 9 in Supplement 3. Further information about intervention adherence is given in eTables 7 and 9 in Supplement 3. We performed per-protocol and CACE analyses alongside the primary intention-to-treat analysis, confirming noninferiority despite the movement from one treatment arm to the other.

### Safety Data

The number of adverse events (and participants who experienced them) were comparable between groups: 260 (111 participants) in the PR-min arm and 272 (127 participants) in the PR-gym arm. Respiratory events (eg, chest infection not requiring hospitalization) were the most common adverse event: 176 (76 participants) in the PR-min arm and 184 (73 participants) in the PR gym arm. A total of 2 (0.7%) and 8 (2.9%) adverse events were related to the intervention in the PR-min and PR-gym arms, respectively (eTable 9 in Supplement 3). Similarly, the number of serious adverse events were comparable: 75 (37 participants) in the PR-min arm and 78 (39 participants) in the PR-gym arm. Exacerbations requiring hospitalization were also comparable: 40 (19 participants) in the PR-min arm and 37 (13 participants) in the PR-gym arm. Only 1 serious adverse event (1.3%) was related to PR-gym, with none related to PR-min (eTable 10 in Supplement 3). There were 21 deaths during the trial period, with 15 in the PR-min arm and 6 in the PR-gym arm (*P* = .045). These deaths predominantly occurred toward the end of follow-up (eFigure 4 and eTable 11 in Supplement 3).

## Discussion

In this randomized clinical trial, participants gained improvements in exercise capacity, breathlessness, and health-related quality of life with PR-min that were noninferior to those observed with PR-gym. A previous meta-analysis^9^ compared exercise training using minimal equipment with that delivered using specialist equipment in COPD and demonstrated no short-term difference in exercise capacity (6-Minute Walk Distance; 5 trials, n = 164), CRQ-Dyspnea (3 trials, n = 85), CRQ-Fatigue (3 trials, n = 53), or knee extensor strength (3 trials, n = 82). However, only small low-quality trials were included, which led to uncertainty about data generalizability. Furthermore, in only one small trial (n = 12 per arm)^10^ did the intervention incorporate aerobic and resistance training, considered core PR components,^30^ and any longitudinal data, albeit until only 14 weeks after intervention. A previous propensity-matched analysis^20^ involving participants with COPD, also demonstrated short-term noninferiority of PR-min to PR-gym for ISW distance, CRQ-Dyspnea, CRQ -Mastery, and CRQ -Total scores but not CRQ-Fatigue or CRQ -Emotion scores.

The current study is, to our knowledge, the largest and most rigorously conducted randomized clinical trial on the efficacy of PR-min. Its strengths include a prepublished protocol and oversight from an independent clinical trials unit. The PR interventions were designed and conducted according to international guidelines^3,30,31^ in a clinical PR service with standard operating procedures for exercise prescription and progression, which ensures intervention replicability. In contrast to previous studies,^9,10^ we included participants diagnosed with chronic respiratory diseases, which may enhance the generalizability of our results. Although PR practitioners could not be blinded due to the nature of the intervention, assessors were not involved in intervention delivery, with both assessors and statisticians blinded. We performed multiple and robust analyses of the primary outcome with consistent findings, which provides confidence in the validity of the results. The study is also the first to date to provide longitudinal data up to 12 months, demonstrating that PR-min is noninferior to PR-gym in both the short and long terms. Follow-up included collection of adverse events and cost-effectiveness data.

The World Health Organization’s Rehabilitation 2030 Initiative calls for global action to upscale rehabilitation.^32^ An international consortium listed investigation of rehabilitation strategies as a research priority to address the global and increasing burden of COPD and other respiratory diseases,^33^ whereas the need to increase PR access and capacity globally has long been identified.^8^ PR-min does not require any specialist equipment; therefore, initial capital costs are reduced, which may be particularly pertinent to those setting up new services in underresourced areas. Furthermore, PR-min can be conducted in nongymnasium settings, which could expand the number of settings where PR is conducted, improving patient accessibility. By offering PR-min as part of a suite of program options, services may expand patient choice.^34^ Notably, no increased costs or adverse events were observed with PR-min.

### Limitations

This study has some limitations. For a 13-month period, the COVID-19 pandemic led to substantial restrictions on recruitment and face-to-face assessments. These factors contributed to missing data, particularly for outcomes that needed in-person supervision (eg, ISW distance and QMVC). However, we met the prespecified minimum sample size target of 246 patients for the primary outcome.

The ISW distance improvement for both groups was of smaller magnitude than observed in previous observational work^20^ but in line with previous PR randomized clinical trials^5,35,36^ that used ISW distance as an exercise outcome. A possible explanation might be the lower-than-expected adherence to PR, with a mean attendance of 8.9 and 8.3 sessions for PR-min and PR-gym, respectively. We hypothesize that reduced adherence may have been related to restrictions placed by the COVID-19 pandemic.

There were a small number of deaths in both trial arms, with a statistically higher number occurring in the PR-min arm. Most of these deaths occurred at the end of the follow-up period and therefore were unlikely to be directly attributable to the intervention. Nevertheless, future research should monitor for any clear difference in mortality signal.

Despite the demonstration of noninferiority in this trial, there are some caveats to PR-min. Noninferiority of PR-min was not shown in the sensitivity analysis of participants with higher functioning capacity (upper quartile of baseline ISW distance). Although this finding could have been due to insufficient power, it is also possible that this might be due to difficulties eliciting physiologic improvements in higher-functioning individuals without specialist equipment; this finding is deserving of further investigation. Similarly, PR-min was not noninferior for QMVC. The trial was underpowered for this outcome, particularly because there were missing data, but it is plausible that PR-min may not provide sufficient resistance training stimulus without specialist resistance equipment.

A larger number of participants randomized to PR-min moved to PR-gym than vice versa (eTables 8 and 9 in Supplement 3). Future qualitative research is warranted to explore patient preferences for different PR models to personalize care.

## Conclusions

In this randomized clinical trial, we found that PR-min was associated with benefits in exercise capacity and health-related quality of life that were noninferior to those associated with PR-gym. This noninferiority persisted through 1 year, with no associated additional costs. PR-min should be offered as an option for PR delivery and can improve accessibility by expanding the number of settings where PR can be provided.

## Notes

Supplement 1.
Trial Protocol

Supplement 2.
Statistical Analysis Plan

Supplement 3. **eAppendix 1.** Supplementary methods **eTable 1.** Study outcome measures **eAppendix 2.** Supplementary results **eTable 2.** Primary outcome (ISW): Linear mixed model adjusting for age, dyspnea and the stratification factors (previous PR completion, multiple deprivation index, frailty) **eTable 3.** Generalised estimating equation-based analysis for incremental shuttle walk test between visits 1 and 2 **eFigure 1.** Non-inferiority graph at visit 2 for Chronic Respiratory Questionnaire a) Total, b) Dyspnea domain, c) Fatigue domain, d) Emotion domain, e) Mastery domain, f) QMVC **eFigure 2.** Non-inferiority graph at visit 3 for Chronic Respiratory Questionnaire a) Total, b) Dyspnea domain, c) Fatigue domain, d) Emotion domain, e) Mastery domain, f) QMVC **eFigure 3.** Graph of data over time by trial arm for a) incremental shuttle walk test, b) Chronic Respiratory Questionnaire-Total, c) Chronic Respiratory Questionnaire-Total-Dyspnea, d) QMVC **eAppendix 3.** Economic Analysis **eTable 4.** Mean costs at each visit, (2022 GBP), prior to multiple imputation **eTable 5.** Mean HRQoL at each visit, prior to multiple imputation **eTable 6.** Bootstrapped treatment effect estimates, PR delivered using minimal equipment versus PR gym **eTable 7.** Compliance with allocated trial arm **eTable 8.** Reasons participants did not comply with allocated trial arm **eTable 9.** Baseline characteristics of participants who switched or did not switch from their allocated treatment arm **eTable 10.** Number of adverse events and serious adverse events related to the intervention, by study arm **eFigure 4.** Kaplan-Meier curve demonstrating time to all-cause mortality during the trial according to trial arm **eTable 11.** Number of deaths by study arm and body system code
eReferences

Supplement 4.
Data Sharing Statement
