# Supplementary material for: Minimal vs Specialized Exercise Equipment for Pulmonary Rehabilitation: A Randomized Clinical Trial
Source: JAMA Netw Open. 2025 Aug 12;8(8):e2526616. doi: 10.1001/jamanetworkopen.2025.26616 (PMC12344533; doi:10.1001/jamanetworkopen.2025.26616)
Supplement: Supplement 1. — Trial Protocol [file jamanetwopen-e2526616-s001.pdf]

MInimal versus SpecialisT Equipment in the delivery of pulmonary Rehabilitation (MISTER): a randomised controlled trial

Short Study Title/Acronym: MISTER

REC Reference: 18/LO/0315
IRAS Reference: 241564

CHIEF INVESTIGATOR:

Dr. William Man
Consultant Chest Physician and Clinician Scientist
Department of Respiratory Medicine
Harefield Hosptial
Harefield
Middlesex
UB9 6JH

Phone: 01895 828851
Email: w.man@rbht.nhs.uk
Fax: 01895 828889

SPONSOR REPRESENTATIVE:

Mr Patrik Pettersson
Guy's and St Thomas' NHS Foundation Trust (GSTT)
Royal Brompton Hospital (RBH)
Research Office
Chelsea Wing, Level 2
Sydney Street
London SW3 6NP

Phone: 0207 352 8121 ext. 2610
Email: p.pettersson@rbht.nhs.uk
Fax: 0207 351 8829

Signature Page

The Chief Investigator (CI) and the Research Office have discussed and agreed this study protocol. The investigators agree to perform the investigations outlined in this study protocol and to abide by this protocol except in the case of medical emergency that will be notified to the Research Office.

The Investigator agrees to conduct the study in compliance with the study protocol and/or any subsequent amendments approved by the Sponsor and HRA, the Data Protection Act (1998), the Trust Information Governance Policy (or other local equivalent as applicable), the Research Governance Framework for Health & Social Care, 2nd Edition (2005), the Sponsor's SOPs, and any other applicable regulatory requirements.

This protocol has been written in accordance with the Sponsor's guidance for writing non-CTIMP protocols.

Table of contents

1. LIST OF ABBREVIATIONS 5
2. STUDY PERSONNEL AND FACILITIES 6
3. STUDY SYNOPSIS 8
4. INTRODUCTION 9
4.1 BACKGROUND 9
4.2 STUDY RATIONALE AND RISK/BENEFIT ANALYSIS 11
4.3 MANAGEMENT OF POTENTIAL STUDY RISKS 11
5. STUDY OBJECTIVES 11
5.1 PRIMARY OBJECTIVE 11
5.2 SECONDARY OBJECTIVES 12
6.1 OVERALL DESIGN 12
6.2 TREATMENT AND RATIONALE 13
6.3 SCHEMATIC OF STUDY DESIGN 15
7. ELIGIBILITY CRITERIA 16
7.1 INCLUSION CRITERIA 16
7.2 EXCLUSION CRITERIA 16
7.3 DISCONTINUATION/WITHDRAWAL OF PARTICIPANTS AND STOPPING RULES 16
8. SUBJECT/PATIENT RECRUITMENT PROCESS 16
9. STUDY PROCEDURES 17
9.1 INFORMED CONSENT 17
9.2 RANDOMISATION PROCEDURE 18
9.3 EMERGENCY UN-BLINDING 18
10. STUDY ASSESSMENTS 18
11. SAFETY REPORTING 22
11.1 DEFINITIONS 22
11.2 RECORDING ADVERSE EVENTS (AES) 22
11.3 ASSESSMENT OF SAES 23
11.4 REPORTING OF SAES TO THE SPONSOR AND THE REC 23
11.5 THE TYPE AND DURATION OF THE FOLLOW-UP OF SUBJECTS AFTER AES 24
11.6 PREGNANCY 24
11.7 ANNUAL PROGRESS REPORTS (APRS) 24
11.8 REPORTING URGENT SAFETY MEASURES 24
12. DATA MANAGEMENT AND QUALITY ASSURANCE 25
12.1 CONFIDENTIALITY 25
12.2 DATA COLLECTION TOOL 26

12.3 DATA HANDLING AND ANALYSIS **26**
12.4 ARCHIVING ARRANGEMENTS **27**
13. **STATISTICAL DESIGN** **27**
13.1 SAMPLE SIZE AND RECRUITMENT **28**
13.2 ENDPOINTS **28**
13.2.1 Primary endpoints 28
13.2.2 Secondary endpoints 28
13.3 STATISTICAL ANALYSIS PLAN **29**
13.3.1 Primary endpoint analysis 30
13.3.2 Secondary endpoint analysis 30
13.4 RANDOMISATION **31**
14. **COMMITTEES INVOLVED IN THE STUDY** **31**
15. **MONITORING AND AUDITING** **32**
16. **DIRECT ACCESS TO SOURCE DATA** **32**
17. **ETHICS AND REGULATORY REQUIREMENTS** **32**
18. **FINANCE** **33**
19. **INSURANCE AND INDEMNITY** **33**
20. **PUBLICATION POLICY** **33**
21. **STATEMENT OF COMPLIANCE** **33**
22. **LIST OF PROTOCOL APPENDICES** **35**
23. **REFERENCES** **100**

1. LIST OF ABBREVIATIONS

6MWT	Six minute walk test
95% CI	95% Confidence Interval
AE	Adverse Event
APR	Annual Progress Report
BTS	British Thoracic Society
CI	Chief Investigator
COPD	Chronic Obstructive Pulmonary Disease
CRF	Case Report Form
CRQ	Chronic Respiratory Questionnaire
CRQ-D	CRQ-Dyspnoea domain
CRQ-T	CRQ-Total score
CTIMP	Clinical Trial of an Interventional Medicinal Product
CTU	Clinical Trials Unit
EQ5D5L	Euro-Quol – 5 Dimensions – 5 Levels
FEV_1	Forced Expiratory Volume in One Second
FVC	Forced Vital Capacity
GCP	Good Clinical Practice
GRCQ	Global Rate of Change Questionnaire
HRA	Health Research Authority
ICER	Incremental Cost Effectiveness Ratio
ICF	Informed Consent Form
ILD	Interstitial Lung Disease
ISW	Incremental Shuttle Walk test
MCID	Minimum Clinically Important Difference
MRC	Medical Research Council
NMB	Net Monetary Benefits
PI	Principal Investigator
PIS	Participant Information Sheet
PR	Pulmonary Rehabilitation
PR-min	PR delivered using minimal equipment
PR-specialist	PR delivered using specialist equipment
QALY	Quality Adjusted Life Year
QMVC	Quadriceps Maximum Voluntary Contraction
RCT	Randomised Control Trial
REC	Research Ethics Committee
SAE	Serious Adverse Event
SOP	Standard Operating Procedure
SMF	Study Master File
SPPB	Short Physical Performance Battery
UK	United Kingdom

2. STUDY PERSONNEL AND FACILITIES

Principal Investigator (PI): Dr William Man
Department of Respiratory Medicine
Harefield Hospital
Harefield
Middlesex
UB9 6JH
E-mail: w.man@rbht.nhs.uk
Phone: 01895 828851

Statistician: Dr. Francesca Fiorentino
Cicely Saunders Institute
Division of Palliative Care, Policy and Rehab
Kings College London
London
SE5 9RJ
E-mail: francesca.fiorentino@kcl.ac.uk
Phone: 0207 8485242

Project Manager: Ms. Claire Nolan
Department of Respiratory Medicine
Harefield Hospital
Harefield
Middlesex
UB9 6JH
E-mail: c.nolan@rbht.nhs.uk
Phone: 01895 828851

Trial Co-ordinator for Kings Clinical Trials Unit:
Dr. Matthew Maddocks
Cicely Saunders Institute
Division of Palliative Care, Policy and Rehab
Kings College London
London
SE5 9RJ
E-mail: matthew.maddocks@kcl.ac.uk
Phone: 0207 8485242

PPI representative: Mrs. Nannette Spain
41 Gerrard Row
Harrow
Middlesex
HA1 2NE
E-mail: jonesse@ntlworld.co.uk
Phone: 0208 5372835

Clinical Lead for Hillingdon Integrated Respiratory Service:
Dr. Samantha Kon
Department of Respiratory Medicine
The Hillingdon Hospital NHS Foundation Trust
Hillingdon
UB8 3NN
E-mail: s.kon@rbht.nhs.uk
Phone: 01895 238282

3. STUDY SYNOPSIS

**Full study title:**	MInimal versus SpecialisT Equipment in the delivery of pulmonary Rehabilitation (MISTER): a randomised controlled trial
**Short study title:**	MISTER
**Study R&D number:**	241564
**Chief Investigator:**	Dr. William Man
**Medical condition/disease under investigation:**	Chronic respiratory disease
**Study duration:**	60 months
**Clinical phase:**	2 months
**Primary Outcome:**	• Change in incremental shuttle walk test (ISW) following pulmonary rehabilitation (PR)
**Secondary Outcomes:**	• Change in Chronic Respiratory Questionnaire Dyspnoea (CRQ-D) domain, CRQ total (CRQ-T), and quadriceps strength (QMVC) following PR<br>• Change in ISW, CRQ-D, CRQ-total, and QMVC from pre-PR to 12 months post-PR completion<br>• Health economic analysis
**Study population:**	Patients with stable chronic respiratory disease referred for pulmonary rehabilitation with Medical Respiratory Council (MRC) dyspnoea scale of two or more.
**Recruitment Target**	436 patients
**Recruitment Window (Months)**	29 months
**Methodology:**	This study is a parallel, two-group, assessor- and statistician-blinded, non-inferiority randomised trial. Participants will be randomised using minimization at the individual level with a 1:1 allocation to either PR delivered using minimal exercise equipment (PR-min: intervention) versus PR delivered using specialist exercise equipment (PR-specialist: control).
**Eligibility criteria:**	***Inclusion criteria:***<br>1. Adults > 18 years of age, either sex.<br>2. Physician diagnosis of stable chronic respiratory disease, typically COPD, interstitial lung disease, bronchiectasis, chronic asthma or chest wall disease.<br>3. Referred for PR in line with British Thoracic Society guidelines (i.e. ambulatory – can walk ≥5 metres, functional impairment related to breathlessness, typically MRC dyspnoea score ≥2).<br>4. Able to communicate verbally and respond to questions in written English
	Exclusion criteria:<br>1. Contra-indication to moderate intensity physical exercise e.g. unstable cardiovascular disease.

	2. Progressive cancer or neurological disorder with expected life expectancy less than 12 months.<br>3. Completed PR within previous 12 months.<br>4. Unable to provide informed consent.
**Study treatment:**	
PR delivered using minimal exercise equipment (PR-min – intervention) versus PR delivered using specialist exercise equipment (PR-specialist - control). Both interventions will comprise two supervised centre-based sessions per week for eight weeks, delivered by same PR team following the same standard operating procedures for exercise prescription and progression.	

4. INTRODUCTION

4.1 BACKGROUND

Pulmonary rehabilitation (PR) is an evidence-based exercise and education programme that is now widely accepted as a cornerstone of management for people with chronic obstructive pulmonary disease (COPD) and other chronic respiratory disorders.[1] The most recent Cochrane review, comprising 65 randomised controlled trials (RCTs) and 3822 participants, stated that no further trials comparing PR and standard care were necessary, or even ethical, as the evidence supporting the benefits of PR on exercise capacity and health-related quality of life were conclusive.[2] The majority of evidence to support PR has come from trials conducted in hospital or rehabilitation centres that utilise specialist exercise equipment such as treadmills, cycle ergometers, and specialist resistance equipment (PR-specialist).[2] However, in clinical practice, supply does not meet demand, and routine access to specialist exercise equipment may not be feasible.[3] In the 2015 Royal College of Physicians National Audit, it was identified that PR services in England and Wales received 68,000 referrals in 2014 out of an estimated 446,000 eligible patients with COPD.[3] Furthermore, 81% of PR programmes in England and Wales in 2015 were hosted in community sites and 59% of community PR programmes probably did not have access to specialist exercise equipment.[3] Accordingly, exercise training at these sites was completed with minimal exercise equipment (PR-min), typically using simple, portable equipment such as free weights, elastic resistance bands (eg.Theraband™), walking programmes, and bodyweight resistance exercises.[3]

Apart from improving accessibility, it has been argued that PR-min may have other advantages over PR-specialist. Exercise-training using minimal equipment may better reflect activities of daily-living than training using specialist equipment, and therefore be easier to replicate and maintain at home following discharge from PR. However, purists would argue that it is more difficult to prescribe (and progress) exercise training, particularly resistance training, without the use of specialist exercise equipment. This is particularly pertinent in higher functioning individuals.

We conducted a literature search, which identified a systematic review of eight RCTs that compared outcomes following exercise interventions for people with COPD completed with minimal exercise equipment with usual care.[4] Minimal exercise equipment was defined by the authors as exercises completed with portable or no equipment, such as resistance exercises with Theraband™ or a walking programme.[4] Usual care involved care without an exercise intervention. Results were conflicting for exercise outcomes. In four studies where the six minute walk test (6MWT) was used as the primary outcome measure of exercise capacity, the pooled effect showed a mean between-group difference of 40 metres (m) (95% confidence interval (95% CI): 13m to 67m) favouring the intervention group (n=90).[4] Conversely, in the other four studies (n=389) where the incremental shuttle walk test (ISW) was used as an outcome measure, there was no significant mean between-group difference: 21m (95% CI: -9m to 51m).[4] For health-related quality of life, results were similarly conflicting.[4] Whereas in the four studies that used the St. George's Respiratory Questionnaire there was a sizeable difference between the intervention and control groups (-7 (95% CI: -12 to -3) points), the mean between-group difference of the Chronic Respiratory Questionnaire dyspnoea (CRQ-D) and fatigue domains did not reach the minimum clinically important difference (MCID) in three other studies.[4] Only one of the eight RCTs measured muscle strength but results were only reported descriptively with no statistical testing.[4] Furthermore, only one of the eight RCTs would fulfil the British Thoracic Society (BTS) guidelines definition of PR,[1] with seven studies not offering any education component.[4]

To our knowledge, there have been no trials comparing centre-based PR using minimal and specialist exercise equipment. A previous National Institute for Health Research Health Technology Assessment-funded randomised 2x2 trial compared PR undertaken in community venues with PR undertaken in a hospital venue, in 240 patients with COPD.[5] Participants were block randomized (hospital n=129; community n=111). Both groups received twice-weekly PR for six weeks with the exercise training protocol identical in both venues. Importantly, neither community nor hospital sites had access to specialist exercise equipment. Patients in both groups improved their walking distance, exceeding the MCID of the endurance shuttle walk, and there were similar improvements in health related quality of life.[5] Although the results support the conclusion that the efficacy of PR with minimal equipment is not influenced by hospital or community locations, this study did not address whether PR with minimal equipment is non-inferior to 'gold standard' PR delivered using specialist exercise equipment. A recent trial from Australia compared home-based, lightly supervised PR with minimal access to exercise equipment with gold standard centre-based PR, and demonstrated equivalent outcomes.[6] However there was strong evidence of selection bias in that 42% of eligible participants were not randomised because of a preference for centre-based PR. Furthermore, the changes in the primary outcome (6MWT) following PR in both groups were considerably lower than has been observed in previous meta-analyses, or data from the UK national audit.

In summary, there is a paucity of robust literature examining the efficacy of centre-based PR completed with minimal exercise equipment. Previous studies have been significantly underpowered with large heterogeneity in the content and delivery of the exercise-training. Our

literature review lends some support for PR completed with minimal exercise equipment improving exercise capacity and quality of life compared with usual care without PR.

4.2 STUDY RATIONALE

No study has compared whether PR completed with minimal exercise equipment is non-inferior to PR completed with specialist exercise equipment. This is an important and current health service question given the increasing disparity between supply and demand of PR. If PR classes that use minimal equipment alone are found to be non-inferior to PR delivered using specialist equipment, this may help significantly increase provision of PR in community settings close to patients' home. However if classes that use minimal equipment are shown to be inferior, this would lead to a significant shake-up in national provision of PR; according to the last national audit in 2015, a significant proportion of PR delivered in England and Wales is from sites that have little access to specialist equipment.[3] Furthermore, this may affect future development of PR services, particularly in low-to-middle income countries.

4.3 MANAGEMENT OF POTENTIAL STUDY RISKS

This study is a non-clinical trial of an interventional medicinal product (non-CTIMP). Both arms of the trial comprise interventions that would be considered appropriate and adequate standard of care and does not involve any interventions or measurements that are likely to pose significant risk to the patient or investigator. Care will be taken to minimise and manage any distress generated by interviews and/or questionnaires.

5. STUDY OBJECTIVES

The aims of the research are to determine whether an eight-week supervised PR programme using minimal exercise equipment (PR-min) is non-inferior to a standard eight-week supervised PR programme delivered using specialist exercise equipment (PR-specialist) in terms of health benefits for patients with chronic respiratory disease.

5.1 PRIMARY OBJECTIVE

- To determine whether PR-min is non-inferior to PR-specialist regarding change in exercise capacity measured by the incremental shuttle walk test (ISW)[7] distance from baseline (Visit 1) to immediate post-PR assessment at eight weeks (Visit 2).

5.2 SECONDARY OBJECTIVES

- To determine whether PR-min is non-inferior to PR-specialist regarding changes in the following variables from baseline (Visit 1) to immediate post-PR assessment at eight weeks (Visit 2):
  - Change in breathlessness measured using the dyspnea domain of the Chronic Respiratory Questionnaire (CRQ-D).
  - Change in disease-specific health related quality of life measured using the CRQ total score (CRQ-T)
  - Change in lower limb muscle strength measured using isometric quadriceps maximum voluntary contraction (QMVC)

- To determine whether PR-min is non-inferior to PR-specialist regarding changes in the following variables from baseline (Visit 1) to 12 months after the post-PR assessment (Visit 3):
  - Change in breathlessness measured using the CRQ-D.
  - Change in disease-specific health related quality of life measured using the CRQ-T.
  - Change in lower limb muscle strength measured using isometric QMVC.

- To evaluate the trial process by recording the participant recruitment and retention, participant uptake of PR, PR attendance, PR completion, reasons for PR non-completion and participant satisfaction in each arm of the study, at the appropriate stage of the trial.

- To estimate the cost and cost-effectiveness during the trial period using:
  - The Modified Client Service Receipt Inventory questionnaire measured at visits 1, 2 and 3. Unit costs will be obtained from published sources and service use costs will be obtained by combining quantity in service use and unit costs of the service.
  - Quality-adjusted life years (QALYs) will be calculated using the Euro Qol-5 Dimensions-5 Levels measured (EQ5D5L) at visits 1, 2 and 3.
  - Data obtained from NHS Digital regarding health resource utilization between visit 1 and visit 3. This will include information on elective and non-elective hospital admissions (e.g. reason for admission; type of ward; treatment received; length of stay (including date of admission and discharge); location of discharge) as well as outpatient healthcare contacts (e.g. type of appointment, number of contacts, treatment received) and mortality (e.g. date of death, reason for death, place of death).

6.0 STUDY DESIGN

6.1 OVERALL DESIGN

This study is a parallel, two-group, assessor- and statistician-blinded, randomised trial. Participants will be randomised at the individual level with a 1:1 allocation to either PR-min or PR-specialist. Both interventions will comprise two supervised sessions per week for eight weeks delivered by the same team. Outcome measures will be recorded at initial assessment for PR (Visit 1), following PR at eight weeks (Visit 2) and at 12 months (Visit 3).

6.2 TREATMENT AND RATIONALE

PR-specialist (control group): The control intervention will be current gold-standard clinical practice. PR-specialist will comprise an eight-week outpatient exercise and multi-disciplinary self-management education programme, with two supervised and at least one additional home session each week, and delivered according to the British Thoracic Society Quality Standards.[8] Supervising staff will comprise specialist respiratory therapists with a minimum of two years' experience in PR. Available equipment will include treadmills, cycle ergometers, cross-trainers, specialist lower limb resistance equipment (such as leg press, knee extension). Each supervised session will consist of one hour of exercise (at least 30 minutes aerobic exercise) and 45 minutes of education. Study participants in the control group will have a choice of centres across northwest London.

Initial walking speed prescription on the treadmill will be 80% of predicted peak oxygen consumption based on baseline ISW performance[7], whilst initial endurance cycling will be initially set at 60% peak workload on a cycle ergometer with the aim of patients completing ten minutes of continuous training. Lower limb resistance training will comprise two sets of ten leg press repetitions on specialist resistance equipment performed with an initial training load of 60% one-repetition maximum. Similarly two sets of ten bilateral knee extension repetitions will be performed on specialist resistance equipment at an initial training load of 60% one-repetition maximum. This will be supplemented with sit-to-stand sets, plus knee lifts/extension and hip abduction with appropriate ankle weights up to 10kg. Upper limb resistance training will comprise biceps curls, shoulder press and upright row with free weights or Therabands™ (red to black). Exercise training will be individualised and regularly progressed (either in duration or intensity) according to standard operating procedures, with targets reviewed at each supervised session. Education will be delivered by a multi-disciplinary team with topics chosen to develop patients' understanding and holistic management of their disease.

PR-min (Intervention group): PR-min will also comprise an eight-week outpatient exercise and multidisciplinary self-management education programme, with two supervised and at least one additional home session each week, and delivered according to the BTS Quality Standards.[8] As for PR-specialist, supervising staff will comprise specialist respiratory therapists with at least two years' experience of delivering pulmonary rehabilitation independently. There will be no access to

treadmills, cycle ergometers, or specialist resistance equipment. Available exercise equipment will include portable steppers, portable pedals, hand and ankle weights (up to 5kg), and Therabands™ (red to black). Each supervised session will consist of one hour of exercise (at least 30 minutes aerobic exercise) and 45 minutes of education. Study participants in the intervention group will have a choice of centres across northwest London.

Initial walking speed prescription will be 80% of predicted peak oxygen consumption based on baseline ISW performance. Participants will be provided with their own stopwatch and given time targets to complete a walking course of known distance. Although the resistance of the portable steppers and pedals can be manually adjusted, this cannot be objectively quantified. Initial prescription will be set at "level 1" but individually adjusted to find an intensity where patients can complete ten minutes of continuous training with a target modified Borg breathless score of 3-4 and a Borg rating of Perceived Exertion of 13-15 (on a scale of 6-20). Resistance training will include functional activities such as sit-to-stand and step-ups as well as Theraband™ based exercises such as sitting knee extension, leg press and hip flexion as well as standing hip extension, squats, chest press and lateral raise. Progression will be through the use of hand/ankle weights, and increasing resistant Therabands™ (from red to black). Exercise training will be individualised and regularly progressed (either in duration or intensity) with targets reviewed at each supervised session. Education will be delivered by a multi-disciplinary team as per PR-specialist.

1.3 SCHEMATIC OF STUDY DESIGN

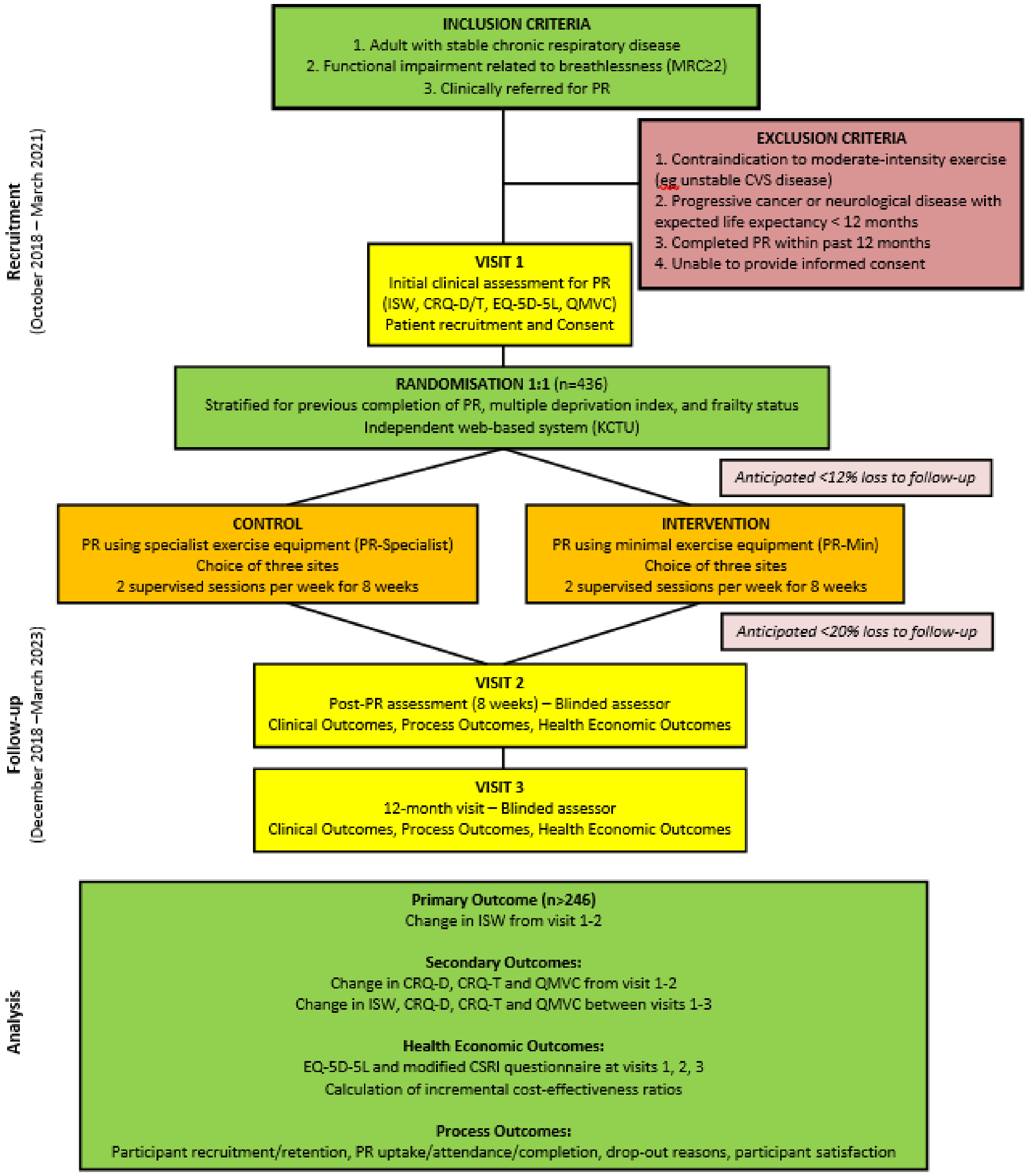

7.0 ELIGIBILITY CRITERIA

7.1 Inclusion criteria

1. Adults >18 years of age, either sex.
2. Physician diagnosis of stable chronic respiratory disease, typically COPD, interstitial lung disease (ILD), bronchiectasis, chronic asthma or chest wall disease.
3. Referred for PR in line with BTS guidelines (i.e. ambulatory – can walk ≥5 metres, functional impairment related to breathlessness, typically MRC dyspnoea score ≥2)
4. Able to communicate verbally and respond to questions in written English.

7.2 Exclusion criteria

1. Contra-indication to moderate intensity physical exercise e.g. unstable cardiovascular disease.
2. Progressive cancer or neurological disorder with expected life expectancy less than 12 months.
3. Completed PR within previous 12 months.
4. Unable to provide informed consent.

7.3 Discontinuation/withdrawal of participants and stopping rules

Participants should be withdrawn from the trial if it is deemed unsafe to exercise e.g. onset of unstable cardiac disease, musculoskeletal injury.

Should participants wish to withdraw from the trial, no further interventions would be carried out on the participant under the study protocol and no new personal data would be collected. Data already collected in relation to the participant may be retained and used for the purposes for which consent has already been given, provided they are effectively anonymised. All reasons for voluntary withdrawal from the study will be documented.

Participants who are withdrawn from the trial will not be replaced.

If the Chief Investigator (CI) deems that there are obvious safety concerns the trial will be terminated prematurely.

8.0 SUBJECT/PATIENT RECRUITMENT PROCESS

Patient recruitment at a site will only commence once the *study* team has ensured that the following approval/essential documents are in place:

1. Health Research Authority (HRA) approval,
2. Local Site Delegation of Duties and Signature Log is completed (if applicable).

All subjects who wish to enter the study will be fully screened and consented by the CI, Principal Investigator or someone else from the research team who is suitably qualified.

Participants will be chronic respiratory disease patients referred to the Harefield PR programme. Following clinical referral, but prior to initial assessment, potential participants will be approached by telephone and provided with a participant information sheet. Consent will take place following the pre-PR clinical assessment visit either in person or via telephone. The nominated researchers will be responsible for recruitment and consent.

9.0 STUDY PROCEDURES

9.1 INFORMED CONSENT

Informed consent will be obtained by the nominated researchers as recorded in the Sponsor's Delegation of Responsibilities Log. All individuals taking informed consent will have received consent training. All participants will provide written consent.

Consent to enter this study will be obtained after a full account has been provided of its nature, purpose, risks, burdens and potential benefits, and the patient has had the opportunity to deliberate. The patient will be allowed to specify the time they wish to spend deliberating, usually up to 24 hours.

Periods shorter than 24 hours will be permitted if the patient feels that further deliberation will not lead to a change in their decision, and provided the person seeking consent is satisfied that the patient has fully retained, understood and deliberated on the information given. This provision has been made with the support of our patient advisory group.

Likewise, periods longer than 24 hours will be permitted should the patient request this. The Investigator or designee will explain that the patients are under no obligation to enter the study and that they can withdraw at any time during the study, without having to give a reason.

A copy of the signed Informed Consent Form (ICF) along with a copy of the most recent approved Patient Information Sheet (PIS) will be given to the study participant. The original signed consent form will be retained at the study site (one filed in the medical notes and one field in the Study Master File (SMF)). A copy of the consent form will also be given to the patient.

If new safety information results in significant changes to the risk–benefit assessment, the consent form will be reviewed and updated if necessary. All subjects, including those already being

treated, will be informed of the new information, given a copy of the revised consent form and asked to re-consent if they choose to continue in the study.

9.2 RANDOMISATION PROCEDURE

- Randomisation procedure: Consenting participants will be randomised at the individual level with a 1:1 allocation, using an independent web-based system provided by the United Kingdom Clinical Research Collaboration registered King's Clinical Trials Unit (CTU), to receive either "usual care" (PR-specialist) or intervention (PR-min). Randomisation by minimization will ensure that participants will be stratified according to previous completion of PR (yes / no), multiple deprivation index (most deprived quintile of index: yes / no) and frailty status (Short Physical Performance Battery score < 10 / ≥10). A proportion of patients will be entered initially using simple randomisation in order to create a level of initial imbalance and the minimisation algorithm will maintain a level of randomness in order to preserve pre-randomisation allocation concealment. Once randomised, the system will automatically generate a full audit trail of the process and send emails to relevant investigators in a blinded or unblinded format, depending on their role. For each arm, participants will be provided with a choice of three sites to undertake PR.

- Blinding: Owing to the nature of the interventions, participants and providers of the intervention will not be blinded. However post-rehabilitation assessments will be performed by a researcher not involved in the delivery of either intervention arm, and blinded to group allocation. The trial statistician will also be blinded to group allocation.

9.3 EMERGENCY UN-BLINDING

It is not anticipated that an emergency code break will be required out of hours in this study. Within office hours, all requests for code break should be directed to the Chief Investigator (CI).

10.0 STUDY ASSESSMENTS

Some outcome measurements will be assessed as part of the patient's routine clinical assessment (**visit 1 – pre-PR assessment** and **visit 2 – post-PR assessment**); further measurements will occur at a third visit **(visit 3 - 12 months following visit 1)**. The summary chart of study assessments is in section 10.12.

10.1 STRUCTURED HISTORY (VISIT 1, VISIT 3)

A structured history will be taken **as part of routine clinical assessment**, including age, gender, previous hospitalisations (including bed days), past medical history/co-morbidities (including age-adjusted Charlson co-morbidity index[9]), symptoms of breathlessness (Medical Research Council dyspnoea score[10]), usual functional status, physical activity levels, drug history and social history. The history may be supplemented with records from hospital notes and GP records. This will take 10 minutes.

10.2 Anthropometry (Visit 1-3)

Height, weight and body mass index will be recorded **as part of routine clinical assessment**. This will take three minutes.

10.3 Spirometry (Visit 1, Visit 3)

All participants will undergo spirometry performing the Forced Vital Capacity (FVC) / Forced Expiratory Volume in one second (FEV_1) manoeuvre as described in Appendix 1, using an EasyOne™ diagnostic spirometer. As a minimum, three technically acceptable tests must be performed and must meet the Association of Respiratory Technology and Physiology reproducibility criteria.[11] This will occur **as part of routine clinical assessment**. Results obtained will include FVC, FEV_1 and the FEV_1/FVC ratio. Percent predicted reference equations from the European Respiratory Society/European Steel and Coal Society were used.[12] This will take 10 minutes.

10.4 Multiple Deprivation Index (Visit 1)

The multiple deprivation index of each participant will be calculated using the patients' home postcode based on data from 'The English Indices of Deprivation 2015'. This is a dataset published by the UK government accessed using this link: http://imd-by-postcode.opendatacommunities.org/ . The postcode is not saved on the website nor is any other identifiable data inputted onto this website.

10.5 Frailty (Visit 1-3)

All participants will perform the Short Physical Performance Battery (SPPB) which is a simple test of lower limb functional performance and a marker of frailty.[13] It comprises an assessment of standing balance, usual walking speed and ability to stand from a chair. The SPPB is scored out of 12 with a higher score indicating better functional performance. A score of <10 indicates a frail status and this cut-off will be used in the randomisation procedure. The SPPB takes five minutes to complete. The SOP is detailed in Appendix 2. This will occur as **part of routine clinical assessment.**

10.6 EXERCISE CAPACITY (VISIT 1-3)

The ISW is an incremental, externally paced, field walking test that involves participants walking around a 10 metre course in time to a series of progressively faster beeps played from a CD player. The test will occur **as part of routine clinical assessment.** At visit 1**,** two tests (a practice test and then the formal test) will be undertaken with at least 30 minutes rest between tests.[14] This is in accordance with international technical standards. At visits 2 and 3, no practice ISW tests will be performed. Measures of breathlessness, oxygen saturation levels and heart rate will be recorded before and after the test. The distance completed in metres will also be recorded. The SOP is described in Appendix 3 and the whole procedure takes 45 to 60 minutes.

10.7 DYSPNOEA (VISIT 1-3)

Dyspnoea will be measured using the CRQ-D **as part of routine clinical assessment**.[15] The dyspnoea domain allows the patients to choose five activities that have been limited by shortness of breath in the past two weeks.

10.8 HEALTH-RELATED QUALITY OF LIFE (VISIT 1-3)

Health-related quality of life will be measured using the CRQ **as part of routine clinical assessment**.[15] This 20 item questionnaire, is responsive to PR, and contains four domains; dyspnea (described above), fatigue, emotional function and mastery, and a total score. Each item is scored on a seven point Likert scale, with a lower score indicating a higher symptom burden. A copy of the CRQ is in Appendix 4 and it takes five minutes to complete.

10.9 MUSCLE STRENGTH (VISIT 1-3)

Isometric quadriceps maximal voluntary contraction of the dominant leg will be measured using specially designed chair and strain gauge. This test involves the participant pushing against an ankle strap with the knee positioned at 90^0.[16] A warm-up and six efforts will be performed. The SOP is in Appendix 5 and the test will take eight minutes to perform.

10.10 HEALTH ECONOMIC EVALUATION (VISIT 1-3)

- The Modified Client Service Receipt Inventory questionnaire will be used to record information on health care resource, medicine and equipment use, informal care provided by family members, time off work and costs borne by the patient and family relating to the chronic lung disease. A copy of the questionnaire is in Appendix 6 and it takes seven minutes to complete.
- The EQ5D5L is a generic measure of health status that comprises a visual analogue scale and five-item questionnaire with the following domains: mobility; self-care; usual activities; pain/discomfort and anxiety/depression.[17] It is measured **as part of routine**

clinical assessment. A copy of the questionnaire is in Appendix 7 and it takes two minutes to complete.

- After visit 3, data will be obtained from NHS Digital regarding health resource utilization between visit 1 and visit 3. This will include information on elective and non-elective hospital admissions (e.g. reason for admission; type of ward; treatment received; length of stay (including date of admission and discharge); location of discharge) as well as outpatient healthcare contacts (e.g. type of appointment, number of contacts, treatment received) and mortality (e.g. date of death, reason for death, place of death).

10.11 Safety and Trial Process Evaluation (Visit 1 - 2)

- Safety will be assessed in real-time using adverse event (AE) reporting.
- PR uptake, adherence and completion will be assessed objectively through PR attendance registers and training records. Reasons for non-completion of PR will be assessed objectively through PR records.
- To measure patient satisfaction, participants will rate their response to the following question *"How do you feel your overall condition has changed after rehabilitation?"* on a five point Global Rating of Change Questionnaire. The scale ranges from *"1: I feel much better"* to *"5: I feel much worse".* A copy of this questionnaire is in Appendix 8 and it takes less than one minute to complete.

10.12 Summary Chart of Study Assessments

	Visit 1	Visit 2	Visit 3
Structured history	X		X
Anthropometry	X	X	X
Spirometry	X		X
Multiple Deprivation Index	X		
Frailty	X	X	X
Exercise capacity	X	X	X
Dyspnoea	X	X	X

Health-related quality of life	X	X	X
Muscle strength	X	X	X
Health-economic evaluation	X	X	X and after visit 3 (NHS Digital)
Safety and trial process evaluation	X	X	X

11.0 SAFETY REPORTING

11.1 DEFINITIONS

Adverse Event (AE) — any untoward medical occurrence in a patient or clinical study subject who is administered a treatment and which does not necessarily have a causal relationship with this treatment (*i.e.* any unfavourable or unintended change in the structure (signs), function (symptoms), or chemistry (lab data) in a subject to whom a treatment/study procedure has been administered, including occurrences unrelated to that product/procedure/device).

Serious Adverse Event (SAE) – is defined as an untoward occurrence that:

- Results in death; or
- Is life-threatening (places the subject, in the view of the Investigator, at immediate risk of death)
- Requires hospitalization or prolongation of existing hospitalization (hospitalisation is defined as an inpatient admission, regardless of length of stay; even if it is a precautionary measure for observation; including hospitalisation for an elective procedure, for a pre-existing condition)
- Results in persistent or significant disability or incapacity (substantial disruption of one's ability to conduct normal life functions)
- Consists of a congenital anomaly or birth defect (in offspring of subjects or their parents taking the study drug regardless of time of diagnosis)
- Is otherwise considered medically significant by the investigator.

Important medical events that may not be immediately life-threatening or result in death or hospitalisation but may jeopardise the subject or may require intervention to prevent one of the outcomes listed in the definition of serious will also be considered serious.

11.2 RECORDING ADVERSE EVENTS (AEs)

All AEs will be recorded in the hospital notes and Case Report Form (CRF).
If the Investigator suspects that the disease has progressed faster due to the administration of the study treatment/procedure, then he/she will report this as an unexpected AE to the Sponsor and the REC as detailed in Section 12.3.

11.3 Assessment of SAEs

The CI and his research team at RBH are responsible for reporting events to the Research Office immediately and/or within 24 hours of becoming aware of the event using the Sponsor's SAE Reporting Form.

Classification and causality of AEs will be conducted by the project manager and reviewed by CI. The CI cannot downgrade the project manager's classification and if there is disagreement which cannot be resolved during formal discussion then the assessment of the project manager will be accepted. The CI, can however, upgrade the seriousness of an event without consultation with the project manager.

11.4 Reporting of SAEs to the Sponsor and the REC

All AEs that are to be reported to the Research Office must be recorded, signed and dated by the Investigator at site. Research Office accepts study specific SAE forms, HRA SAE Form or RB&H template SAE Reporting Form available here.

Information can be submitted in electronic format:

- E-mail: research.reporting@rbht.nhs.uk
- Fax: 0207 351 8829.

An SAE occurring to a research participant will be reported to the Research Ethics Committee (REC) that gave a favorable opinion of the study (the 'main REC'), the study Sponsor (RB&H Research Office) and the local R&D Office where in the opinion of the CI/PI the event was:

- **'related'**: that is, it resulted from administration of any of the research procedures; and
- **'Unexpected'**: that is, the type of event is not listed in the protocol as an expected occurrence.

Reports of related and unexpected SAEs will be submitted within 15 days of the CI becoming aware of the event; using the SAE reporting form for non-CTIMPs published on the HRA website and entitled non-CTIMP safety report to REC. The form should be completed in typescript and signed by the CI prior to submission to the REC.

Reports of SAEs in double-blind studies should be un-blinded.

The coordinator of the main REC will acknowledge receipt of safety reports within 30 days. It is the responsibility of the CI and his/her research team to send a copy of the SAE notification and acknowledgement receipt to the Research Office.

The research team also has the responsibility to report SAEs occurring in a certain period (28 days) after a patient completes the study. Any SAEs reported to the Investigators during this phase must be documented in the patient's medical notes and submitted via an SAE form

11.5 THE TYPE AND DURATION OF THE FOLLOW-UP OF SUBJECTS AFTER AEs

Patients who experience an SAE will be followed up for up to a month after the last observation. AEs be recorded and reported up to one week after the last observation has been made.

11.6 PREGNANCY

The chronic respiratory diseases of interest to this research tend to occur in older people e.g COPD, ILD, bronchiectasis. It is highly unlikely that patients with chronic respiratory disease will be referred to PR. Indeed, there is no record of a pregnant patient being referred to the recruiting site (Harefield PR Unit) since it opened in 2009.

11.7 ANNUAL PROGRESS REPORTS (APRs)

The CI will prepare the APR for the study. It will be reviewed by the RO and sent to the REC by the CI within 30 days of the anniversary date on which the favourable opinion was given by the REC, and annually until the study is declared ended.

11.8 REPORTING URGENT SAFETY MEASURES

The Sponsor and/or the Investigator may take appropriate urgent safety measures in order to protect the subjects of a clinical study against any immediate hazard to their health or safety. If safety measures are taken, REC approval is not required before the measure is taken.

The Investigator will immediately and in any event no later than 3 days from the date the measures are taken, give written notice to the REC and the study Sponsor of the measures taken and the circumstances giving rise to those measures.

In order to prevent any delays in the reporting timelines the Sponsor has delegated this responsibility to the CI. Therefore the CI must report any urgent safety measures to the REC directly, and in parallel to the Sponsor. The REC coordinator will acknowledge receipt of urgent safety measures within 30 days.

12.0 DATA MANAGEMENT AND QUALITY ASSURANCE

12.1 CONFIDENTIALITY

All data will be handled in accordance with the Data Protection Act 1998, NHS Caldecott Principles, The Research Governance Framework for Health and Social Care, 2nd Edition (2005), and the condition of the REC approval.

The CRFs will not bear the subject's name or other personal identifiable data. The subject's initials, date of birth and study identification number, will be used for identification. The CRFs will be stored on-site in Harefield Hospital in filing cabinets that will be locked inside a locked room. Access will only be available to members of the medical/research team. The CRFs will not be transferred off-site.

The multiple deprivation index of each participant will be calculated by inputting the patient's home postcode onto a UK government website ('The English Indices of Deprivation 2015'): http://imd-by-postcode.opendatacommunities.org/ . The postcode is not saved on the website nor is any other identifiable data inputted onto this website. Consent will be sought to use the patient's home postcode to do this.

A web based electronic data capture (EDC) system will be designed, using the InferMed Macro 4 system. The EDC will be created in collaboration with the trial analyst/s and the CI and maintained by the King's Clinical Trials Unit (KCTU) for the duration of the project. It will be hosted on a dedicated secure server within Kings College London. The CI or delegate will request usernames and passwords from the KCTU. Database access will be strictly restricted through user-specific passwords to the authorised research team members. It is a legal requirement that passwords to the EDC are not shared, and that only those authorised to access the system are allowed to do so. If new staff members join the study, a user-specific username and password will be requested via the CI or delegate (e.g Trial Manger) from the KCTU team and a request for access to be revoked will be requested when staff members leave the project. No identifiable data beyond participant initials and date of birth will be entered on the EDC or transferred to the KCTU. No data will be entered onto the EDC system unless a participant has signed a consent form to participate in the trial. Source data will be entered [by recruiting site staff, typically within one week of data collection by authorised staff onto the EDC by going to www.ctu.co.uk and clicking the link to access the MACRO 4 EDC system. A full audit trial of data entry and any subsequent changes to entered data will be automatically date and time stamped, alongside information about the user making the entry/changes within the system. Upon request, KCTU will provide a copy of the final exported dataset to the CI in .csv format and the CI will distribute this to the trial statistician for analysis.

The application to NHS Digital for information on patients' healthcare resource usage requires these identifiers: NHS number, name, date of birth and postcode to be emailed to NHS Digital –

consent will be sought for this in the consent form. This file will be password protected and will only be emailed through secure nhs.net email addresses.

Files e.g. file containing data for NHS Digital will be stored on the network drive at Royal Brompton & Harefield Hospitals. Access to the NHS computers requires user names and passwords. Further access to particular network drives requires permission from senior line managers. Files within the limited access network drive will be password protected.

Any files transferred electronically will be anonymised/encrypted and password protected and emailed through secure nhs.net email addresses.

12.2 DATA COLLECTION TOOL

CRFs will be designed by the CI and the final version will be reviewed and discussed with the study Sponsor. All data will be entered legibly in black ink with a ball-point pen. If the Investigator makes an error, it will be crossed through with a single line in such a way to ensure that the original entry can still be read. The correct entry will then be clearly inserted. The amendment will be initialled and dated by the person making the correction immediately. Overwriting or use of correction fluid will not be permitted.

It is the Investigator's responsibility to ensure the accuracy of all data entered and recorded in the CRFs. The Delegation of Responsibilities Log will identify all study personnel responsible for data collection, entry, handling and managing the database.

12.3 DATA HANDLING AND ANALYSIS

- Data handling: A web based electronic data capture (EDC) system will be designed, using the InferMed Macro 4 system. The EDC will be created in collaboration with the trial analyst/s and the CI and maintained by the King's Clinical Trials Unit (KCTU) for the duration of the project. It will be hosted on a dedicated secure server within KCL.

  The CI or delegate will request usernames and passwords from the KCTU. Database access will be strictly restricted through user-specific passwords to the authorised research team members. It is a legal requirement that passwords to the EDC are not shared, and that only those authorised to access the system are allowed to do so. If new staff members join the study, a user-specific username and password must be requested via the CI or delegate (e.g Trial Manger) from the KCTU team and a request for access to be revoked must be requested when staff members leave the project. Study site staff experiencing issues with system access or functionality should contact the CI or delegate (e.g Trial Manger) in the first instance.

No identifiable data beyond participant initials and date of birth will be entered on the EDC or transferred to the KCTU. No data will be entered onto the EDC system unless a participant has signed a consent form to participate in the trial. Source data will be entered by recruiting site staff, typically within one week of data collection by authorised staff onto the EDC by going to www.ctu.co.uk and clicking the link to access the MACRO 4 EDC system. A full audit trial of data entry and any subsequent changes to entered data will be automatically date and time stamped, alongside information about the user making the entry/changes within the system.

The CI team will undertake appropriate reviews of the entered data, [in consultation with the project analyst]*delete where appropriate for the purpose of data cleaning and will request amendments as required.

At the end of the trial, the site PI will review all the data for each participant if not using this functionality to verify that all the data are complete and correct. At this point, all data can be formally locked for analysis.

- Data analysis: Upon request, KCTU will provide a copy of the final exported dataset to the CI in .csv format and the CI will onward distribute as appropriate

12.4 ARCHIVING ARRANGEMENTS

The study documents (including SMF, CRFs, ICFs) will be kept for a minimum of five years. They will be stored in locked offices within the Royal Brompton and Harefield Hospitals. The CI is responsible for the secure archiving of study documents. The final exported study dataset will also be kept electronically on the RB&H computer network, for a minimum of five years.

The approved repository for longer retention of local materials for studies that involve RB&H patients is Box-It Storage UK. The study documentation will be prepared for archiving by the research team in line with the Research Office Archiving SOP and the transfer will be arranged by the Research Office.

13.0 STATISTICAL DESIGN

13.1 SAMPLE SIZE AND RECRUITMENT

Previous audits of the Harefield PR service have shown that in participants undergoing PR-specialist achieve a mean (SD) change in ISW of 58 (67) metres. The null hypothesis is that the experimental treatment (PR-min) is inferior to the standard treatment (PR-specialist). The alternative hypothesis is that PR-min is not inferior to PR-specialist. The non-inferiority margin will be defined as half the known MCID using the fixed-margin method with a preserved effect of 50% as recommended by previous guidance, including from the United States Food and Drug Administration.[18 19] The MCID of the ISW is 47.5 metres,[20] and therefore 24 metres will be considered the non-inferiority margin. If there is truly no difference between PR-min and PR-specialist, then a minimum of 246 patients (123 in each group) is required to be 80% sure that the lower limit of a one-sided 97.5% CI (or equivalently a 95% two-sided CI) will be above the non-inferiority limit of -24 metres. Based on audit data, we anticipate 32% drop out from PR (12% from assessment to starting PR, and 20% from starting PR to completing PR). Taking into account drop-out, the minimum sample size required for analysis will be 362 patients (181 patients per group).

Recruitment will take place from the Harefield Hospital PR programme which receives 1000 referrals a year. We anticipate 700 eligible patients for recruitment per year. Based on our experience from previous studies, our research recruitment and consent rate is consistently above 65%. Our proposal sample size is 362 patients. Using a conservative estimate of 40% recruitment rate, we anticipate recruitment of 362 patients will take 15-18 months. Due to difficulties delivering the intervention because of COVID-19, the sample size has been increased by 74 participants to 436 (218 per group).

13.2 ENDPOINTS

13.2.1 Primary endpoints

Change in exercise capacity measured by the ISW distance from Visit 1 to Visit 2 according to treatment arm.

13.2.2 Secondary endpoints

- Change in breathlessness measured using the CRQ-D from Visit 1 to Visit 2 and Visit 1 to Visit 3.
- Change in disease-specific health related quality of life measured using the CRQ from Visit 1 to Visit 2 and Visit 1 to Visit 3.
- Change in lower limb muscle strength measured using isometric QMVC from Visit 1 to Visit 2 and Visit 1 to Visit 3.

- Trial process details: number of patients recruited to the trial; proportion of patients that uptake, adhere to and complete PR; reasons for PR non-completion and proportions of patients satisfaction levels on the GROC. This information will be collected in each arm of the study, at the appropriate stage of the trial.
- The cost and cost-effectiveness of the intervention from the perspective of the NHS.

13.3 STATISTICAL ANALYSIS PLAN

Data analysis will be conducted by the Trial Statistician Dr Gao Wei using STATA 14. Data will be analysed on both a per protocol and an intention to treat basis.

A CONSORT flow diagram will be drawn up to describe the number of patients eligible for the study, the number that consent to it as well as the proportion that uptake, adhere to and complete PR. The proportion of patients that attend each visit (visit 1, 2, 3) and complete the study protocol will be included. A full statistical analysis plan will be developed prior to any testing.

Baseline characteristics for each group will be expressed using descriptive statistics (mean (95% CI), or median (25th, 75th centiles). PR completion rates and the number of PR sessions attended will be reported in both groups and compared using a chi square test and independent sample t-test respectively. Baseline characteristics of completers and non-completers will be expressed and compared as for the whole group analysis.

Change in outcomes from visit 1 to 2 will be compared using paired t-tests (or Wilcoxon signed-rank test for non-parametric variables). The proportions of people achieving the MCID of the primary outcome measure (ISW) and secondary outcome measures (CRQ-D, CRQ-T and QMVC) will be compared using chi-squared test. This will be repeated for change in outcomes from visit 1 to 3.

Between-group comparisons will be made using independent sample t-tests (or Mann-Whitney for non-parametric), and 95% CI of primary and secondary outcome measures will be represented graphically in order to determine non-inferiority. A sensitivity analysis will be performed in those in the upper quartile for baseline ISW.

We will compare PR-min with PR-specialist with a view to demonstrating that PR-min is not inferior to PR-specialist in terms of effects on exercise capacity, dyspnoea, health-related quality of life, quadriceps strength.

13.3.1 Primary endpoint analysis

Change in ISW from visit 1 to 2 will be compared using paired t-test (or Wilcoxon signed-rank test if the data are non-parametric). The proportions of people achieving the MCID of the ISW will be compared using chi-squared test.

Between-group comparisons will be made using independent sample t-tests (or Mann-Whitney for non-parametric). A sensitivity analysis will be performed in those in the upper quartile for baseline ISW.

Non-inferiority will be determined using the fixed-margin method as reported by Piaggio[18] in an extension of the CONSORT statement. This method requires a pre-stated margin of non-inferiority for each outcome to be stated (Δ). Δ should be 50% of the MCID to ensure a preserved effect of 50% as recommended by previous guidance.[19] Therefore, Δ for the primary outcome measure of ISW will be pre-defined as 24 metres. The 95% CI of the between group ISW difference will be represented graphically in order to determine non-inferiority.

13.3.2 Secondary endpoint analysis

Change in outcomes from visit 1 to 2 will be compared using paired t-tests (or Wilcoxon signed-rank test for non-parametric data). The proportions of people achieving the MCID of the CRQ-D, CRQ-T and QMVC will be compared using chi-squared test. This will be repeated for change in outcomes from visit 1 to 3.

Between-group comparisons will be made using independent sample t-tests (or Mann-Whitney for non-parametric data).

For the non-inferiority analysis, Δ will be defined as 1.25 points for the CRQ-D and 5 points CRQ-T. The MCID for QMVC has not been established. Based on previous published data of the response of QMVC to PR and neuromuscular electrical stimulation, an improvement of 2.5 kg is considered clinically significant. Therefore Δ will be defined as 1.25 kg for QMVC. The 95% CI of the between group difference of the CRQ-D, CRQ-T and QMVC will be represented graphically in order to determine non-inferiority.

Health-economic analysis: Patient-specific utility profiles will be constructed assuming a straight-line relation between each of the patients' EQ5D5L scores at each follow-up point. The QALYs experienced by each patient from visit 1 to visit 2 and visit 3 will be calculated as the area underneath this profile. Missing data will be explored and appropriate imputation methods will be introduced given characteristic of missingness in EQ5D5L and resource use values. Subsequent analyses of imputed data may include variance correction factors to account for additional variability introduced into parameter values as a result of the imputation process. Cost effectiveness will be calculated as the mean cost difference between trial arms (PR-min and PR-

specialist) divided by the mean difference in outcomes in QALYs to give the incremental cost-effectiveness ratio (ICER).

We will also calculate incremental net monetary benefits (NMBs). Non-parametric methods for calculating confidence intervals around the ICER and incremental NMB based on bootstrapped estimates of the mean cost and QALY differences will be used. The bootstrap replications will also be used to construct a cost-effectiveness acceptability curve, which will show the probability that PR-min is cost-effective in the within-trial period for different values of the cost-effectiveness thresholds (NHS' willingness to pay for an additional QALY) (e.g. £20,000). We will conduct extensive sensitivity analysis to assess the robustness of the results.

13.4 RANDOMISATION

Consenting participants will be randomised at the individual level with a 1:1 allocation, using an independent web-based system provided by the United Kingdom Clinical Research Collaboration registered King's CTU, to receive either "usual care" (PR-specialist) or intervention (PR-min). Randomisation by minimization will ensure that participants will be stratified according to previous completion of PR (yes / no), multiple deprivation index (most deprived quintile of index: yes / no) and frailty status (Short Physical Performance Battery score < 10 / ≥10). A proportion of patients will be entered initially using simple randomisation in order to create a level of initial imbalance and the minimisation algorithm will maintain a level of randomness in order to preserve pre-randomisation allocation concealment. Once randomised, the system will automatically generate a full audit trail of the process and send emails to relevant investigators in a blinded or unblinded format, depending on their role. For each arm, participants will be provided with a choice of three sites to undertake PR.

14.0 COMMITTEES INVOLVED IN THE STUDY

- **Trial Management Group:** Dr. William Man (CI), Ms. Claire Nolan (Project Manager), Dr. Matthew Maddocks (Trial Co-ordinator).

- **Trial Steering Committee:** Dr. William Man (CI), Ms. Claire Nolan (Project Manager), Dr. Matthew Maddocks (Trial Co-ordinator), Ms. Peihan Yu (Blinded Trial Statistician, Mrs. Nannette Spain (PPI representative), Dr. Samantha Kon (Clinical Lead for Hillingdon Integrated Respiratory Service), Dr Mendwas Dzingina (Health Economist).

- **Data Monitoring Ethics Committee:** The members of this committee are not members of the applicants' or sponsors' institution: Professor Michael Steiner (Consultant Respiratory Physician, University Hospitals of Leicester), Dr. Chris Newby (Asthma UK Centre for

Applied Research Lecturer, Pragmatic Clinical Trials Unit, Blizard Institute), Dr Wei Gao (Unblinded Trial Statistician, Cicley Saunders Institute, Kings College London.

15.0 MONITORING AND AUDITING

The requirement for study monitoring or audit will be based on the internal Research Office risk assessment procedure and applicable SOPs. It is the responsibility of the RO to determine the monitoring risk assessment and explain the rationale to the study research team.

Study monitoring and/or audit will be discussed with the CI before arrangements are made to conduct the visit.

16.0 DIRECT ACCESS TO SOURCE DATA

The Investigator(s)/institution(s) will permit study-related monitoring, audits, REC review, and regulatory inspection(s), providing direct access to source data/documents. Study participants are informed of this during the informed consent discussion. Participants will consent to provide access to their medical notes.

17.0 ETHICS AND REGULATORY REQUIREMENTS

The Sponsor will ensure that the study protocol, PIS, ICF, GP letter and submitted supporting documents have been approved by the HRA which includes REC approval if applicable, prior to any patient recruitment taking place. The protocol and all agreed substantial protocol amendments, will be documented and submitted for HRA approval prior to implementation.

Before site(s) can enrol patients into the study confirmation of capacity and capability must be issued by the institution hosting the trial (unless HRA specifically has confirmed in the HRA approval letter that this is not required). It is the responsibility of the CI at each site to ensure that all subsequent amendments gain the necessary approvals by the participating site. This does not affect the individual clinician's responsibility to take immediate action if thought necessary to protect the health and interest of individual patients.

Within 90 days after the end of the study, the CI will ensure that the REC is notified that the study has finished. If the study is terminated prematurely, those reports will be made within 15 days after the end of the study.

The CI will supply a final summary report of the clinical study to the REC and the Sponsor in parallel within one year after the end of the study.

18.0 FINANCE

The National Institute for Health Research is funding this study through a Research for Patient Benefit grant.

19.0 INSURANCE AND INDEMNITY

NHS bodies are liable for clinical negligence and other negligent harm to individuals covered by their duty of care. NHS Institutions employing researchers are liable for negligent harm caused by the design of studies they initiate. The provision of such indemnity for negligent harm should be stated to the participant.

20.0 PUBLICATION POLICY

Data ownership rights will lie with the institution.

21.0 STATEMENT OF COMPLIANCE

The trial will be conducted in compliance with the protocol, Sponsor's SOPs, GCP and the applicable regulatory requirement(s).

The study conduct shall comply with all relevant laws of the EU if directly applicable or of direct effect and all relevant laws and statutes of the UK country in which the study site is located including but not limited to, the Human Rights Act 1998, the Data Protection Act 1998, the Medicines Act 1968, and with all relevant guidance relating to medicines and clinical studies from time to time in force including, but not limited to, the ICH GCP**,** the World Medical Association Declaration of Helsinki entitled 'Ethical Principles for Medical Research Involving Human Subjects' (2008 Version), the NHS Research Governance Framework for Health and Social Care (Version 2, April 2005).

This study will be conducted in compliance with the protocol approved by HRA and according to RGF standards. No deviation from the protocol will be implemented without the prior review and approval of the Sponsor and HRA except where it may be necessary to eliminate an immediate

hazard to a research subject. In such case, the deviation will be reported to the Sponsor and the REC as soon as possible.

22.0 LIST OF PROTOCOL APPENDICES

Appendix 1 Spirometry SOP

Appendix 2 Short Physical Performance Battery SOP

Appendix 3 Incremental Shuttle Walk test SOP

Appendix 4 Chronic Respiratory Questionnaire

Appendix 5 Quadriceps Maximum Voluntary Contraction SOP

Appendix 6 Client Service Receipt Inventory questionnaire

Appendix 7 Euro-Qol – 5 Dimensions – 5 Levels questionnaire

Appendix 8 Global Rate of Change questionnaire

Appendix 1 Spirometry SOP

Spirometry
SOP Reference: HHSpirometry
Version Number: V2.3

Effective Date: 18/11/2016	Date of next review: 18/11/2018
Authors: Melanie Curtis, Dr Jane Canavan, Claire Nolan, Dr William Man	
Approved by: Dr William Man	

Version	Date	Reason for Change
2.0	30/01/2015	Update
2.1	15/07/2015	Clarification
2.2	18/11/2015	Planned revision
2.3	18/11/2016	Planned revision

Table of Contents	Page number
1. Purpose	3
2. Introduction	3
3. Procedure	4
4. Contraindications and precautions	8
5. References	9

PURPOSE

The purpose of spirometry is to assess lung function and to determine lung volumes.

INTRODUCTION

Spirometry is a method of assessing lung function by measuring the volume of air that the patient is able to inhale or exhale and the rate at which that air is forcibly exhaled. The most common spirometric tests require that the patient exhale with as much force as possible after a full, deep inspiration. It is a reliable method of indicating obstructive airways disorders (e.g. COPD, asthma) and restrictive diseases (e.g. interstitial lung disease). It is also the most effective way of determining the severity of airway obstruction (Bellamy et al., 2005).

During each testing session the following tests should be performed, starting with the slow vital capacity manoeuver:

VITAL CAPACITY (VC)
This is (a) the maximal volume of gas which can be expired during a relaxed exhalation, from a full inspiration, or (b) the maximal volume of gas which can be inspired from a maximal expiration. Results should be expressed in litres at body temperature, and ambient pressure saturated with water vapour (BTPS).

FORCED VITAL CAPACITY (FVC)
The maximum volume of air exhaled with maximal effort from a maximal inspiration. Express results in litres at BTPS.

FORCED EXPIRATORY VOLUME IN ONE SECOND (FEV1)
The maximum volume of air exhaled in the first second of a forced exhalation from a position of full inspiration. Express results in litres at BTPS.

THE RATIO OF FEV1 TO FVC (FEV1/FVC)
Obtained by dividing the FEV1 by the FVC, and expressed as a percentage (100 x FEV1/FVC).

PEAK EXPIRATORY FLOW (PEF)
The maximum peak expiratory flow achieved from a maximum forced expiratory manoeuvre (with an open glottis) started without hesitation from a position of maximal inspiration. Express results in litres/minute at BTPS.

ABSOLUTE AND RELATIVE CONTRAINDICATIONS TO PERFORMING SPIROMETRY

ABSOLUTE CONTRAINDICATIONS

- Active Infection.
- Infectious diseases i.e. untreated or active TB.
- Current pneumothorax.
- Unstable aortic, cerebral or abdominal aneurysm.
- Recent surgery (ophthalmic, thoracic, abdominal or neurosurgery).

RELATIVE PRECAUTIONS

- Chest infection in the last 4-6 weeks.
- Undiagnosed chest symptoms e.g. haemoptysis.
- Previous pneumothorax in the last 3 months.
- Recent MI (in the past month).
- Uncontrolled hypertension.
- Pulmonary embolism.
- History of a haemorrhagic event.
- Previous thoracic, abdominal or ophthalmic surgery.

FACTORS THAT MAY INFLUENCE TESTING

- Oral or facial pain, stress incontinence.
- If the person is too unwell to perform spirometry at the time of testing.
- Mental confusion.
- Learning disabilities, communication difficulties.

(Hill & Winter, 2013)

PROCEDURE

TEST LOCATION

Spirometry testing will be performed in a private, temperature controlled room. All necessary equipment will be available in the room. The room will be well lit and in a quiet area. For safety the patient should sit in a chair with arms, without wheels.

There should be access to a sink for hand washing with soap, paper towels and alcohol hand gel.

EQUIPMENT

- Spirometer (EasyOne™ diagnostic, ndd Medical Technologies)
  - input: age, height, weight, ethnicity, gender, smoking status, asthma status
- Box of single-use sealed mouthpieces
- Box of single-use nose-clips
- Box of tissues
- Antibacterial wipes
- Clinical gloves

- Chair with arms and without wheels
- Spare AA batteries
- Approved clinical waste bin
- 3L Calibration syringe and syringe adapter
- Assessment form
- Stadiometer
- Patient weighing scales
- Laptop or computer installed with EasyWare software and connection cable

CALIBRATION AND VERIFICATION
The EasyOne Spirometer and syringe require manufacturer calibration every year, and a verification check 3L syringe with an accuracy of +15ml and which has +/- 3% accuracy should be carried out prior to every testing session or after every 10th patient test. The calibration results should be saved and scrutinised for verification failure.

Note the room temperature for entry into the spirometer. Store the calibration syringe and adaptor with the spirometer to minimise fluctuations in temperature whilst verification checks are completed.

MEDICATION PRIOR TO ASSESSMENT

Community Spirometry Clinics

If the person is having diagnostic spirometry or reversibility testing, they should be advised to:

- Stop short acting bronchodilators 4 hours prior to treatment
- Stop long acting beta 2 agonist for 8 hours prior to treatment
- Stop long acting anticholinergic bronchodilators for 36 hours
- The patient may continue inhaled and oral steroids

Pulmonary Rehabilitation Clinics

If the spirometry is to monitor a pre-existing condition then they should be advised to continue all usual inhaled therapy. Repeat tests on separate occasions should always be performed at the same time of day and ideally by the same operator and using the same equipment.

All patients should be asked to

- Avoid smoking for 24 hours before the test
- Avoid eating a large meal for at least 2 hours before the test
- Avoid alcohol for at least 4 hours before the test
- Avoid vigorous exercise before the test
- Avoid wearing tight clothing that may restrict maximal inspiration and expiration

Patients should bring any pre-existing inhalers with them to the appointment.

REASONS TO RESCHEDULE SPIROMETRY

In some cases, spirometry may be contraindicated by a temporary condition that would affect the validity of the manoeuvre. This may include; a chest infection, severe back pain or recent dental work. Testing should be postponed and re-scheduled when symptoms have resolved.

PREPARING THE PATIENT

- Ensure that the patient is seated, in a chair with arms, in an upright position with the soles of the feet in contact with the ground. This ensures that he / she is in a safe position in the event that syncope or dizziness is experienced during the procedure.
- Explain the purpose of the test, describe the tests and the need to perform two types of tests and that repeated blows will be necessary. Adequate rest will be given between each blow.
- Ask the patient for consent, and document this in the notes.
- Ensure the patient does not lean forward during the test and try to avoid coughing where possible.
- Ensure the patient does not block the spirette with their tongue or teeth and makes a good seal with their lips around the mouthpiece.
- Ask the patient to remove false teeth if they are loose.

MEASURING LUNG FUNCTION

- Wash your hands and wipe the spirometer with an anti-bacterial wipe.
- Attach a clean, disposable, spirette to the spirometer – match the ‘v’ on the mouthpiece to the ‘v’ on the spirometer.
- The assessor or patient will position the nose-clip on the patient’s nose ensuring the nostrils are completely occluded.

VC manoeuver

Instruct the patient to:

- Insert the mouthpiece into his/her mouth, bite gently on the mouthpiece and form a tight lip-seal around the mouthpiece.
- Complete tidal breathing for approximately 2-3 breaths until a steady baseline is achieved.
- Next, the patient should inhale maximally into the spirometer, before exhaling slowly to a full expiration followed by a maximal inspiration and return to tidal breathing.
- Remove the spirometer and nose-clip from the patient and allow him / her to recover.

- This manoeuvre should be performed until three technically acceptable and reproducible results are obtained i.e. more manoeuvres may be required if the patient has a poor technique.
- A maximum of 8 manoeuvres for VC should be performed in one testing session.
- Re-book the patient if reproducibility criteria are not met.
- Document in the notes if the patient has any variations from the above technique e.g. poor technique, coughing etc.
- Save all data in the EasyWare software.

FVC/FEV1 manoeuver

Instruct the patient to:

- Hold the spirometer to the side of his / her mouth (or start tidal breathing on the mouthpiece with a good lip seal if using the tidal flow loop (TFL) setting).
- Breathe in as deeply as possible (full inspiration).
- The patient should immediately insert the mouthpiece into his / her mouth and form a tight lip-seal around the mouthpiece (omit the insertion at this point if using TFL).
- The patient should exhale immediately with maximum forced effort to full expiration.
- The instructor should strongly encourage the patient for the duration of this manoeuvre e.g. keep going, keep going…blow all the way out.
- Remove the spirometer and nosepiece from the patient and allow him / her to recover (or complete the TFL by asking the patient to take another fast maximal inspiration before ending the test).
- This manoeuvre should be performed until three technically acceptable and reproducible results are obtained i.e. more manoeuvres may be required if the patient has a poor technique. A maximum of eight tests can be performed in one testing session.
- Document in the notes if the patient has any variations from the above technique e.g. poor technique, coughing, poor lip-seal, bending forwards etc.
- Save all data in the EasyWare software.

As a minimum, three technically acceptable tests must be performed and must meet ARTP reproducibility criteria. "Acceptable" is defined as a manoeuvre that is free from error. "Reproducible" is defined as being without excessive variability between manoeuvres: the FEV1, FVC, VC from the two best volumes from the three acceptable traces must be within 100mls (0.100L) or 5% of each other (whichever is the greatest). The technically best test should be selected and recorded in the notes. There is currently no definitive guidance regarding PEF reproducibility, but ARTP recommendations are to use two best PEF within 10% of each other.

NB: The EasyOne Spirometer uses the GOLD/Hardie (2003) criteria but the traces are screened manually according to the ARTP reproducibility guidelines as previously described.

Performing post-bronchodilator spirometry in the event of an obstructed flow-volume loop:

- Administer bronchodilator (usually 4 x 100 mcg salbutamol as single doses via a spacer or 2.5mcg via a nebuliser).
- Wait for 15 minutes post-administration and re-test spirometry as above.
- Ensure a post-bronchodilator trace is available for comparison with pre-bronchodilator tests and record the response.

ADDITIONAL SAFETY POINTS

- The assessor should be completely familiar with the test procedures and have practiced them with a partner who is ARTP trained, or with a volunteer under the observation of someone experienced in performing spirometry before attempting it with a patient.

- Clearly explain and demonstrate (without blowing into the spirometer) all procedures prior to testing. Participants should be queried to ensure that they understand the instructions. If a participant is uncomfortable performing a test or if you feel that it is not safe for an individual to continue, the test should not be performed. The assessor should stop the test at any point if the participant appears unduly fatigued.

- The test should be stopped if the patient becomes unwell e.g. dizziness, syncope, chest pain etc. and referred to an appropriate health care provider if required. The rapid access chest pain clinic or the outreach team, or respiratory and cardiology registrars are available at short notice.

This SOP will be revised for accuracy and content in November 2018

REFERENCES

Association for Respiratory Technology and Physiology. www.artp.org.uk/

General Considerations for Lung Function Testing Series ""ATS/ERS Task Force: Standardisation of Lung Function Testing". European Respiratory Journal 2005; 26: 319-338

Bellamy D., Booker R, Connellan S, et al. Spirometry in practice – a practical guide to using spirometry in primary care 2nd edition. BTS COPD consortium 2005.

British Thoracic Society and Association for Respiratory Technology and Physiology. Guidelines for the Measurement of Respiratory Function. Respiratory Medicine 1994; 88; 165-194.

Cooper BG. An Update on Contraindications for Lung Function Testing. Thorax 2011; 66: 714-723.

Fabbri, L. M., & Hurd, S. S.. Global strategy for the diagnosis, management and prevention of COPD: 2003 update. *European Respiratory Journal*, 2003; *22*(1), 1-1.

Hardie, JA, BuistAS, Vollmer WM.et al., Risk of over-diagnosis of COPD in asymptomatic elderly never-smokers. *European Respiratory Journal* 2002; 20 (5): 1117-1122.

Hill S & Winter R. A Guide to Performing Quality Assured Diagnostic Spirometry (2013). http://www.artp.org.uk/en/professional/artpstandards/index.cfm/QADS%20Apr%202020

Vestbo, J., Hurd, S. S., Agusti, A. G., Jones, P. W., Vogelmeier, C., Anzueto, A., & Rodriguez-Roisin, R. Global strategy for the diagnosis, management, and prevention of chronic obstructive pulmonary disease: GOLD executive summary. *American journal of respiratory and critical care medicine*, 2013; *187*(4), 347-365.

Appendix 2 Short Physical Performance Battery SOP

Royal Brompton & Harefield NHS
NHS Foundation Trust

Short Physical Performance Battery
SOP Reference: HHSPPB
Version Number: V2-2017
Effective Date: 02/11/2017<br><br>Review Date: 02/11/2019
Authors: Jane Canavan, Dr William Man
Approved by: Dr William Man

Version	Date	Reason for Change
2	02/11/2017	Review

Table of Contents	Page number
1. Purpose	3
2. Introduction	3
3. Procedure	3-9
4. Additional Safety points	10
5. References	20

PURPOSE

The purpose of the short physical performance battery (SPPB) [1-3] is to evaluate lower extremity function, and provide a marker of disease severity and patient frailty.

INTRODUCTION

Assessment of functional limitations has been used in clinical practice for determining current health status, disease progression and response to medication or pulmonary rehabilitation. In non-disabled older persons poor lower extremity performance, as measured by tests of walking, balance, and chair stands, is associated with poor health status, physiological decrements and inactivity [4-8].

The Short Physical Performance Battery (SPPB) is a simple field test (Appendix 1) of lower limb function [1-3] developed by the National Institute on Aging for the Established Populations for Epidemiologic Studies of the Elderly (EPESE). It comprises assessment of standing balance, usual gait speed and ability to stand from a chair. It only requires a chair, a stopwatch and a four metre flat surface, and takes less than 5 minutes to complete. The SPPB has been well-validated in the elderly population, with associations between SPPB score, hospital admission and mortality reported [1-3], even in those who are highly functioning [1].

PROCEDURE

Clearly explain and demonstrate all procedures prior to testing. Participants should be queried to ensure that they understand the instructions.

All of the tests should be performed in the same order as they are presented in this protocol. Standardised instructions to the participants are shown in bold italic (detailed in the SPPB Protocol and Scoring documentation in Appendix 1) and should be given exactly as they are written.

Part One

BALANCE TESTS

These allow an assessment of the participant's ability to hold three basic standing positions with the eyes open.

Required equipment

Stopwatch, SPPB protocol and score sheet (Appendix 1).

Procedure

The positions are side-by-side stand, semi-tandem, and full tandem stand (heel-to-toe) performed in this order (foot stance detailed in Appendix 1, p10). For each position, the assessor describes and then demonstrates the appropriate stand. Show the participant strategies to help balance (arm out to the side, bent knees, move body, but do not move feet). If the participant is unstable with support, do not try the balance tests and code it on the score sheet as "not attempted" and circle "not attempted, you felt unsafe".

Side-by-side stand:

- The participant assumes the correct foot position (supported as necessary by the assessor).
- Once the participant confirms they are steady, withdraw support and say ***"ready begin"*** and start timing;
- Continue until ten seconds have elapsed, or until the participant moves their feet, or grasps the assessor for support. Watch the patient, not the stopwatch.
- Note the balance duration if they hold it for less than ten seconds to the nearest hundredth of a second. Record on the score sheet (Appendix 1).

Participants who are unable to hold the stand for less than ten seconds do not proceed with the other balance tests and are given a score of zero for this section of the SPPB. Successful participants score one point and progress to the next stand.

Semi-tandem stand:

- The participant is asked to stand with the heel of one foot (either foot) placed to the side of the big toe of the other foot.

- Once the participant confirms they are steady, withdraw support and say ***"ready begin"*** and start timing; continue until ten seconds have elapsed, or until the participant moves their feet, or grasps the assessor for support. Watch the patient, not the stopwatch.

- Participants score one additional point if they hold the semi-tandem position for ten seconds and proceed to the final balance test. Failure to hold the position for ten seconds results in a score of zero and end of the balance tests (Appendix 1).

Tandem stand:

- The participant stands with the heel of one foot (either foot) placed directly in front of the toes of the other foot.

- Once the participant confirms they are steady, withdraw support and say ***"ready begin"*** and start timing; continue until ten seconds have elapsed, or until the participant moves their feet, or grasps the assessor for support. Watch the patient, not the stopwatch.

- Participants holding this position for ten seconds are awarded an additional two points. Those who hold it for 3-9.99 seconds are given one additional point and for less than 3 seconds the participant scores zero (Appendix 1).

Part Two

GAIT SPEED TEST

Required equipment
Tape measure, stopwatch, SPPB protocol and score sheet (Appendix 1).

Course layout

- Identify a hard flat surface to prepare the course
- At a convenient starting, point mark the floor with tape (start line)
- Using the tape measure (laid straight and flat at 90° to the start tape) determine 4m and mark this with 1m of tape (the finish line)

Procedure

Show the patient the walking course and ask them to walk at their usual speed, as if they were walking to go to the shops, walking past the finish line before stopping. Use the standardised instructions in Appendix 1. Demonstrate a walk at normal speed and ask if the participant feels safe to attempt the walk. The participant may use a cane or walking aid during the walk, but if the patient is able to walk a short distance without these, they should be encouraged to do so.

- Stand in a position so that you can observe the foot crossing the finishing line; the best position to maintain is to the side and slightly behind the participant, outside of the patients visual field.

- Ensure the participant's toes are just touching the starting line.

- **Start timing** when the participant begins to move. Do <u>not</u> start the watch when you say ***"begin"***.

- **Stop timing** when the participant's first foot crosses the 4m finish line. If the foot lands on the line but doesn't cross it do not stop timing; anticipate when a foot will fully cross the line and stop timing. Record the time to the nearest hundredth of a second.

- **Repeat the walking test.** Remind the patient to walk at their usual speed and to walk through the line.

Use the shorter of the two times for calculating the score as described (Appendix 1) where if the time is more than 8.70 seconds the participant scores one point; between 6.21-8.70 seconds they score two points; if the time is 4.82-6.20 seconds the participant scores three points, and if the time is less than 4.82 seconds they are awarded four points.

Troubleshooting: If there are problems with the correct starting and stopping of the stopwatch, or the participant expresses concern that they did not reproduce their usual speed accurately the gait speed test should be repeated. If the walk was not

attempted or completed, select a reason from the options on the score sheet and give the participant a score of zero.

Ambulatory Oxygen users

An additional helper is required to carry a small oxygen cylinder or concentrator during the gait speed test. In this case ensure you remain behind the patient and do not set the walking speed. Conversely, supply the patient with extra-long tubing (>6m) connected to wall supplied oxygen or an immovable cylinder.

Part Three

CHAIR STAND TEST

This assesses the participant's ability to rise from a chair (i.e. leg strength) without using their arms.

Required equipment

A straight-backed armless chair with a hard seat, stopwatch, SPPB protocol and score sheet (Appendix 1).

Do not use a folding chair, a soft chair, a deep chair, or a chair on wheels. Place the chair next to the wall. There is no standardised floor to sitting surface height of chair described for this test. Each centre should use the same appropriate chair. As a guide, Harefield Hospital uses a chair with a floor to chair height of 48cm height (measured at the side and centre of the chair from the floor to the top surface of the seat).

Procedure

1. Single sit to stand

- Describe and then demonstrate the sit to stand manoeuvre. Fold your arms across your chest and stand up one time from an armless chair placed against a wall.

- Ask the participant to attempt the sit to stand. Record whether the participant was able to rise from the chair without the use of their arms. If the participant is unsuccessful ask them to repeat the stand using their arms. If the patient is unable to complete the chair stand test with their arms folded, or can only do it with the use of their arms, this will result in a score of zero for this section and the end of the test and do not attempt the five sit to stands. If the patient is successful note this down and proceed to the multiple chair stand test.

Five sit to stands

- Ask the participant to stand up straight as quickly as they can five times without stopping in between. Tell the participant to stand up, sit down and then stand up again, keeping their arms folded across their chest.

- Mention that you'll be timing the test with a stopwatch.

- Perform the demonstration, standing and rising as quickly as possible, emphasising the word quickly.

- **Start timing** when the command to ***"stand"*** is given (this is different to the gait speed test where timing begins only when the participant begins to move).

- Count out loud each time the participant straightens their body after the rise. Count the stand number only after the participant has straightened up; do not pace the test with your counting and do not encourage the participant during the test.

- **Stop timing** when the participant stands straight up at the end of the fifth rise.

Troubleshooting: If the participant does not attempt the test, or is unable to complete the test, note the reason on the score sheet. Do not record the time and score the patient zero for this part of the test. The test should be stopped if the participant becomes unduly tired during the repeated chair stands, uses their arms to rise at any time, or if the participant has not completed the five chair stands after one minute. The test may be stopped by the assessor if there are concerns about the participant's safety. If the participant stops before completing the five rises, ask if they can continue. If the participant says yes, continue timing; if no, stop the test and record reason on the score sheet and give the participant a score of zero.

Scoring of the chair stand test is based on established categories of completion times shown to divide the older population into four equal groups (Appendix 1). Participants completing the test between 16.70 and 60 seconds score one point. Completion times falling into the range 13.70-16.69 seconds score two points, while those in the range 11.20-13.69 seconds score three points. If participants finish the five rises in less than 11.20 seconds they receive four points.

Overall scoring for the complete SPPB

- Add together the score from the Balance test (maximum 4 points), Gait Speed test (maximum 4 points) and Sit-to-Stand (maximum 4 points) to get an overall score. The summary score ranges from worst performance (0 points), to best performance (12 points).

ADDITIONAL SAFETY POINTS

The assessor should be completely familiar with the test procedures and have practiced them with a partner who is in training or trained, or with a volunteer under the observation of someone experienced in administering the battery before attempting to administer the test battery to a patient or research subject.

Clearly explain and demonstrate all procedures prior to testing. Participants should be queried to ensure that they understand the instructions. If a participant is uncomfortable performing a test or if you feel that it is not safe for an individual to continue, the test should not be performed. The assessor should stop the tests at any point if the participant appears unduly fatigued.

BALANCE TESTS

If required, the assessor may stabilise the participant by lightly holding his/her arm, or allow the participant to lean against them until their feet are in position. If the participant is not steady, even with support, do not continue with the balance tests. Ensure the participant is stable and feet are in the correct position before releasing him/her. The assessor should stand close enough so that it is possible to seize the patient's arm if they begin to falter, but not so close that the patient's balance is hindered if they use their arms to maintain balance.

GAIT SPEED TEST

Ensure that the course is clear of obstructions before giving the command to walk. The assessor should stay behind and slightly to one side of the participant, outside of his/her visual field, but close enough to support the participant if they begin to fall. A cane or walker may be used during the walk, but if participants can walk without the devices they should be encouraged to do so as this gives a more accurate assessment of functional limitations.

CHAIR STAND TEST

The assessor should ensure that the chair is stable against a wall for example, and that they are close enough to provide support if the patient requires it, but not too close so as to impede movement.

Appendix 3.1

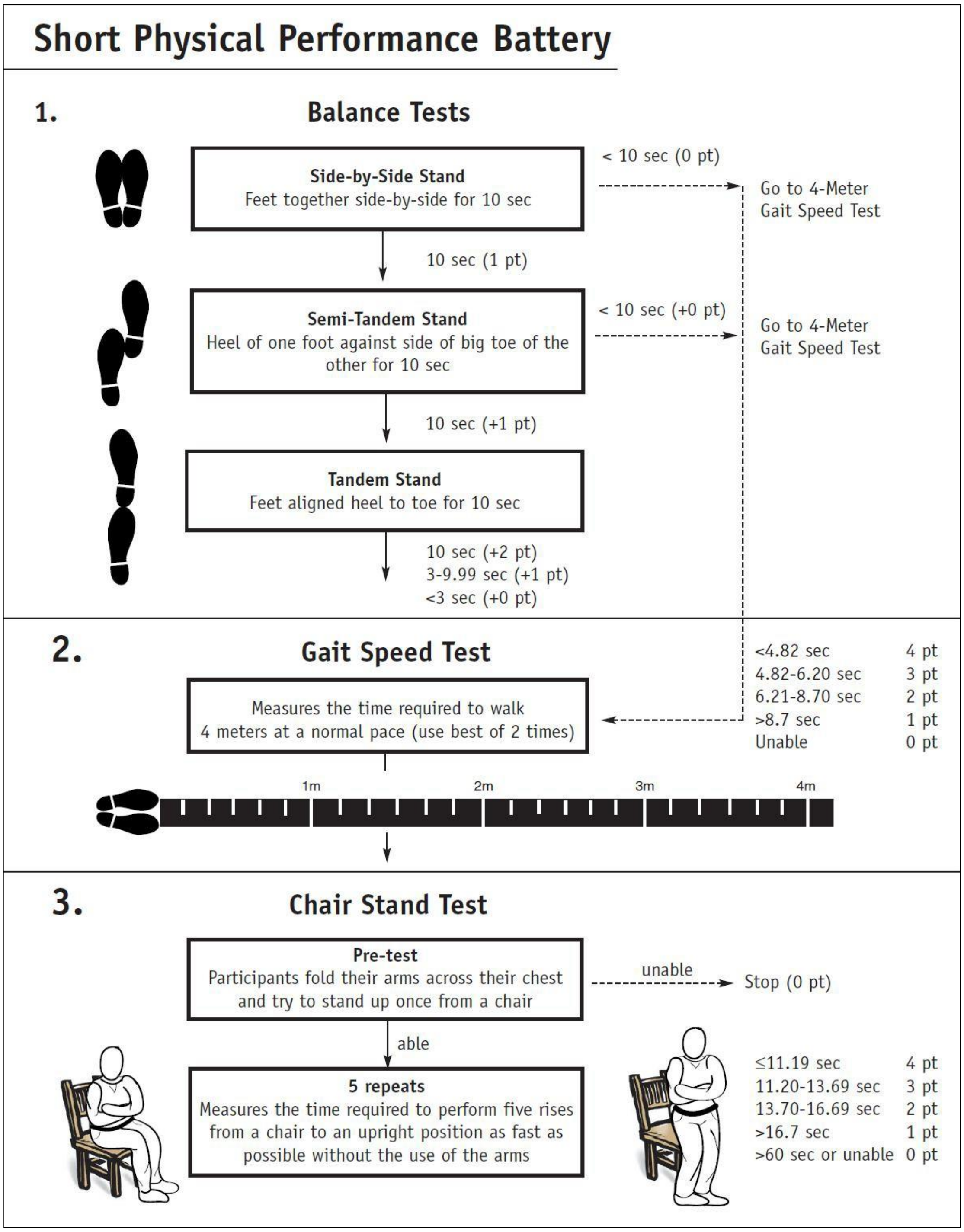
Short Physical Performance Battery
1.
Balance Tests
Side-by-Side Stand
Feet together side-by-side for 10 sec
< 10 sec (0 pt)
Go to 4-Meter Gait Speed Test
10 sec (1 pt)
Semi-Tandem Stand
Heel of one foot against side of big toe of the other for 10 sec
< 10 sec (+0 pt)
Go to 4-Meter Gait Speed Test
10 sec (+1 pt)
Tandem Stand
Feet aligned heel to toe for 10 sec
10 sec (+2 pt)
3-9.99 sec (+1 pt)
<3 sec (+0 pt)
2.
Gait Speed Test
Measures the time required to walk 4 meters at a normal pace (use best of 2 times)
<4.82 sec 4 pt
4.82-6.20 sec 3 pt
6.21-8.70 sec 2 pt
>8.7 sec 1 pt
Unable 0 pt
1m
2m
3m
4m
3.
Chair Stand Test
Pre-test
Participants fold their arms across their chest and try to stand up once from a chair
unable
Stop (0 pt)
able
5 repeats
Measures the time required to perform five rises from a chair to an upright position as fast as possible without the use of the arms
≤11.19 sec 4 pt
11.20-13.69 sec 3 pt
13.70-16.69 sec 2 pt
>16.7 sec 1 pt
>60 sec or unable 0 pt

Study ID ____________________ Date ________________ Tester Initials __________________

SHORT PHYSICAL PERFORMANCE BATTERY PROTOCOL AND SCORE SHEET

All of the tests should be performed in the same order as they are presented in this protocol. Instructions to the participants are shown in bold italic and should be given exactly as they are written in this script.

1. BALANCE TESTS
The participant must be able to stand unassisted without the use of a cane or walker. You may help the participant to get up.

> ***Now let's begin the evaluation. I would now like you to try to move your body in different movements. I will first describe and show each movement to you. Then I'd like you to try to do it. If you cannot do a particular movement, or if you feel it would be unsafe to try to do it, tell me and we'll move on to the next one. Let me emphasize that I do not want you to try to do any exercise that you feel might be unsafe.***
>
> ***Do you have any questions before we begin?***

A. Side-by-Side Stand

1. ***Now I will show you the first movement.***
2. (Demonstrate) ***I want you to try to stand with your feet together, side-by-side, for about 10 seconds.***
3. ***You may use your arms, bend your knees, or move your body to maintain your balance, but try not to move your feet. Try to hold this position until I tell you to stop.***
4. Stand next to the participant to help him/her into the side-by-side position.
5. Supply just enough support to the participant's arm to prevent loss of balance.
6. When the participant has his/her feet together, ask ***"Are you ready?"***
7. Then let go and begin timing as you say, ***"Ready, begin."***
8. Stop the stopwatch and say ***"Stop"*** after 10 seconds or when the participant steps out of position or grabs your arm.
9. If participant is unable to hold the position for 10 seconds, record result and go to the gait speed test.

Study ID ____________________ Date ________________ Tester Initials ________________

B. Semi-Tandem Stand

1. ***Now I will show you the second movement.***

2. (Demonstrate) ***Now I want you to try to stand with the side of the heel of one foot touching the big toe of the other foot for about 10 seconds. You may put either foot in front, whichever is more comfortable for you.***

3. ***You may use your arms, bend your knees, or move your body to maintain your balance, but try not to move your feet. Try to hold this position until I tell you to stop.***

4. Stand next to the participant to help him/her into the semi-tandem position

5. Supply just enough support to the participant's arm to prevent loss of balance.

6. When the participant has his/her feet together, ask ***"Are you ready?"***

7. Then let go and begin timing as you say ***"Ready, begin."***

8. Stop the stopwatch and say ***"Stop"*** after 10 seconds or when the participant steps out of position or grabs your arm.

9. If participant is unable to hold the position for 10 seconds, record result and go to the gait speed test.

C. Tandem Stand

1. ***Now I will show you the third movement.***

2. (Demonstrate) ***Now I want you to try to stand with the heel of one foot in front of and touching the toes of the other foot for about 10 seconds. You may put either foot in front, whichever is more comfortable for you.***

3. ***You may use your arms, bend your knees, or move your body to maintain your balance, but try not to move your feet. Try to hold this position until I tell you to stop.***

4. Stand next to the participant to help him/her into the tandem position.

5. Supply just enough support to the participant's arm to prevent loss of balance.

6. When the participant has his/her feet together, ask ***"Are you ready?"***

7. Then let go and begin timing as you say, ***"Ready, begin."***

8. Stop the stopwatch and say ***"Stop"*** after 10 seconds or when the participant steps out of position or grabs your arm.

Study ID ____________ Date ____________ Tester Initials ____________

SCORING:

A. Side-by-side-stand

Held for 10 sec ❒ 1 point
Not held for 10 sec ❒ 0 points
Not attempted ❒ 0 points
If 0 points, end Balance Tests

Number of seconds held if less than 10 sec: __.__ __sec

If participant did not attempt test or failed, circle why:	
Tried but unable	1
Participant could not hold position unassisted	2
Not attempted, you felt unsafe	3
Not attempted, participant felt unsafe	4
Participant unable to understand instructions	5
Other (specify) ____________	6
Participant refused	7

B. Semi-Tandem Stand

Held for 10 sec ❒ 1 point
Not held for 10 sec ❒ 0 points
Not attempted ❒ 0 points *(circle reason above)*
If 0 points, end Balance Tests

Number of seconds held if less than 10 sec: __.__ __sec

C. Tandem Stand

Held for 10 sec ❒ 2 points
Held for 3 to 9.99 sec ❒ 1 point
Held for < than 3 sec ❒ 0 points
Not attempted ❒ 0 points (circle reason above)

Number of seconds held if less than 10 sec: __.__ __sec

D. Total Balance Tests score__________(sum points)

Comments: ____________

Study ID ____________________ Date ________________ Tester Initials ________________

2. GAIT SPEED TEST

Now I am going to observe how you normally walk. If you use a cane or other walking aid and you feel you need it to walk a short distance, then you may use it.

A. First Gait Speed Test

1. ***This is our walking course. I want you to walk to the other end of the course at your usual speed, just as if you were walking down the street to go to the store.***
2. Demonstrate the walk for the participant.
3. ***Walk all the way past the other end of the tape before you stop. I will walk with you. Do you feel this would be safe?***
4. Have the participant stand with both feet touching the starting line.
5. ***When I want you to start, I will say: "Ready, begin."*** When the participant acknowledges this instruction say: ***"Ready, begin."***
6. Press the start/stop button to start the stopwatch as the participant begins walking.
7. Walk behind and to the side of the participant.
8. Stop timing when one of the participant's feet is completely across the end line.

B. Second Gait Speed Test

1. ***Now I want you to repeat the walk. Remember to walk at your usual pace, and go all the way past the other end of the course.***
2. Have the participant stand with both feet touching the starting line.
3. ***When I want you to start, I will say: "Ready, begin."*** When the participant acknowledges this instruction say: ***"Ready, begin."***
4. Press the start/stop button to start the stopwatch as the participant begins walking.
5. Walk behind and to the side of the participant.
6. Stop timing when one of the participant's feet is completely across the end line.

Study ID ____________________ Date ________________ Tester Initials __________________

GAIT SPEED TEST SCORING:

Length of walk test course: Four meters ❐ Three meters ❐

A. Time for First Gait Speed Test (sec)

1. Time for 3 or 4 meters __ __.__ __sec
2. If participant did not attempt test or failed, circle why:

Tried but unable	1
Participant could not walk unassisted	2
Not attempted, you felt unsafe	3
Not attempted, participant felt unsafe	4
Participant unable to understand instructions	5
Other (Specify) ____________________	6
Participant refused	7

Complete score sheet and go to chair stand test

3. Aids for first walk...............None ❐ Cane ❐ Other ❐

Comments:____

____

____

B. Time for Second Gait Speed Test (sec)

1. Time for 3 or 4 meters __ __.__ __ sec
2. If participant did not attempt test or failed, circle why:

Tried but unable	1
Participant could not walk unassisted	2
Not attempted, you felt unsafe	3
Not attempted, participant felt unsafe	4
Participant unable to understand instructions	5
Other (Specify)	6
Participant refused	7

3. Aids for second walk............ None ❐ Cane ❐ Other ❐

What is the time for the faster of the two walks?
Record the shorter of the two times __ __.__ __ sec
[If only 1 walk done, record that time] __ __.__ __ sec

If the participant was unable to do the walk: ❐ **0 points**

**For 4-Meter Walk:**		**For 3-Meter Walk:**	
If time is more than 8.70 sec:	❐ **1 point**	If time is more than 6.52 sec:	❐ **1 point**
If time is 6.21 to 8.70 sec:	❐ **2 points**	If time is 4.66 to 6.52 sec:	❐ **2 points**
If time is 4.82 to 6.20 sec:	❐ **3 points**	If time is 3.62 to 4.65 sec:	❐ **3 points**
If time is less than 4.82 sec:	❐ **4 points**	If time is less than 3.62 sec:	❐ **4 points**

Study ID ____________________ Date ________________ Tester Initials ________________

3. CHAIR STAND TEST

Single Chair Stand

1. ***Let's do the last movement test. Do you think it would be safe for you to try to stand up from a chair without using your arms?***
2. ***The next test measures the strength in your legs.***
3. (Demonstrate and explain the procedure.) ***First, fold your arms across your chest and sit so that your feet are on the floor; then stand up keeping your arms folded across your chest.***
4. ***Please stand up keeping your arms folded across your chest.*** (Record result).
5. If participant cannot rise without using arms, say ***"Okay, try to stand up using your arms."*** This is the end of their test. Record result and go to the scoring page.

Repeated Chair Stands

1. ***Do you think it would be safe for you to try to stand up from a chair five times without using your arms?***
2. (Demonstrate and explain the procedure): ***Please stand up straight as QUICKLY as you can five times, without stopping in between. After standing up each time, sit down and then stand up again. Keep your arms folded across your chest. I'll be timing you with a stopwatch.***
3. When the participant is properly seated, say: ***"Ready? Stand"*** and begin timing.
4. Count out loud as the participant arises each time, up to five times.
5. Stop if participant becomes tired or short of breath during repeated chair stands.
6. Stop the stopwatch when he/she has straightened up completely for the fifth time.
7. Also stop:
   - If participant uses his/her arms
   - After 1 minute, if participant has not completed rises
   - At your discretion, if concerned for participant's safety
8. If the participant stops and appears to be fatigued before completing the five stands, confirm this by asking ***"Can you continue?"***
9. If participant says "Yes," continue timing. If participant says "No," stop and reset the stopwatch.

Study ID ____________ Date ____________ Tester Initials ____________

SCORING
Single Chair Stand Test

		YES	NO
A.	Safe to stand without help	❒	❒

B. Results:

Participant stood without using arms	❒	→ Go to Repeated Chair Stand Test
Participant used arms to stand	❒	→ End test; score as 0 points
Test not completed	❒	→ End test; score as 0 points

C. If participant did not attempt test or failed, circle why:

Tried but unable	1
Participant could not stand unassisted	2
Not attempted, you felt unsafe	3
Not attempted, participant felt unsafe	4
Participant unable to understand instructions	5
Other (Specify) ____________	6
Participant refused	7

Repeated Chair Stand Test

		YES	NO
A.	Safe to stand five times	❒	❒

B. If five stands done successfully, record time in seconds.

Time to complete five stands __ __.__ __ sec

C. If participant did not attempt test or failed, circle why:

Tried but unable	1
Participant could not stand unassisted	2
Not attempted, you felt unsafe	3
Not attempted, participant felt unsafe	4
Participant unable to understand instructions	5
Other (Specify)	6
Participant refused	7

Scoring the Repeated Chair Test

Participant unable to complete 5 chair stands or completes stands in >60 sec:	❒ 0 points
If chair stand time is 16.70 sec or more:	❒ 1 points
If chair stand time is 13.70 to 16.69 sec:	❒ 2 points
If chair stand time is 11.20 to 13.69 sec:	❒ 3 points
If chair stand time is 11.19 sec or less:	❒ 4 points

Study ID ____________________ Date ________________ Tester Initials ________________

Scoring for Complete Short Physical Performance Battery

Test Scores
Total Balance Test score _____ **points**
Gait Speed Test score _____ **points**
Chair Stand Test score _____ **points**

Total Score _____ **points (sum of points above)**

REFERENCES

1. Guralnik, J.M., et al., *Lower-extremity function in persons over the age of 70 years as a predictor of subsequent disability.* N Engl J Med, 1995. **332**(9): p. 556-61.
2. Guralnik, J.M., et al., *A short physical performance battery assessing lower extremity function: association with self-reported disability and prediction of mortality and nursing home admission.* J Gerontol, 1994. **49**(2): p. M85-94.
3. Guralnik, J.M., et al., *Lower extremity function and subsequent disability: consistency across studies, predictive models, and value of gait speed alone compared with the short physical performance battery.* J Gerontol A Biol Sci Med Sci, 2000. **55**(4): p. M221-31.
4. Ferrucci, L., et al., *Characteristics of nondisabled older persons who perform poorly in objective tests of lower extremity function.* J Am Geriatr Soc, 2000. **48**(9): p. 1102-10.
5. Seeman, T.E., et al., *Predicting changes in physical performance in a high- functioning elderly cohort: MacArthur studies of successful aging.* J Gerontol, 1994. **49**(3): p. M97-108.
6. Rantanen, T., P. Era, and E. Heikkinen, *Maximal isometric knee extension strength and stair-mounting ability in 75- and 80-year-old men and women.* Scand J Rehabil Med, 1996. **28**(2): p. 89-93.
7. Seeman, T.E., et al., *Behavioral and psychosocial predictors of physical performance: MacArthur studies of successful aging.* J Gerontol A Biol Sci Med Sci, 1995. **50**(4): p. M177-83.
8. Penninx, B.W., et al., *Lower extremity performance in nondisabled older persons as a predictor of subsequent hospitalization.* J Gerontol A Biol Sci Med Sci, 2000. **55**(11): p. M691-7.

Appendix 3 Incremental Shuttle Walk test SOP

Royal Brompton & Harefield NHS
NHS Foundation Trust

**Incremental Shuttle Walk Test**
SOP Reference: HHISW
Version Number: V3 - 2017
Effective Date: 07/11/2017<br><br>Review Date: 07/11/2019
Authors: Claire Nolan, Dr. William Man, Ruth Barker
Approved by: Dr. William Man

Version	Date	Reason for Change
3	07/11/2017	Update and review

Table of Contents	Page number
1. Purpose	3
2. Introduction	3
3. Procedure	3
4. Additional Safety points	5
6. Appendices	7
5. References	11

PURPOSE

The purpose of the incremental shuttle walk test (ISW) is to assess maximal exercise capacity, cardiorespiratory response to exercise and prescribe walking speed.

INTRODUCTION

The ISW is a standardised externally-paced, progressive, incremental field walking test for patients with chronic airways disease comprising 12 levels and 102 shuttles. Patients walk around a 10m shuttle course marked by cones in time with a series of bleeps played from a CD.

The ISW has been shown to be a valid and reproducible measure of exercise capacity in subjects with COPD (Singh et al., 1992). It produces a higher peak heart-rate and Borg Dyspnoea score as well as a more graded cardiorespiratory response to exercise compared to the 6 minute walk test (6MWD) (Singh et al., 1992).

It is used as an outcome measure of exercise capacity in pulmonary rehabilitation. An improvement of 47.5 metres following PR indicates that patients with COPD are 'slightly better' and an improvement of 78.7 metres represents 'better' (Singh et al., 2008).

The ISW is undertaken as described in the ERS/ATS technical standard for field walking tests in chronic respiratory disease (Holland et al, 2014).

PROCEDURE

Required equipment

- Two cones
- Two chairs
- Stopwatch
- Pulse-oximeter
- Sphygmomanometer
- Borg Dyspnoea scale
- CD player with ISW CD
- Typed explanation of the ISW instructions (Appendix 1)
- PR assessment sheet / research scoring sheet
- Clipboard and pen
- Access to oxygen and nasal cannula / face mask (if the patient is going to perform the test using oxygen)

Protocol

Before the test

- If this is the patient's initial assessment, document the past-medical history
- Identify and record if the patient will do the test using a walking aid.

- Measure blood pressure, heart-rate, resting oxygen saturations and Borg Dyspnoea level **(do not perform the test if systolic blood pressure is ≥180mmHg, diastolic blood pressure is ≥100mmHg, resting heart rate is ≥120bpm).**
- If at the initial assessment the patients resting oxygen saturations are <92% the test should be performed using oxygen (Appendix 1). The patient should not carry the oxygen cylinder. Assessors should carry the patient's oxygen during the test. They should ensure that they walk behind the subject to avoid setting the walking pace.
- If performing the end of course assessment, the patient should use oxygen only if the initial assessment was performed using oxygen or if a senior physiotherapist deems it unsafe to assess the patient without oxygen. The patient should not carry the oxygen cylinder. Assessors should carry the patient's oxygen during the test. They should ensure that they walk behind the subject to avoid setting the walking pace.
- Set-up the course: two cones placed 9 metres apart on flat, straight flooring with a chair 1 metre behind each cone (Figure 1).
- Show the patient the course and play the standardised instructions on the CD player or read the typed standardised instructions to the patient (Appendix 2).
- Ask the patient if he / she understands the instructions and answer any posed questions.
- Before the test starts remind the patient that 'this is a maximal test, by the end of the test you should walk or run as fast as you can.'

Figure 1: ISW course layout

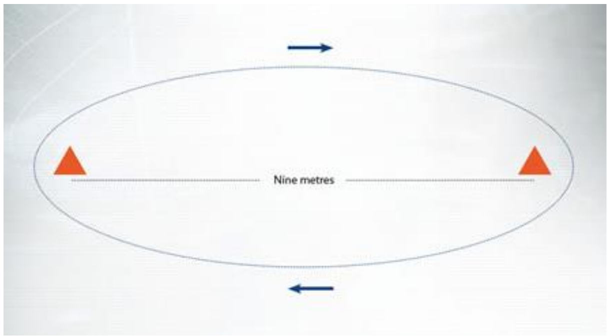

<u>During the test</u>

- Walk with the patient for the first level (3 shuttles) to ensure correct pacing.
- If the patient is not using oxygen, check the patient's oxygen saturations before the end of the 3rd shuttle. If they drop below 88% the test must be stopped and recommenced using oxygen. (Appendix 2). The patient should not carry the oxygen cylinder. Assessors should carry the patient's oxygen during the test. They should ensure that they walk behind the subject to avoid setting the walking pace.
- The standardised instructions should be administered as appropriate and the patient should not be encouraged (Appendix 3). It is OK to advise the patient to slow his / her walking speed to ensure a continuous walk.
- At each new level (triple bleep) instruct the patient to '*increase your speed now.'*
- Record each completed shuttle and monitor the patient's heart-rate and oxygen saturations.
- If the patient is less than 0.5m from the cone do not say anything.

- If the patient is greater than 0.5m from the cone advise the patient that '*You need to increase your speed to keep up with the test'.* Only record this shuttle if the patient is less than 0.5m from the cone on the next shuttle.
- The test can be terminated for a number of reasons:
  - Objective reasons:
    - The patient is greater than 0.5m from the cone two shuttles in a row (i.e. you have given the patient a chance to increase his / her speed and he / she is not within 0.5m of the cone).
    - Heart rate > 85% predicted maximum heart rate.
    - Sp02 < 80% (A senior physiotherapist may continue the test if the patient appears clinically well. Physiotherapy assistants should seek advice from a senior physiotherapist if this situation arises).
    - Chest pain, light-headedness, dizziness, unstable gait pattern, evolving mental confusion, any other clinically warranted reason.

  - Subjective reasons:
    - The patient is too short of breath to continue.
    - The patient's legs are too tired or sore to continue.
    - Any limiting pain.

<u>After the test</u>

- Sit the patient on a chair (if patient is able to walk to one of the chairs behind the cones or if the patient is unable to walk bring a chair to the patient).
- Immediately record heart rate, oxygen saturations and Borg Dyspnoea level.
- Immediately begin timing recovery, recording the individual recovery time for heart rate, oxygen saturations and Borg Dyspnoea level, only stop timing when the observations have returned to the pre-test levels or when 6 minutes has elapsed.
- Record completed shuttles only i.e. only those where the patient was less than 0.5m from the cone.
- Record why the test ended
  - *"What do you think stopped you from keeping up with the beeps?"*

REPEAT TEST

A learning effect is associated with the ISW. The test should be repeated after 30 minutes rest, and <u>the best distance of the two tests recorded</u>.

ADDITIONAL SAFETY POINTS

Clearly explain and demonstrate all procedures prior to testing. Participants should be queried to ensure that they understand the instructions. If a participant is uncomfortable performing a test or if you feel that it is not safe for an individual to continue, the test should not be performed.

1. Testing should be performed in a location where a rapid, appropriate response to an emergency is possible. The appropriate location of the crash trolley should be determined by the assessor undertaking the test.
2. Oxygen must be available. If applicable, the patient's sublingual nitroglycerine and or salbutamol, must also be accessible. A telephone or other means should be in place to enable a call for help.
3. The technician should be certified in cardiopulmonary resuscitation with a minimum of Basic Life Support by Resuscitation Council (UK)–approved cardiopulmonary resuscitation course. Adult Intermediate Life Support Training is desirable. A certified individual should be readily available to respond if needed.
4. The assessor should be completely familiar with the ISW procedures and have practiced them with a partner who has been trained by someone experienced in administering the ISW before attempting to administer the test to a patient.

APPENDIX 1

ISW OXYGEN PROTOCOL

RESTING

- SATS ON ROOM AIR ABOVE **92%** - **OK** !
- SATS **92%** OR LOWER AND NOT ON HOME O_2 – REFER FOR LTOT AX

INITIAL ISWT

TEST ON SUPPLEMENTARY OXYGEN IF:

- PATIENT KNOWN TO BE ON HOME OR AMBULATORY O_2
- PATIENT RESTING SATS ON ROOM AIR LESS THAN 92%

IF PATIENT DESATURATES BELOW **88%** WITHIN FIRST THREE SHUTTLES:

- STOP THE TEST AND BEGIN TEST AGAIN ON O_2

IF THE PATIENT DESATURATES AFTER THE FIRST THREE LENGTHS, CONTINUE THE WALK TEST UNTIL SATS FALL BELOW **80%**.

- IF BELOW **80%** AT ANY TIME, CEASE THE TEST
    - A senior physiotherapist may continue the test if the patient appears clinically well. Physiotherapy assistants should seek advice from a senior physiotherapist if this situation arises).
- CONSIDER REFERRAL FOR AMBULATORY O_2 AX

COURSE

EXERCISE ON O_2 IN CLASS

- IF DESATURATION BELOW **88%** AT ANY TIME DURING INITIAL ASSESSMENT OR DURING COURSE
- KNOWN TO BE ON HOME OR AMBULATORY O_2
- RESTING SATS ON ROOM AIR LESS THAN 92%

END OF COURSE ASSESSMENT

- IN GENERAL, END OF COURSE SHOULD BE DONE IN THE SAME CONDITIONS AS INITIAL ASSESSMENT UNLESS IT IS DEEMED UNSAFE TO DO SO BY A SENIOR PHYSIOTHERAPIST

PATIENT DECLINING OXYGEN

- EXPLAIN BENEFITS

- IF STILL DECLINING, REFER TO WILL & COMPLETE THE ‘DECLINE OXYGEN LETTER (APPENDIX 4) AND FILE IN THE HOSPITAL NOTES.

APPENDIX 2

VERBAL INSTRUCTIONS FOR THE INCREMENTAL SHUTTLE WALK TEST

The object of the progressive shuttle walking test is to walk for as long as possible there and back along the 10 metre course keeping to the speed indicated by the beeps on the CD. You will hear these beeps at regular intervals. You should walk at a steady pace aiming to turn around the cone at one end of the course when you hear the first beep and at the other end when you hear the next.

At first your walking speed will be very slow but you will need to speed up at the end of each minute. Your aim should be to follow the set rhythm for as long as you can. Each single beep signals the end of a shuttle and each triple beep signals an increase in walking speed. You should stop walking only when you become too breathless to maintain the required speed or can no longer keep up with the set pace.

The test is maximal and progressive, in other words, it is easier at the start and harder at the end. Your walking speed for the first minute is very slow and you have 20 seconds to complete each 10 metre shuttle, so don't go too fast.

Level one starts with a triple beep after the 4 second count down.

Note to the assessor: before you start the ISW remind patient that *'this is a maximal test, by the end of the test you should walk or run as fast as you can'.*

APPENDIX 3

Standardised instructions during the ISW

Follow the instructions on the CD, and use the following standard prompts:

- Each time the triple beep sounds advise the patient to: *"Increase your speed now."*

- Use the following prompt if the patient is more than 0.5 m away from the cone when the beep sounds: *"You need to increase your speed to keep up with the test."*

APPENDIX 4

Decline Oxygen Letter

A lifetime of specialist care

Royal Brompton & Harefield NHS
NHS Foundation Trust

Dear

It has been noted that during your pulmonary rehab assessment / classes your oxygen levels drop significantly when exercising. In order to reduce any potential problems and side effects we have offered oxygen therapy to use during your exercise classes. We have discussed the possible side effects and you are aware of any risks this may result in.

Please sign below to indicate that you have declined the offer of oxygen therapy during the pulmonary rehabilitation programme and you accept the risks.

Signed……………………………… Date………………………………..

Patient:

Signed………………………………… Date…………………………………….

Staff:

REFERENCES

- Singh SJ et al,(1992) *"Development of a shuttle walking test of disability in patients with chronic airways obstruction"* Thorax 47 (1019 – 1024)

- S Singh et al, (2008) "*Minimum clinically important improvement for the incremental shuttle walking test"* Thorax 63 (775 – 777)

- Holland A et al., *"An official European Respiratory Society/American Thoracic Society technical standard: field walking tests in chronic respiratory disease".* European Respiratory Journal 44.6 (2014): 1428-1446

Appendix 4 Chronic Respiratory Questionnaire

CHRONIC RESPIRATORY QUESTIONNAIRE

SELF REPORTED (ASSESSMENT)

This questionnaire is designed to find out how you have been feeling during the last two weeks. You will be asked how short of breath you have been, how tired you have been feeling and how your mood has been.

NAME____

DATE____

We would like you to think of ways in which your shortness of breath limits your life. We are particularly interested in activities, which you still do, but which are limited by your shortness of breath. Listed below are some activities, which can make people with lung problems feel short of breath.

If you have felt **short of breath** doing any of the **activities** listed below **during the last two weeks** then please tick each relevant activity. If you have **not** done the activity during the last two weeks or it does **not** make you short of breath then leave it blank.

1. BEING ANGRY OR UPSET ☐
2. HAVING A BATH OR SHOWER ☐
3. BENDING ☐
4. CARRYING - SUCH AS GROCERIES ☐
5. DRESSING ☐
6. EATING ☐
7. GOING FOR A WALK ☐
8. DOING YOUR HOUSEWORK ☐
9. HURRYING ☐
10. MAKING YOUR BED ☐
11. MOPPING OR SCRUBBING A FLOOR ☐
12. MOVING FURNITURE ☐
13. PLAYING WITH CHILDREN/GRANDCHILDREN ☐
14. PLAYING SPORTS ☐
15. REACHING OVER YOUR HEAD ☐
16. RUNNING - SUCH AS FOR A BUS ☐
17. SHOPPING ☐
18. WHILE TRYING TO SLEEP ☐
19. TALKING ☐
20. VACUUMING ☐
21. WALKING AROUND YOUR OWN HOME ☐
22. WALKING UPHILL ☐
23. WALKING UP STAIRS ☐
24. WALKING WITH OTHERS ON LEVEL GROUND ☐
25. PREPARING MEALS ☐

Please list **any other activities** that you have done during the last two weeks, which have made you feel short of breath. These should be activities, which you do frequently and which are important in your day-to-day life.

Other Activities:
1.
2.
3.

HOW SHORT OF BREATH HAVE YOU BEEN DURING THE LAST TWO WEEKS WHILE PERFORMING THESE ACTIVITIES?

We would now like you to identify the most important activities in which you have been limited by your shortness of breath in the last two weeks. Using the list you have made on the previous page, write down the five most important activities that have made you short of breath on the lines below. We would then like you to tell us how short of breath you have been while performing each activity by ticking the box which best describes how you feel.

THE ACTIVITIES ARE:

	Extremely short of breath	Very short of breath	Quite short of breath	Moderate shortness of breath	Some shortness of breath	A little shortness of breath	Not at al short of breath
**1**							
**2**							
**3**							
**4**							
**5**							

6. In general, how much of the time during the last 2 weeks have you felt frustrated or impatient?
Please indicate how often during the last 2 weeks you have felt frustrated or impatient by ticking one of the following options from the list below.
1. ALL OF THE TIME ☐
2. MOST OF THE TIME ☐
3. A GOOD BIT OF THE TIME ☐
4. SOME OF THE TIME ☐
5. A LITTLE OF THE TIME ☐
6. HARDLY ANY OF THE TIME ☐
7. NONE OF THE TIME ☐

7. How often during the past 2 weeks did you have a feeling of fear or panic when you had difficulty getting your breath?
Please indicate how often you had a feeling of fear or panic when you had difficulty getting your breath by ticking one of the following options from the list below.
1. ALL OF THE TIME ☐
2. MOST OF THE TIME ☐

3. A GOOD BIT OF THE TIME ☐
4. SOME OF THE TIME ☐
5. A LITTLE OF THE TIME ☐
6. HARDLY ANY OF THE TIME ☐
7. NONE OF THE TIME ☐

8. What about fatigue? How tired have you felt over the last 2 weeks?
Please indicate how tired you have felt over the last 2 weeks by ticking one of the following options from the list below.
1. EXTREMELY TIRED ☐
2. VERY TIRED ☐
3. QUITE A BIT OF TIREDNESS ☐
4. MODERATELY TIRED ☐
5. SOMEWHAT TIRED ☐
6. A LITTLE TIRED ☐
7. NOT AT ALL TIRED ☐

9. How often during the last 2 weeks have you felt embarrassed by your coughing or heavy breathing?
Please indicate how much of the time you felt embarrassed by your coughing or heavy breathing by ticking one of the following options from the list below.
1. ALL OF THE TIME ☐
2. MOST OF THE TIME ☐
3. A GOOD BIT OF THE TIME ☐
4. SOME OF THE TIME ☐
5. A LITTLE OF THE TIME ☐
6. HARDLY ANY OF THE TIME ☐
7. NONE OF THE TIME ☐

10. In the last 2 weeks, how much of the time did you feel very confident and sure that you could deal with your illness?
Please indicate how much of the time you felt very confident and sure that you could deal with your illness by ticking one of the following options from the list below.
1. NONE OF THE TIME ☐
2. A LITTLE OF THE TIME ☐
3. SOME OF THE TIME ☐
4. A GOOD BIT OF THE TIME ☐
5. MOST OF THE TIME ☐
6. ALMOST ALL OF THE TIME ☐
7. ALL OF THE TIME ☐

11. How much energy have you had in the last 2 weeks?
Please indicate how much energy you have had by ticking one of the following options from the list below.
1. NO ENERGY AT ALL ☐
2. A LITTLE ENERGY ☐
3. SOME ENERGY ☐
4. MODERATELY ENERGETIC ☐
5. QUITE A BIT OF ENERGY ☐
6. VERY ENERGETIC ☐
7. FULL OF ENERGY ☐

12. In general, how much of the time did you feel upset, worried or depressed during the past 2 weeks?
Please indicate how much of the time you felt upset, worried or depressed during the past 2 weeks by ticking one of the following options from the list below.
1. ALL OF THE TIME ☐
2. MOST OF THE TIME ☐
3. A GOOD BIT OF THE TIME ☐
4. SOME OF THE TIME ☐
5. A LITTLE OF THE TIME ☐
6. HARDLY ANY OF THE TIME ☐
7. NONE OF THE TIME ☐

13. How often during the last 2 weeks did you feel you had complete control of your breathing problems?
Please indicate how often you felt you had complete control of your breathing problems by ticking one of the following options from the list below.
1. NONE OF THE TIME ☐
2. A LITTLE OF THE TIME ☐
3. SOME OF THE TIME ☐
4. A GOOD BIT OF THE TIME ☐
5. MOST OF THE TIME ☐
6. ALMOST ALL OF THE TIME ☐
7. ALL OF THE TIME ☐

14. How much of the time during the last 2 weeks did you feel relaxed and free of tension?
Please indicate how much of the time you felt relaxed and free of tension by ticking one of the following options from the list below.
1. NONE OF THE TIME ☐
2. A LITTLE OF THE TIME ☐
3. SOME OF THE TIME ☐
4. A GOOD BIT OF THE TIME ☐
5. MOST OF THE TIME ☐
6. ALMOST ALL OF THE TIME ☐
7. ALL OF THE TIME ☐

15. How often during the last 2 weeks have you felt low in energy?
Please indicate how often during the last 2 weeks you have felt low in energy by ticking one of the following options from the list below.
1. ALL OF THE TIME ☐
2. MOST OF THE TIME ☐
3. A GOOD BIT OF THE TIME ☐
4. SOME OF THE TIME ☐
5. A LITTLE OF THE TIME ☐
6. HARDLY ANY OF THE TIME ☐
7. NONE OF THE TIME ☐

16. In general, how often during the last 2 weeks have you felt discouraged or down in the dumps?
Please indicate how often during the last 2 weeks you felt discouraged or down in the dumps by ticking one of the following options from the list below.
1. ALL OF THE TIME ☐

2. MOST OF THE TIME ☐
3. A GOOD BIT OF THE TIME ☐
4. SOME OF THE TIME ☐
5. A LITTLE OF THE TIME ☐
6. HARDLY ANY OF THE TIME ☐
7. NONE OF THE TIME ☐

17. How often during the last 2 weeks have you felt worn out or sluggish?
Please indicate how much of the time you felt worn out or sluggish by ticking one of the following options from the list below.
1. ALL OF THE TIME ☐
2. MOST OF THE TIME ☐
3. A GOOD BIT OF THE TIME ☐
4. SOME OF THE TIME ☐
5. A LITTLE OF THE TIME ☐
6. HARDLY ANY OF THE TIME ☐
7. NONE OF THE TIME ☐

18. How happy, satisfied or pleased have you been with your personal life during the last 2 weeks?
Please indicate how happy, satisfied or pleased you have been by ticking one of the following options from the list below.
1. VERY DISSATISFIED, UNHAPPY MOST OF THE TIME ☐
2. GENERALLY DISSATISFIED, UNHAPPY ☐
3. SOMEWHAT DISSATISFIED, UNHAPPY ☐
4. GENERALLY SATISFIED, PLEASED ☐
5. HAPPY MOST OF THE TIME ☐
6. VERY HAPPY MOST OF THE TIME ☐
7. EXTREMELY HAPPY, COULD NOT HAVE BEEN MORE SATISFIED OR PLEASED ☐

19. How often during the last 2 weeks did you feel upset or scared when you had difficulty getting your breath?
Please indicate how often during the past 2 weeks you felt upset or scared when you had difficulty getting your breath by ticking one of the following options from the list below.
1. ALL OF THE TIME ☐
2. MOST OF THE TIME ☐
3. A GOOD BIT OF THE TIME ☐
4. SOME OF THE TIME ☐
5. A LITTLE OF THE TIME ☐
6. HARDLY ANY OF THE TIME ☐
7. NONE OF THE TIME ☐

20. In general how often during the last 2 weeks have you felt restless, tense or uptight?
Please indicate how often you have felt restless, tense or uptight by ticking one of the following options from the list below.
1. ALL OF THE TIME ☐
2. MOST OF THE TIME ☐
3. A GOOD BIT OF THE TIME ☐
4. SOME OF THE TIME ☐
5. A LITTLE OF THE TIME ☐

6. HARDLY ANY OF THE TIME ☐
7. NONE OF THE TIME ☐

Thank you very much for taking the time to complete this questionnaire.

FOR PR TEAM:

D	1 to 5	
F	8, 11, 15, 17	
E	6, 9, 12, 14, 16, 18, 20	
M	7, 10, 13, 19	

SCORED BY:
DATE:

Appendix 5 Quadriceps maximum voluntary contraction SOP

Royal Brompton & Harefield NHS
NHS Foundation Trust

**Quadriceps Maximum Voluntary Contraction**
SOP Reference: HHQMVC
Version Number: V3
Effective Date: 01/09/2017<br><br>Review Date: 01/09/2019
Authors: Sarah Jones, Cayley Smith, Dr Nick Hopkinson, Prof Mike Polkey, Dr William Man
Approved by: Dr William Man

Version	Date	Reason for Change
3	01/09/2017	Review and Update

Table of Contents	Page number
1. Purpose	3
2. Equipment	3
3. Procedure	3
4. Data Analysis	4
5. References	4

Purpose

The quadriceps muscle is of significant functional importance and may be affected by disuse, local disorders, or systematic problems. The force generated by a contracting muscle depends upon a number of factors, including the number of fibres stimulated and the muscle length.

It is important that the same procedure is always followed to obtain a Quadriceps Maximal Voluntary Contraction (QMVC) to ensure consistent, reproducible and standardised measurements.

Equipment

- Quadriceps chair with seatbelt, ankle strap and strain gauge.
- PowerLab recording unit
- PC
- LabChart software (or equivalent data acquisition system).

Procedure

QMVC is measured using the technique described by Edwards et al (1977). The subjects are seated in the quadriceps chair, with hip and knee flexion of 90 degrees. An inextensible strap is placed around the ankle, immediately proximal to the malleoli, adjusted to ensure the knee remains at 90 degrees flexion. The ankle strap is connected to a strain gauge mounted on the back of the chair, and runs perpendicular to both the ankle and the strain gauge. A seatbelt is secured across the subject's hips to stabilise the pelvis.

1) The procedure should be explained to the subject.
2) The subject should rest their hands/arms on top of their thighs, in their lap or across their chest to avoid the use of their upper limbs to increase force. Supervision should be provided to ensure the patient does not use other muscle groups to increase contraction force, such as holding on with hands, arching the back, lifting the buttocks off chair, or pushing the pelvis upwards against the hip strap.
3) The subject is asked to perform a warm-up consisting of four contractions at approximately 50% effort.
4) The subject is then asked to perform four contractions at approximately 75% effort, before commencing QMVC measures.
5) The QMVC measurements can then be commenced. Strong encouragement should be provided to obtain a maximal effort.
6) For the purpose of measurement, the maximal contraction needs to be maintained for several seconds.
7) A 20 second interval between maximal contractions should be observed to allow time for recovery.
8) Six QMVC contractions should be performed, with the best effort recorded. If force continues to increase with each effort then additional manoeuvres are permitted until fatigue is reached.

9) The QMVC is recorded as the maximal force able to be maintained for one full second.

Data Analysis

1. Open Labchart 7.
2. Select data file that you wish to analyse.
3. Select a point before the QMVC contraction that you want to analyse.

Select point in front of contraction for analysis.

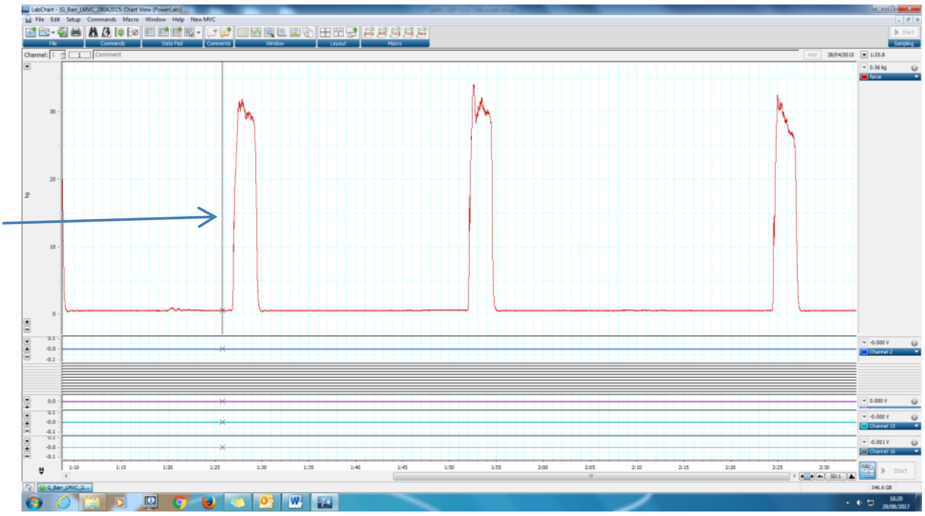

4. In the tool bar – under the MACRO tab – click NEW.

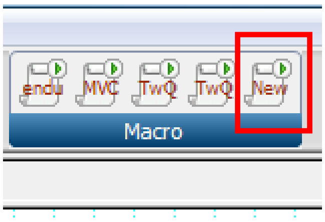

5. In the tool bar – under the DATA PAD tab – select the first icon.

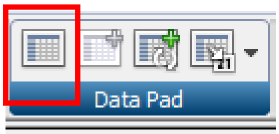

6. Click on the top row – then right click – select RECALCULATE ALL ROWS.
7. Looking down the first column – scroll down until you find the greatest value – this is your QMVC value.
8. Record on the data collection sheet.
9. Repeat steps 3-8 for each of the quadriceps contractions on the graph.
10. QMVC peak is the highest contraction recorded from all of the contractions.

Reference

Edwards, R. H. T., Young, A., Hosking, G. P., and Jones, D. A. (1977). Human skeletal muscle function: description of tests and normal values. Clinical Science and Molecular Medicine, 52, 283-290.

Appendix 6 Client Service Receipt Inventory

1. Please provide details of **hospital and residential services** you have used over the last three months.

Service	HAVE YOU HAD CONTACT?<br>NO 0	YES 1	Amount of use in the last 3 months	Any other information?
1-Specialist medicine outpatient visit (e.g. respiratory, cardiac, oncology)<br>(specify)	☐	☐	... attendances	
2-Other hospital outpatient visit<br>(specify)	☐	☐	... attendances	
3-Day hospital department (e.g. respiratory, cardiac, oncology)<br>(specify)	☐	☐	... attendances	
4-Rehabilitation (e.g. pulmonary,<br>cardiac) (specify)	☐	☐	... attendances	
5-A&E department	☐	☐	... attendances	
6-Nursing or residential home	☐	☐	... days	
7-Hospice	☐	☐	... days	
8-Inpatient ward (specify)	☐	☐	...days	
9-Critical care unit (intensive care or high dependency unit)	☐	☐	...days	
10-Other inpatient ward (specify)	☐	☐	... days	

2. Please provide details of **primary and community care services** you have used over the last three months.

Service	HAVE YOU HAD CONTACT?<br>No 0 / Yes 1		No. of contacts in last 3 months	Average duration (minutes)	Any other information?
	No 0	Yes 1			
1-General practitioner (GP)	☐	☐			
2-Other doctor, not including those in section 1 (specify) ____________	☐	☐			
3-Physiotherapist	☐	☐			
4-Social worker	☐	☐			
5-District nurse	☐	☐			
6-Practice nurse	☐	☐			
7-Rehabilitation (e.g. pulmonary, cardiac), not including rehabilitation included in section 1; (specify)	☐	☐	... attendances		
8-Psychologist	☐	☐			
9-Home help	☐	☐			
10-Occupational therapist	☐	☐			
11-Dietician	☐	☐			
12-Home palliative care/ hospice service	☐	☐			
13-Other therapists (e.g. speech)	☐	☐			

14-Other service (specify)	☐ ☐			

3. Please list any **investigations / diagnostic tests** you have received over the last three months.

Service	HAVE YOU HAD THIS TEST?<br>No 0	 Yes 1	No. in the last 3 months	Any other information?
1-Respiratory function test	☐	☐		
2-Chest x-ray	☐	☐		
3-Echocardiogram	☐	☐		
4-ECG	☐	☐		
5-Blood gas test	☐	☐		
6-Magnetic Resonance Image (MRI)	☐	☐		
7-CT/ CAT scan	☐	☐		
8-Blood test	☐	☐		
9-Other investigations/ tests	☐	☐		

4.
Please give details of any help you have received from **friends of family** members in the last three months **as a result of your illness**.

Type of help	HAVE YOU HAD HELP?<br>No 0	 Yes 1	Average no. of hours per week	Any other information?
1-Personal care (e.g. bathing, dressing)	☐	☐		
2-Help with medical procedures	☐	☐		
3-Help inside the home (e.g. cooking, cleaning)	☐	☐		

4-Help outside the home (e.g. shopping)	☐ ☐		
5-Time spent 'on-call' i.e. you need someone to stay with you if even they don't do specific jobs	☐ ☐		
6-Other (specify)	☐ ☐		

5. Please list below any **additional equipment** you have been using over the <u>last three months</u>.

**Type of help**	**HAVE YOU HAD HELP?**<br>**No 0** **Yes 1**	**Average no. of hours per day**	**Any other information?**
1- Ambulatory oxygen (oxygen cylinders)	☐ ☐		
2-Long-term oxygen therapy (oxygen concentrator)	☐ ☐		
3- Non-invasive ventilation (or CPAP)	☐ ☐		☐ overnight<br>☐ also during the day
4-Walking stick, rollator	☐ ☐		
5-Wheelchair	☐ ☐		
6-Feeding pump	☐ ☐		
7-Commode	☐ ☐		
8-Special bed	☐ ☐		
9-Bathroom or toilet adapted	☐ ☐		
10-Other equipment (specify)	☐ ☐		

6. Please list below use of any **medication** you have taken over the <u>last three months</u>.

Name of drug	Dosage (if known)	Dose frequency (e.g. daily)	How many weeks during the past three months did you take this drug (max 12)
1.	*mg*		
2.	*mg*		
3.	*mg*		
4.	*mg*		
5.	*mg*		

Appendix 7 Euro Qol – 5 Dimension – 5 Levels

Health Questionnaire

English version for the UK

Under each heading, please tick the ONE box that best describes your health TODAY.

MOBILITY

I have no problems in walking about ❑

I have slight problems in walking about ❑

I have moderate problems in walking about ❑

I have severe problems in walking about ❑

I am unable to walk about ❑

SELF-CARE

I have no problems washing or dressing myself ❑

I have slight problems washing or dressing myself ❑

I have moderate problems washing or dressing myself ❑

I have severe problems washing or dressing myself ❑

I am unable to wash or dress myself ❑

USUAL ACTIVITIES *(e.g. work, study, housework, family or leisure activities)*

I have no problems doing my usual activities ❑

I have slight problems doing my usual activities ❑

I have moderate problems doing my usual activities ❑

I have severe problems doing my usual activities ❑

I am unable to do my usual activities ❑

PAIN / DISCOMFORT

I have no pain or discomfort ❑

I have slight pain or discomfort ❑

I have moderate pain or discomfort ❑

I have severe pain or discomfort ❑

I have extreme pain or discomfort ❑

ANXIETY / DEPRESSION

I am not anxious or depressed ❑

I am slightly anxious or depressed ❑

I am moderately anxious or depressed ❑

I am severely anxious or depressed ❑

I am extremely anxious or depressed ❑

The best health
you can imagine

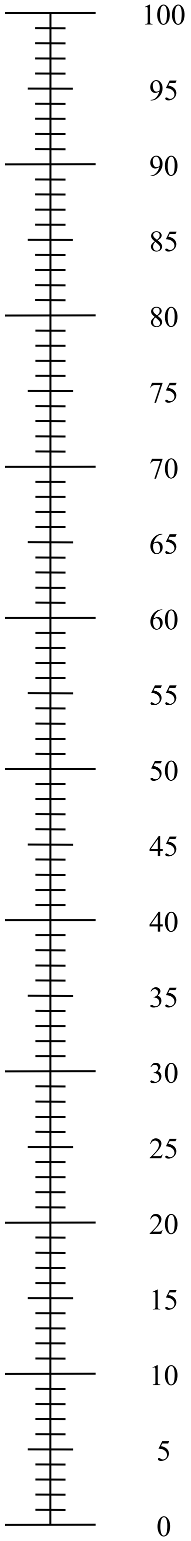

The worst health
you can imagine

We would like to know how good or bad your health is TODAY.

This scale is numbered from 0 to 100.

100 means the best health you can imagine.
0 means the worst health you can imagine.

Mark an X on the scale to indicate how your health is TODAY.

Now, please write the number you marked on the scale in the box below.

YOUR HEALTH TODAY =

Appendix 8 Global Rating of Change Questionnaire

Name Date

DOB Hospital Number

Thank you for completing the assessments at the end of your rehabilitation program

____

How do you feel your overall condition has changed after rehabilitation?

Please tick the one answer which fits you best –		
1)	I feel much better	
2)	I feel a little better	
3)	I feel no different	
4)	I feel a little worse	
5)	I feel much worse	

23.0 REFERENCES

1. Bolton CE, Blakey JD, Morgan MD. The British Thoracic Society guideline on pulmonary rehabilitation in adults: your opinion is noted. *Thorax* 2014;69(4):388-9. doi: 10.1136/thoraxjnl-2013-204754 [published Online First: 2014/01/17]
2. McCarthy B, Casey D, Devane D, et al. Pulmonary rehabilitation for chronic obstructive pulmonary disease. *The Cochrane database of systematic reviews* 2015;2 doi: 10.1002/14651858.CD003793.pub3 [published Online First: 2015/02/24]
3. RCP B. National COPD Audit Programme. Pulmonary Rehabilitation: Steps to Breathe Better 2016 [Available from: file:///C:/Users/cn805/Downloads/Nat%20COPD%20PR%20Clinical%20Audit%202015%20Full%20report_0.pdf accessed 4th December 2017.
4. Alison JA, McKeough ZJ. Pulmonary rehabilitation for COPD: are programs with minimal exercise equipment effective? *Journal of thoracic disease* 2014;6(11):1606.
5. Waterhouse J, Walters S, Oluboyede Y, et al. A randomised 2 x 2 trial of community versus hospital pulmonary rehabilitation, followed by telephone or conventional follow-up. *Health technology assessment (Winchester, England)* 2010;14(6):i-v, vii-xi, 1-140.
6. Holland AE, Mahal A, Hill CJ, et al. Home-based rehabilitation for COPD using minimal resources: a randomised, controlled equivalence trial. *Thorax* 2017;72(1):57-65.
7. Singh SJ, Morgan MD, Scott S, et al. Development of a shuttle walking test of disability in patients with chronic airways obstruction. *Thorax* 1992;47(12):1019-24.
8. BTS. Quality Standards for Pulmonary Rehabilitation. *British Thoracic Society Reports* 2014;6(2)
9. Charlson ME, Pompei P, Ales KL, et al. A new method of classifying prognostic comorbidity in longitudinal studies: development and validation. *Journal of chronic diseases* 1987;40(5):373-83.
10. Bestall JC, Paul EA, Garrod R, et al. Usefulness of the Medical Research Council (MRC) dyspnoea scale as a measure of disability in patients with chronic obstructive pulmonary disease. *Thorax* 1999;54(7):581-6. [published Online First: 1999/06/22]
11. ARTP. ARTP Handbook in Spirometry. 3rd Edition ed. United Kingdom: ARTP 2010.
12. Quanjer PH, Tammeling GJ, Cotes JE, et al. Lung volumes and forced ventilatory flows. Report Working Party Standardization of Lung Function Tests, European Community for Steel and Coal. Official Statement of the European Respiratory Society. *Eur Respir J Suppl* 1993;16:5-40. [published Online First: 1993/03/01]
13. Patel MS, Mohan D, Andersson YM, et al. Phenotypic characteristics associated with reduced short physical performance battery score in COPD. *CHEST Journal* 2014;145(5):1016-24.
14. Holland AE, Spruit MA, Troosters T, et al. An official European Respiratory Society/American Thoracic Society technical standard: field walking tests in chronic respiratory disease. *European Respiratory Journal* 2014;44(6):1428-46.
15. Williams JE, Singh SJ, Sewell L, et al. Development of a self-reported Chronic Respiratory Questionnaire (CRQ-SR). *Thorax* 2001;56(12):954-59.
16. Man WD, Hopkinson NS, Harraf F, et al. Abdominal muscle and quadriceps strength in chronic obstructive pulmonary disease. *Thorax* 2005;60(9):718-22.
17. Nolan CM, Longworth L, Lord J, et al. The EQ-5D-5L health status questionnaire in COPD: validity, responsiveness and minimum important difference. *Thorax* 2016:thoraxjnl-2015-207782.
18. Piaggio G, Elbourne DR, Pocock SJ, et al. Reporting of noninferiority and equivalence randomized trials: extension of the CONSORT 2010 statement. *Jama* 2012;308(24):2594-604.
19. Wangge G, Putzeist M, Knol MJ, et al. Regulatory scientific advice on non-inferiority drug trials. *PloS one* 2013;8(9):e74818.

20. Singh SJ, Jones PW, Evans R, et al. Minimum clinically important improvement for the incremental shuttle walking test. *Thorax* 2008;63(9):775-7.
