# Supplementary material for: Minimal vs Specialized Exercise Equipment for Pulmonary Rehabilitation: A Randomized Clinical Trial
Source: JAMA Netw Open. 2025 Aug 12;8(8):e2526616. doi: 10.1001/jamanetworkopen.2025.26616 (PMC12344533; doi:10.1001/jamanetworkopen.2025.26616)
Supplement: Supplement 2. — Statistical Analysis Plan [file jamanetwopen-e2526616-s002.pdf]

MIster

MInimal versus SpecialisT Equipment in the delivery of pulmonary Rehabilitation (MISTER): a randomised controlled trial

Statistical Analysis Plan

Version 2.0

Version 1 started: 23/10/2018

Based on MISTER Protocol Version 5

Authors: Miss Peihan Yu, Dr. Rebecca Wilson, Mr Callum Glen

Reviewed by: Professor Wei Gao, Dr Francesca Fiorentino

Senior Statistician: Dr Francesca Fiorentino

ISRCTN: 16196765

Chief Investigators

Dr William Man
Harefield Respiratory Research Group, Royal Brompton and Harefield Clinical Group, GSTT
National Heart and Lung Institute, Imperial College London
Harefield Pulmonary Rehabilitation Unit, GSTT
w.man@rbht.nhs.uk

Dr Matthew Maddocks PhD
Senior Lecturer and Specialist Physiotherapist
Cicely Saunders Institute, Division of Palliative Care, Policy and Rehabilitation
King's College London
matthew.maddocks@kcl.ac.uk

Dr Claire Nolan
Lecturer in Physiotherapy
Brunel University London
Claire.nolan@brunel.ac.juk

Statisticians

Callum Glen
Trial Statistician
King's Clinical Trial Unit and Nightingale Saunders Faculty of Nursing, Midwifery and Palliative Care
King's College London
callum.glen@kcl.ac.uk

Dr Francesca Fiorentino
Senior Lecturer in Clinical Trials Statistics
King's Clinical Trials Unit and Nightingale Saunders Faculty of Nursing, Midwifery and Palliative Care
King's College London
francesca.fiorentino@kcl.ac.uk

Version number	Date	Reason for Revision	Revised by
1.1	29/11/21	Revision from protocol update	CG
1.2	27/05/22	Revision from FF comments	CG
1.3	06/09/22	Revision from FF comments	CG
1.4	11/03/23	Comments from Trial team	CG
1.5	12/04/23	Comments from FF	CG

Signatures

Dr William Man

____ Date ____________

Dr Matthew Maddocks

Matthew Maddocks Digitally signed by Matthew Maddocks Date: 2023.04.17 15:42:20 +01'00'

Date ____________

Dr Claire Nolan

Claire nolan Digitally signed by Claire nolan Date: 2023.04.13 10:08:00 +01'00'

Date ____________

Dr Francesca Fiorentino

Francesca Fiorentino (k2145850) Digitally signed by Francesca Fiorentino (k2145850) Date: 2023.04.26 10:35:01 +01'00'

Date ____________

Mr Callum Glen

Callum Glen (k2149490) Digitally signed by Callum Glen (k2149490) Date: 2023.04.13 09:34:01 +01'00'

Date ____________

Acronyms

Acronym	
95% CI	95% Confidence Interval
AaR	Attrition at Random
ADD	Attrition Due to Death
ADI	Attrition Due to Illness
AE	Adverse Event
BTS	British Thoracic Society
CACE	Complier Average Causal Effect
CI	Chief Investigator
CONSORT	Consolidated Standards of Reporting Trials
COPD	Chronic Obstructive Pulmonary Disease
CRQ-D	Chronic Respiratory Questionnaire Dyspnoea domain
CRQ-T	Chronic Respiratory Questionnaire Total score
FDR	False Discovery Rate
FEV_1	Forced Expiratory Volume (first second)
FVC	Forced Vital Capacity
GEE	Generalised Estimation Equation
GROC	Global Rating of Change
GROS	Global Rating of Satisfaction
ILD	Interstitial Lung Disease
ISW	Incremental Shuttle Walk test
ITT	Intention to Treat
KCTU	King's Clinical Trials Unit
MCID	Minimal Clinically Important Difference
MORECARE	Methods for Evaluating Service Delivery Models for End-of-Life Care
MRC	Medical Research Council
PP	Per Protocol
PR	Pulmonary Rehabilitation
QMVC	Quadriceps Maximum Voluntary Contraction
SAE	Serious Adverse Event
SAP	Statistical Analysis Plan
SD	Standard Deviation
Spo2	Oxygen Saturation

Contents

1 Study summary 7
2 Description of the trial 7
2.1 Overall trial design 7
2.2 Trial schema 8
2.3 Eligibility criteria 9
2.3.1 Inclusion criteria 9
2.3.2 Exclusion criteria 9
2.4 Randomisation procedure 9
2.5 Study treatment 10
2.5.1 Control group: PR-specialist 10
2.5.2 Intervention group: PR-min 10
2.6 Outcomes 11
2.6.1 Primary outcome 11
2.6.2 Secondary outcomes 11
2.7 Visit windows 12
2.8 Data collection 12
2.8.1 Primary outcome measure 12
2.8.2 Secondary outcomes measures 13
2.9 Sample size estimation 15
2.10 Brief description of proposed analyses 15
2.11 Timing of Final Analysis 16
3 Data analysis plan: Data description 16
3.1 Recruitment and representativeness of recruited patients 16
3.1.1 Figure 1. Template CONSORT diagram for the MISTER trial 17
3.2 Baseline comparability of randomised groups 18
3.3 Adherence to allocated treatment and treatment fidelity 18
3.4 Loss to follow-up and other missing data 18
3.5 Adverse event reporting and Protocol Deviations 19

3.6 Descriptive statistics for outcome measures 19
3.7 Assessment of outcome measures (unblinding) 20
4 Data analysis plan: Inferential analysis 20
4.1 Main analysis of treatment differences 20
4.1.1 Analysis of primary outcome 20
4.1.2 Analysis of secondary outcomes 21
4.1.3 Statistical considerations 21
4.1.4 Analysis of adverse events and survival 22
4.1.5 Sensitivity analyses 23
4.2 Interim and subgroup analyses 23
4.3 Exploratory analyses 24
5 Software 24
6 Amendment 24
7 References 25
8 Appendices 26
8.1 MISTER - Forms to expect completed depending on trial status 26
8.1.1 Visit 1 27
8.1.2 Visit 2 27
8.1.3 Visit 3 29
8.2 MISTER - Tables to be included in the statistical report 31
8.2.1 Table A1 - Summary table of baseline characteristics by treatment arm 31
8.2.2 Table A2 – Summary of baseline outcome measures 33
8.2.3 Table A3 – Summary of baseline characteristics by the validity of ISW score at visit 2 34
8.2.4 Table A4 – Summary of baseline outcome measures by validity of ISW test score 36
8.2.5 Table A5 - Summary table of primary outcome statistics 37
8.2.6 Table A6 – Summary table of secondary outcome statistics 38
8.2.7 Table A7 - Primary outcome analysis 40
8.2.8 Table A8 – Primary outcome analysis – secondary analysis 40

8.2.9 Table A9 – Secondary outcome analysis (pre- to post-PR)....41
8.2.10 Table A10 – Secondary outcome analysis (pre-PR to 12 months)....42
8.2.11 Table A11 – Compliance and Adherence table....43
8.2.12 Table A12 – Sensitivity analyses for primary outcome data....43
8.2.13 Table A13 – PR completion rate and attendance....44
8.2.14 Table A14 – Minimal clinically important difference – Primary Outcome....44
8.2.15 Table A15 – Minimal Clinically Important Difference – Secondary outcomes....45
8.3 MISTER - Tables to be included in the statistical report....46
8.3.1 Figure A1....46
8.3.2 Figure A2....46
8.3.3 Figure A3....46
8.3.4 Figure A4....46
8.3.5 Figure A5....46
8.3.6 Figure A6....46

1 Study summary

The aim of the study is to determine whether an eight-week supervised pulmonary rehabilitation (PR) programme using minimal exercise equipment (PR-min) is non-inferior to a standard eight-week supervised PR programme delivered using specialist exercise equipment (PR-specialist) in terms of health benefits for patients with chronic respiratory disease.

2 Description of the trial

2.1 Overall trial design

This study is a parallel, two-group, assessor- and statistician-blinded, randomised, non-inferiority trial of PR programmes, comparing a PR programme that uses minimal exercise equipment (PR-min, intervention group), to one that uses specialist exercise equipment (PR-specialist, control group). Participants are randomised at the individual level with a 1:1 allocation to either PR-min or PR-specialist. Both interventions comprise two supervised sessions per week for eight weeks delivered by the same team.

2.2 Trial schema

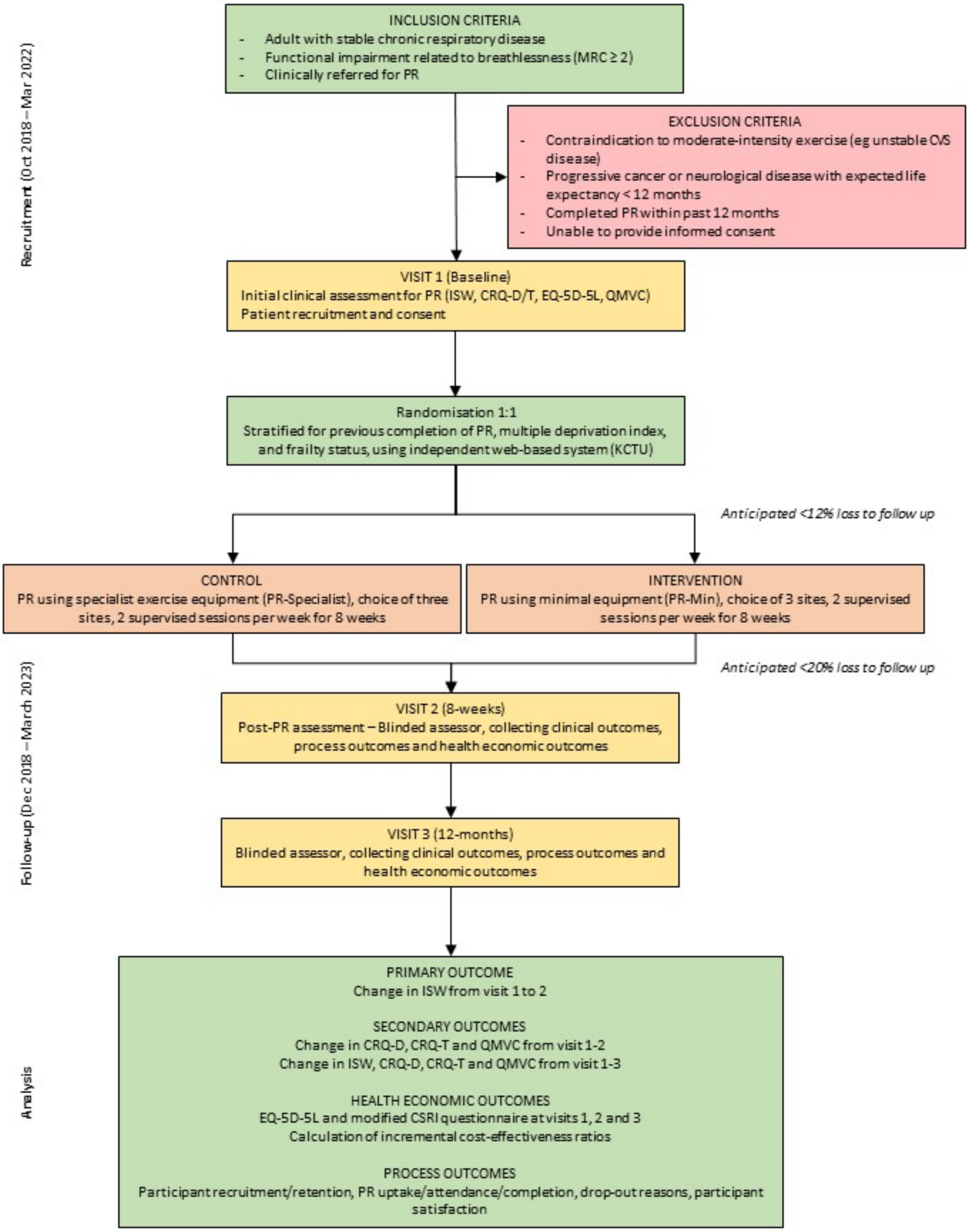

2.3 Eligibility criteria

2.3.1 Inclusion criteria

1. Adults >18 years of age of either sex.
2. Physician diagnosis of stable chronic respiratory disease - typically COPD, interstitial lung disease (ILD), bronchiectasis, chronic asthma, or chest wall disease.
3. Referred for PR in line with BTS guidelines (i.e., ambulatory – can walk ⩾5 metres, functional impairment related to breathlessness, typically MRC dyspnoea score ⩾2)
4. Able to communicate verbally and respond to questions in written English.

2.3.2 Exclusion criteria

1. Contra-indication to moderate intensity physical exercise e.g., unstable cardiovascular disease.
2. Progressive cancer or neurological disorder with expected life expectancy less than 12 months.
3. Completed PR within previous 12 months.
4. Unable to provide informed consent.

2.4 Randomisation procedure

Consenting participants are randomised at the individual level with a 1:1 allocation, using an independent web-based system provided by the United Kingdom Clinical Research Collaboration registered King's Clinical Trials Unit (KCTU), to receive either "usual care" (PR-specialist) or intervention (PR-min).

Randomisation by minimization ensures that participants are stratified according to the following:

1. Previous completion of PR (yes / no)
2. Multiple deprivation index (most deprived quintile of index: yes / no)
3. Frailty status (Short Physical Performance Battery score < 10 / ⩾10).

A proportion of patients will be entered initially using simple randomisation in order to create a level of initial imbalance, then the minimisation algorithm maintains a level of randomness in order to preserve pre-randomisation allocation concealment.

Once randomised, the system automatically generates a full audit trail of the process and send emails to relevant investigators in a blinded or unblinded format, depending on their role. For each arm, participants are provided with a choice of three sites to undertake PR.

Owing to the nature of the interventions, participants and providers of the intervention are not blinded. However, post-rehabilitation assessments are performed by a researcher not involved in the delivery of either intervention arm, and blinded to group allocation. The junior trial statistician is not blinded to group allocation; the senior trial statistician is blinded.

2.5 Study treatment

Both treatment groups comprise two supervised sessions per week for eight weeks delivered by the same team.

2.5.1 Control group: PR-specialist

The control intervention is current gold-standard clinical practice. PR-specialist comprises an eight-week outpatient exercise and multi-disciplinary self-management education programme, with two supervised and at least one additional home session each week, delivered according to the British Thoracic Society Quality Standards. Supervising staff comprise specialist respiratory therapists with a minimum of two years' experience in PR. Available equipment include treadmills, cycle ergometers, cross-trainers, specialist lower limb resistance equipment (such as leg press, knee extension). Each supervised session consists of one hour of exercise (at least 30 minutes of aerobic exercise) and 45 minutes of education. Study participants in the control group have a choice of centres across northwest London.

Initial walking speed prescription on the treadmill is 80% of predicted peak oxygen consumption based on baseline ISW performance, whilst initial endurance cycling is initially set at 60% peak workload on a cycle ergometer with the aim of patients completing ten minutes of continuous training. Lower limb resistance training comprises of two sets of ten leg press repetitions on specialist resistance equipment performed with an initial training load of 60% one-repetition maximum. Similarly, two sets of ten bilateral knee extension repetitions are performed on specialist resistance equipment at an initial training load of 60% one-repetition maximum. This is supplemented with sit-to-stand sets, plus knee lifts/extension and hip abduction with appropriate ankle weights up to 10kg. Upper limb resistance training comprises biceps curls, shoulder press and upright row with free weights or Therabands^{TM} (red to black). Exercise training is individualized and regularly progressed (either in duration or intensity) according to standard operating procedures set by the Royal Brompton Hospital manual, with targets reviewed at each supervised session. Education is delivered by a multi-disciplinary team with topics chosen to develop patients' understanding and holistic management of their disease.

2.5.2 Intervention group: PR-min

PR-min also comprises an eight-week outpatient exercise and multidisciplinary self-management education programme, with two supervised and at least one additional home session each week, delivered according to the BTS Quality Standards. As per PR-specialist, supervising staff comprise specialist respiratory therapists with at least two years' experience of delivering pulmonary rehabilitation independently. There is no access to treadmills, cycle ergometers, or specialist resistance equipment. Available exercise equipment includes portable steppers, portable pedals, hand and ankle weights (up to 5kg), and Therabands^{TM} (red to black). Each supervised session consists of one hour of exercise (at least 30 minutes aerobic exercise) and 45 minutes of education. Study participants in the intervention group have a choice of centres across northwest London.

Initial walking speed prescription is 80% of predicted peak oxygen consumption based on baseline ISW performance. Participants are provided with their own stopwatch and given time targets to complete a walking course of known distance. Although the resistance of the portable steppers and pedals can be manually adjusted, this cannot be objectively quantified. Initial prescription is set at "level 1" but individually adjusted to find an intensity where patients can complete ten minutes of continuous training with a target modified Borg breathless score of 3-4 and a Borg rating of Perceived Exertion of 13-15 (on a scale of 6-20). Resistance training includes functional activities such as sit-to-stand and step-ups as well as Theraband^{TM} based exercises such as sitting knee extension, leg press and hip flexion as well as standing hip extension, squats, chest press and lateral raise. Progression is through the use of hand/ankle weights and increasing resistant Therabands^{TM} (from red to black). Exercise training is individualized and regularly progressed (either in duration or intensity)

with targets reviewed at each supervised session. Education is delivered by a multi-disciplinary team as per PR-specialist.

2.6 Outcomes

2.6.1 Primary outcome

The primary outcome of the study is change in exercise capacity measured by the incremental shuttle walk test (ISW) distance from pre-PR (visit 1/baseline) to post-PR (visit 2 at 8-weeks post randomisation), calculated as post-PR incremental shuttle walk test (ISW) distance minus pre-PR incremental shuttle walk test (ISW) distance. ISW is also collected at 12-month (visit 3) follow-up.

2.6.1.1 Primary outcome measure: Incremental Shuttle Walk test

For the Incremental Shuttle Walk (ISW) the participant is required to walk around two cones set 9 metres apart in time to a set of auditory beeps played on a CD. The participant keeps walking until they can no longer keep up with the beeps or are too breathless to continue. At that point, the test ends. The number of shuttles between the cones is recorded and the distance covered during the test is measured.

2.6.2 Secondary outcomes

- Change in breathlessness measured using the dyspnoea domain of the Chronic Respiratory Questionnaire (CRQ-D) from pre-PR to post-PR and 12-month follow-up.
- Change in disease-specific health related quality of life measured using the CRQ total score (CRQ-T) from pre-PR to post-PR and 12-month follow-up
- Change in lower limb muscle strength measured using isometric quadriceps maximum voluntary contraction (QMVC) from pre-PR to post-PR and 12-month follow-up.
- Trial process indicators at appropriate stages of the trial
    - Number of patients recruited to the trial
    - Proportion of patients that uptake, adhere to, and complete PR
    - Reasons for PR non-completion
    - Patients' satisfaction levels measured using the Global Rating of Change Questionnaire and Global Rating of Satisfaction
- Health economics measures for cost and cost-effectiveness of the intervention (to be conducted by a Health Economist at the Cicely Saunders Institute at King's College, London).
- Adverse events

2.6.2.1 Chronic Respiratory Questionnaire

The Chronic Respiratory Questionnaire (CRQ is an interviewer-administered questionnaire measuring both physical and emotional aspects of chronic respiratory disease. 20 items are split into 4 categories (Dyspnoea, fatigue, emotional function, mastery), with a 7-point scale for each item which gives a score when added together and then averaged over the number of items.

For the CRQ-D, the scores for each item will be added together, and averaged over 5 items, these items being 1-5 of the total 20. The CRQ-D measures the dyspnoea of the participant. For the CRQ-F, the scores for each item will be added together and averaged over 4 items, these items being 8, 11, 15 and 17. The CRQ-F measures the fatigue of the patient. For the CRQ-E, the scores for each item will be added together and averaged over 7 items, these items being 6, 9, 12, 14, 16, 18 and 20. The CRQ-E measures the emotional function of the participant. For the CRQ-M, the scores for each item will be added together and averaged

over 4 items, these items being 7, 10, 13 and 19. The CRQ-M measures the mastery of the participant.

The CRQ-T will be the sum of all items from all subscales, averaged over the number of items, which is 20 in this case. The CRQ-T measures the disease specific health related quality of life for each participant.

2.6.2.2 *Quadriceps Maximum Voluntary Contraction*

To perform the Quadriceps Maximum Voluntary Contraction (QMVC) test, participants sit in a purpose-built chair with an inextensible strap which connects the ankle to a strain meter (measured in kg). The force is then applied by the participant by raising their leg and the reading from the meter is taken.

2.7 Visit windows

Outcome measures and patients' characteristics are recorded at three time points during the trial, as appropriate. They are:

- Visit 1 – initial assessment for PR (pre-PR)
- Visit 2 – immediate assessment after PR at eight weeks from Visit 1 (post-PR)
- Visit 3 – follow-up assessment at 12 months from Visit 1 (12-month follow-up)

Safety data are recorded throughout the trial whenever necessary.

2.8 Data collection

A detail description of the study assessments used can be found in the MISTER Protocol (version 5).

The treatment period is 8-weeks for both PR-min and PR-specialist. In each week of the programme participants receive 2 supervised sessions and are expected to carry out at least one additional home session, making a total of at least 24 sessions over the 8-weeks, 16 supervised and at least 8 additional home sessions.

Follow-up data is collected at 8-weeks, immediately after finishing the treatment course (visit 2, primary endpoint), and at 12 months (visit 3, secondary endpoint). Follow-up data are acceptable when collected at the visit in question.

2.8.1 *Primary outcome measure*

Outcome	Variable	Timepoint(s)	Variable type	Assessment / Description
Exercise capacity	ISW distance	Visit 1, Visit 2, Visit 3	Continuous	Incremental Shuttle Walk (ISW) test, conducted as

				part of routine clinical assessment.<br><br>The ISW distance in metres will be used in the analysis.

2.8.2 Secondary outcomes measures

Outcome	Variable	Timepoint(s)	Variable type	Assessment / Description
Breathlessness / Dyspnoea	CRQ-Dyspnoea domain score	Visit 1, Visit 2, Visit 3	Continuous	Dyspnoea domain of the 20-item Chronic Respiratory Questionnaire (CRQ-D), conducted as part of routine clinical assessment.<br><br>Items 1 to 5 form the Dyspnoea domain, with scores ranging 1 to 7; a lower score would mean higher breathlessness.
Fatigue	CRQ – Fatigue domain	Visit 1, Visit 2, Visit 3	Continuous	Fatigue domain of the 20-item Chronic Respiratory Questionnaire (CRQ-F), conducted as part of routine clinical assessment.<br><br>Items 8, 11, 15 and 17 form the fatigue domain, with scores ranging from 1 to 7, a lower score means higher fatigue.
Emotional Function	CRQ – Emotional	Visit 1, Visit 2, Visit 3	Continuous	Emotional Function domain of the 20-item Chronic Respiratory

	Function domain			Questionnaire (CRQ-E), conducted as part of routine clinical assessment.<br><br>Items 6, 9, 12, 14, 16, 18 and 20 form the emotional function domain, with scores ranging from 1 to 7, a lower score means higher emotional function.
Mastery	CRQ – Mastery	Visit 1, Visit 2, Visit 3	Continuous	Mastery domain of the 20-item Chronic Respiratory Questionnaire (CRQ-M), conducted as part of routine clinical assessment.<br><br>Items 7, 10, 13 and 19 form the mastery domain, with scores ranging from 1 to 7, a lower score means higher mastery.
Disease-specific health-related quality-of-life	CRQ-Total score	Visit 1, Visit 2, Visit 3	Continuous	Chronic Respiratory Questionnaire total score (CRQ-T), conducted as part of routine clinical assessment to measure symptom burden.<br><br>It contains 20 items, with scores ranging from 1 to 7; a lower score would mean a higher symptom burden i.e. lower disease-specific health-related quality-of-life.
Lower limb muscle strength	Peak Quadriceps Maximum Voluntary Contraction value	Visit 1, Visit 2, Visit 3	Continuous	Quadriceps Maximum Voluntary Contraction (QMVC) test.<br><br>The best QMVC value in kilograms will be used in the analysis.

2.9 Sample size estimation

Previous audits of the Harefield PR service have shown that participants undergoing PR-specialist achieve a mean (SD) change in ISW of 58 meters (SD 67m). The null hypothesis is that the experimental treatment (PR-min) is inferior to the standard treatment (PR-specialist). The alternative hypothesis is that PR-min is not inferior to PR-specialist. The non-inferiority margin will be defined as half the known MCID using the fixed-margin method with a preserved effect of 50% as recommended by previous guidance, including from the United States Food and Drug Administration [1 2]. The MCID of the ISW is 47.5 metres[3], and therefore 24 meters will be considered the non-inferiority margin. If there is truly no difference between PR-min and PR-specialist, then a minimum of 246 patients (123 in each group) is required to be 80% sure that the lower limit of a one-sided 97.5% CI (or equivalently a 95% two-sided CI) will be above the non-inferiority limit of -24 meters. Based on audit data, we anticipate 32% drop out from PR (12% from assessment to starting PR, and 20% from starting PR to completing PR). Taking into account drop-out, the minimum sample size required for analysis will be 362 patients (181 patients per group).

Recruitment will take place from the Harefield Hospital PR programme which receives 1000 referrals a year. We anticipate 700 eligible patients for recruitment per year. Based on experience from previous studies, the research recruitment and consent rate is consistently above 65%. Our proposal sample size is 362 patients. Using a conservative estimate of 40% recruitment rate, we anticipate recruitment of 362 patients will take 15-18 months.

2.9.1 Impact of COVID-19

Owing to the impact of the COVID-19 pandemic on the study (described in the Amendment, section 6), the study target sample size has been increased by a further 74 participants. The number was decided upon the fact that 60 participants had their intervention (PR-min or PR-specialist) interrupted by a COVID lockdown. Participants were interrupted because of a lockdown being introduced while they still had PR sessions to attend as part of their intervention. Hence adding 60 participants, and accounting for potential loss to follow up, increased the sample size by 74 participants, for a total of 436 participants (218 patients per group).

2.10 Brief description of proposed analyses

Analyses will be carried out by the trial statistician. Primary data analysis will be conducted under the intention-to-treat (ITT) assumptions; a secondary data analysis will be performed under per-protocol (PP) assumptions.

Under ITT assumptions, all randomised patients will be included in the analysis; they will be analysed according to the treatment they were randomised to, irrespective of the treatment they had actually received, and any other protocol violations.

Under PP assumptions, all patients who had adhered to the protocol (ie, adhere to and complete PR) will be included in the analysis. Patients who had not adhered to the protocol will be excluded from the analysis.

The proposed analyses include:

1. Descriptive analysis of baseline and follow-up data by trial arm;
2. Main analysis for the effect of PR-specialist and PR-min on the primary outcome, using all available data and adjusting for baseline score and for variables for which treatment-group substantial imbalance is found;
3. Analysis for effects of PR on the secondary outcomes;
4. Descriptive analysis of the missing data, exploration of missing patterns by trial arm, missing data will be imputed as appropriate;
5. Sensitivity analysis (including per protocol analysis) to assess the robustness of the trial findings for primary outcome.

2.11 Timing of Final Analysis

The outcomes of the trial, both primary and secondary, will be analysed after data for the 12-month follow up point have been collected and the database has been locked.

3 Data analysis plan: Data description

3.1 Recruitment and representativeness of recruited patients

A CONSORT flow chart will be constructed – see Figure 1. This will include the number of eligible patients, number of patients agreeing to enter the trial, number of patients refusing, then by treatment arm: the number of patients not/inadequately/adequately treated OR compliant/non-compliant, the number continuing through the trial, the number withdrawing, the number lost to follow-up and the numbers excluded/analysed. Appendix figure A1 details the graph to be produced to show the recruitment over time.

3.1.1 Figure 1. Template CONSORT diagram for the MISTER trial

3.2 Baseline comparability of randomised groups

Summary tables (descriptive statistics and/or frequency tables) by trial arm will be provided for all baseline variables, including demographic and clinical characteristics and baseline measures of primary and secondary outcomes. We will also produce the above summary tables by those with and without a valid ISW distance score at visit 2 (primary outcome) where a valid ISW score is a score between 0 and 1500. Other values are deemed invalid. Continuous variables will be summarized with descriptive statistics (mean, standard deviation, median, interquartile range, minimum and maximum where appropriate). Frequency counts and percentage of subjects within each category will be provided for categorical data. No significance testing for the difference in baseline characteristics will be carried out. Appendix table A1 is a blank table for the summary of baseline characteristics by treatment arm and overall, and appendix table A2 is a summary of baseline questionnaire and test scores, again by treatment arm and overall. Appendix table A3 is a blank table for the summary of baseline characteristics by the validity of ISW distance score at visit 2, and appendix table A4 is a blank table of baseline questionnaire and test scores by the validity of ISW distance score at visit 2.

3.3 Adherence to allocated treatment and treatment fidelity

Adherence versus non-adherence with the treatment will be described by treatment arm and overall using baseline variables. Adherence and non-adherence will be determined by analysing the standard clinical records of the PR teams and direct observation by the trial co-ordinator. Adherence is defined as attending 8 or more sessions out of the maximum 16 supervised pulmonary rehabilitation sessions, irrespective of the assigned intervention. This will be recorded in both intervention arms.

3.4 Loss to follow-up and other missing data

The reasons for withdrawal from the trial will be summarised, categorised into reasons. There are a number of reasons that the participant could withdraw from the study. These are:

- The participant no longer wishes for data to be collected
- Unable to locate or contact the participant
- The participant is no longer able to travel to the centre
- Death of the participant
- Other adverse event/other reason

The reasons for participants withdrawal from the intervention (PR) will also be summarised. There are a number of reasons why a participant could withdraw from the intervention. These are:

- Participant admitted to hospital (underlying respiratory condition)
- Participant admitted to hospital (other reason)
- Exacerbation of underlying lung disease (not admitted to hospital)
- Unwell (for other reasons)
- Family commitments
- Declined PR
- Death of participant
- Unable to contact participant
- Participant wanted to change PR site
- Other reasons

The proportions of participants missing each variable will be summarised in each arm and at each visit. The baseline characteristics of those missing follow up will be compared to those with complete follow up.

3.5 Adverse event reporting and Protocol Deviations

Adverse events (AE) and serious adverse events (SAE) will be summarised.

An Adverse Event (AE) is any untoward medical occurrence in a patient or clinical study subject who is administered a treatment and which does not necessarily have a causal relationship with this treatment (i.e. any unfavourable or unintended change in the structure (signs), function (symptoms), or chemistry (lab data) in a subject to whom a treatment/study procedure has been administered, including occurrences unrelated to that product/procedure/device).

A Serious Adverse Event (SAE) is defined as an untoward occurrence that:

a) results in death,
b) is life-threatening (places the subject, in the view of the investigator, at immediate risk of death),
c) requires hospitalization or prolongation of existing hospitalization (hospitalization is defined as an inpatient admission, regardless of length of stay; even if it is a precautionary measure for observation; including hospitalization for an elective procedure, for a pre-existing condition),
d) results in persistent or significant disability or incapacity (substantial disruption of one's ability to conduct normal life functions),
e) Consists of a congenital abnormality or birth defect (in offspring of subjects or their parents taking the study drug regardless of time of diagnosis) or,
f) is otherwise considered medically significant by the investigator.

Important medical events that may not be immediately life-threatening or result in death or hospitalization but may jeopardize the subject or may require intervention to prevent one of the outcomes listed in the definition of serious will also be considered serious.

The Investigator and research team are responsible for reporting events to the Research Office immediately and/or within 24 hours of becoming aware of the event. An SAE occurring to a research participant will be reported to the Research Ethics Committee that gave a favorable opinion of the study. Classification and causality of AE's will be conducted by the project manager and reviewed by the CI. The CI cannot downgrade the project manager's classification and if there is disagreement which cannot be resolved during formal discussion the assessment of the project manager will be accepted. The CI, can however, upgrade the seriousness of an event without consultation with the project manager.

All AE's will be recorded in the hospital notes and case report forms (CRF). If the investigator suspects that the disease has progressed faster due to the administration of the study treatment/procedure, then he/she will report this as an unexpected AE to the sponsor and the REC.

The following adverse/serious adverse events will be reported by trial arm and by selected variables:

- Any untoward medical occurrence in a patient
- Number of deaths
- Number of hospital admissions
- Length of hospital stay

Any reported protocol deviations will be tabulated.

3.6 Descriptive statistics for outcome measures

The outcome measures will be described by trial arm, at visit 1, visit 2 and visit 3, using standard descriptive statistics (mean and standard deviation, median and interquartile range,

or count and proportion as appropriate) using the blank tables A5 and A6 in the appendix. We will graphically display the means for primary and selected secondary outcomes over time by treatment group, detailed in appendix figure A2 and A3.

3.7 Assessment of outcome measures (unblinding)

A log will be kept of who conducted the baseline assessment for each participant. The trial will then be conducted so that, at the follow-up visits, assessments are not conducted by the same individual that conducted the baseline assessment.

4 Data analysis plan: Inferential analysis

4.1 Main analysis of treatment differences

The main statistical analyses will estimate the difference in mean outcomes between patients randomised to PR-specialist and PR-min by intention to treat at 12 weeks. Group difference estimates and associated confidence intervals will be reported. The significance level is set at one-sided significance level of 0.025.

4.1.1 Analysis of primary outcome

The primary outcome is the measure of ISW distance in metres at visit 2. A change score will be generated, subtracting the ISW distance at visit 2 from that at visit 1. The analysis will be on an intention-to-treat (ITT) basis using one-sided two independent sample t-test or non-parametric equivalent. The effect estimates and their 95% confidence intervals will be reported. Where regression diagnostic checks suggest model distributional assumptions are not met, confidence-intervals and test statistics will be calculated using boot-strap methods. Box-plots for each treatment group at each time point will be generated. Appendix table A7 shows how these data will be displayed in the report, and appendix figure A4 details the box-plot to be produced.

The primary outcome is the measure of ISW distance in metres at visit 2. A change variable will be generated, subtracting the ISW distance at visit 2 from that at visit 1. A secondary analysis of the primary outcome, on an intention-to-treat (ITT) basis, will be performed using a mixed model regression to take into account the longitudinal nature of the data, the form of which is specified below,

$$ISW\ Change_{ij} = \beta_0 + \beta_k(Covariates)_{ij} + \beta_{k+1}(Trial\ Arm)_i + \varepsilon_{ij},$$

where the intercepts and slopes are allowed to vary by participant (*i*) and by arm (*j*), and there are *k* covariates to be included in the model. The covariates to be included in the model are:

- Previous completion of a PR programme (Yes/No)
- Multiple deprivation index (most deprived quintile of index, yes/no)
- Frailty status, measured using the Short Physical Performance battery (<10 / ≥10)
- MRC dyspnoea scale

Continuous covariates will be centred when added to the model. The effect estimates and their 95% confidence intervals will be reported. Appendix table A8 details the blank table for these results.

The proportion of participants achieving the MCID for the primary outcome measure will be compared using chi squared tests, comparing the two arms in change from visit 1 to visit 2. The same will be done for visit 1 to visit 3. This is presented in table A14.

The effect size and the 95% confidence interval of the effect size will be represented graphically in both arms in a forest plot in order to determine non-inferiority. This is presented in figure A5.

The handling of missing data is described in 4.1.3. The robustness of the findings will be assessed and further uncertainties will be addressed by sensitivity analysis as described in 4.1.5.

4.1.2 Analysis of secondary outcomes

The secondary outcomes (CRQ-D, CRQ-F, CRQ-E, CRQ-M and CRQ-Total and QMVC) will be analysed in a similar fashion to primary outcome. Appendix tables A9 and A10 shows how outcome data will be displayed in the report.

For the non-inferiority analysis, delta (or the non-inferiority margin) will be defined as 1.25 points in relation to the CRQ-D and 5 points in relation to the CRQ-T. A MCID for QMVC has not been established, but based on previously published data, the delta will be set at 1.25kg. The test used will be independent sample t-tests, where the 95% CI of the between group difference will be represented graphically in a forest plot in order to determine non-inferiority. This is presented in figure A6.

The secondary outcomes are all change variables, similar to the primary outcomes, as the change from baseline to visit 2. As a secondary analysis of the secondary outcomes, on an intention to treat basis, a mixed model regression will be performed to take into account the longitudinal nature of the data, the general form of which model is specified below,

$$Secondary\ Outcome\ Change_{ij} = \beta_0 + \beta_k(Covariates)_{ij} + \beta_{k+1}(Trial\ Arm)_i + \varepsilon_{ij},$$

Where the intercepts and slopes are allowed to vary by participant (*j*) and by arm (*i*), and there are *k* covariates to be included in the model. The covariates included in the model are the same as those to be included in the model for the primary outcome analysis. These are:

- Previous completion of a PR programme (Yes/No)
- Multiple deprivation index (most deprived quintile of index, yes/no)
- Frailty status, measured using the Short Physical Performance battery (<10 / ≥10)
- MRC dyspnoea scale

Continuous covariates will be centred when added to the model. The effect estimates and their 95% confidence intervals will be reported.

The proportion of participants achieving the MCID for the secondary outcome measures will be compared using chi squared tests, comparing the two arms in change from visit 1 to visit 2. The same will be done for visit 1 to visit 3. This is presented in table A15.

PR completion rates and the number of PR sessions attended will be reported in both groups and compared using a chi squared test and independent sample t test, respectively. This is presented in table A13.

4.1.3 Statistical considerations

4.1.3.1 Missing data

For baseline variables, the number of participants with complete data will be reported.

The primary and secondary outcomes will be analysed as per the recommendations of White et al.[6] Missing post-randomisation assessments will be dealt with using an appropriate method of imputation, dependent on an appropriate missing mechanism, such as missing at random.

4.1.3.2 *Handling multiple comparisons*

No correction will be made for primary outcome comparison..

4.1.3.3 *Handling non-adherence*

We will record and classify the following variables:

- Adherence: The number of supervised pulmonary rehabilitation sessions the participant attends irrespective of the assigned intervention (maximum 16). This will be recorded in both intervention arms. Adherers will be recorded as a dichotomous indicator with 8 sessions attended as the threshold to be classified as "adherer".

The following variables will be recorded in a 3 x 2 table (blank table in appendix, table A11) according to allocated intervention:

- Compliers always receive the allocated treatment.
- Complete defiers do the opposite of the allocated treatment i.e. attend no sessions in allocated treatment.
- Partial defiers are all others not falling in the above groups

	Adherers	Non-adherers
Compliers	≥8 sessions, all in allocated treatment	<8 sessions, all allocated treatment
Complete defiers	≥8 sessions, 0 in allocated treatment	<8 sessions, 0 in allocated treatment
Partial defiers	Not falling in above groups	

Dropout cause will be recorded using the MORECARE classification of reason for attrition.[4]

In addition to the primary intention-to-treat analysis, the effect of actually receiving treatment as defined in the protocol will also be estimated. If non-compliance is >10% a Complier-Average Causal-Effect (CACE) will be estimated.

4.1.4 *Analysis of adverse events and survival*

The difference of adverse events between groups will be compared using Chi square test or Fisher's exact test for count, and Mann-Whitney U test for length of hospital stay; the survival curve will be compared using a statistical test that is sensitive to early difference (e.g., Generalised Wilcoxon test).

The survival curve from recruitment to death/last follow up by trial arm will be plotted using the Kaplan-Meier method.

4.1.4.1 Model assumption checks

The t-test makes multiple assumptions about the data. The assumptions are that the observations are independent, there are no significant outliers, data is normally distributed, and the homogeneity of variances. These assumptions can be tested using box plots for outliers, q-qnorm plots to assess the normality, and the quality of variances using Levene's test. If these assumptions do not hold, then an alternative method will be used in place of the t-test.

For the mixed model, the assumptions made are that the explanatory variables are linearly related to the response variables, the errors have constant variance, the errors are independent and the errors are normally distributed. To test this, the explanatory variables will be plotted against the response variable, the residuals will be plotted against the fitted values from the model, and the residuals will be plotted on a q-qnorm plot to assess for normality of errors. If the assumption of explanatory variables being linearly related to response variables does not hold, the explanatory variables can be transformed for this assumption to hold.

The chi-squared test makes multiple assumptions. The data must be frequencies, and variables are categorical, and that those categories are mutually exclusive. The groups must also be independent, and the count in at least 80% of the distinct categories should be more than 5 and have an expected value of above 1. The data shall be tabulated to check that more than 80% of the cells have a frequency of over 5 and satisfy that assumption.

The Kaplan-Meier curve that will be plotted for survival of the participants of the study also makes some assumptions. The plot assumes that the survival probability is the same for censored and uncensored subjects, the likelihood for the occurrence of the event is the same for the participants enrolled early and late, the probability of censoring is the same for different groups, and the event is assumed to have occurred at the defined time.

4.1.5 Sensitivity analyses

The following sensitivity analyses are planned:

- Per protocol
- Including only those in the upper quartile for baseline ISW test distance score
- Including only those with a primary diagnosis of COPD
- Including only those who have a complete case
- Complier Average Causal Effects (CACE) analysis[8]
- Generalised Estimating Equation analysis to estimate treatment effects, adjusting for smoking status (3 levels, current, ex or never smoker), participant age and gender.

Blank tables to summarise these sensitivity analyses are table A8 and table A9 in the appendix.

4.2 Interim and subgroup analyses

In view of the short duration of the trial and the sample heterogeneity, we do not plan any interim analysis or subgroup analysis.

4.3 Exploratory analyses

Any other analyses, not specifically identified in the protocol and in this document, will be considered exploratory in nature and will be clearly identified in the report.

5 Software

An online data collection system for clinical trials (MACRO; InferMed Ltd) will be used. This is hosted on a dedicated server at King's College London and managed by the KCTU. The KCTU Data Manager will extract data as needed and provide these in comma separated (.csv) format. STATA 17 (or equivalent) will be used for all analyses.

6 Amendment

Owing to the COVID-19 pandemic the study was suspended for 13 months between February 2020 and 2021, and, at the date of study suspension, there were 60 recruited and randomised participants waiting to start pulmonary rehabilitation but were unable to receive the interventions or complete the 8-week measurement due to the pandemic. Accordingly, a substantial amendment to increase the sample size from 362 to 436 participants and extend the recruitment and follow-up periods by one year to 31st March 2022 and 2023 respectively was submitted to the funders (National Institute for Health Research), the Health Research Authority and research ethics committee on 5th February 2021. This amendment was approved on 24th February 2021.

The statistical team changed to Mr Callum Glen as the trial statistician and Dr Francesca Fiorentino as the senior statistician.

The statistical analysis plan was updated to version 2.0. to reflect protocol amendments.

7 References

1. Piaggio G, Elbourne DR, Pocock SJ, et al. Reporting of noninferiority and equivalence randomized trials: extension of the CONSORT 2010 statement. *Jama* 2012;308(24):2594-604.

2. Wangge G, Putzeist M, Knol MJ, et al. Regulatory scientific advice on non-inferiority drug trials. *PLoS One* 2013;8(9):e74818.

3. Singh SJ, Jones P, Evans R, et al. Minimum clinically important improvement for the incremental shuttle walking test. *Thorax* 2008;63(9):775-77.

4. Higginson IJ, Evans CJ, Grande G, et al. Evaluating complex interventions in end of life care: the MORECare statement on good practice generated by a synthesis of transparent expert consultations and systematic reviews. *BMC medicine* 2013;11(1):111.

5. Kolahi J, Bang H, Park J. Towards a proposal for assessment of blinding success in clinical trials: up‐to‐date review. *Community dentistry and oral epidemiology* 2009;37(6):477-84.

6. White IR, Horton NJ, Carpenter J, et al. Strategy for intention to treat analysis in randomised trials with missing outcome data. *Bmj* 2011;342:d40.

7. Benjamini Y, Hochberg Y. Controlling the false discovery rate: a practical and powerful approach to multiple testing. *Journal of the Royal statistical society: series B (Methodological)* 1995;57(1):289-300.

8. Jo B. Statistical power in randomized intervention studies with noncompliance. *Psychological Methods* 2002;7(2):178.

8 Appendices

8.1 MISTER - Forms to expect completed depending on trial status

Summary timeline of forms to be completed at each visit

MISTER	Visit 1 (IA)	Randomi sation	Visit 2 (EoC)	Visit 3 (1 year)	Ongoing
1. Registration Form	x				
2. Eligibility	x				
3. Multiple Deprivation Index	x				
4. Randomisation Form		x			
5. Trial Status Form			x	x	
6. Primary Respiratory Diagnosis	x				
7. Other Medical History	x				
8. Charlson Comorbidity Index	x				
9. Structured History	x			x	
10. Medical Research Council Dyspnoea Score	x		x	x	
11. Spirometry	x			x	
12. Anthropometry	x		x	x	
13. Chronic Respiratory Questionnaire	x		x	x	
14. EQ-5D-5L	x		x	x	
15. Incremental Shuttle Walk Test	x		x	x	
16. Short Physical Performance Battery	x		x	x	
17. Quadriceps Maximum Voluntary Contraction	x		x	x	
18. Modified CSRI (Part 1)	x		x	x	
19. Modified CSRI (Part 2)	x		x	x	
20. Rehab Completion			X (still completed if missed visit)		
21. Global Rating of Change Questionnaire			x		
22. Global Rating of Satisfaction			x		
23. Adverse Events Log					x
24. Discontinuation of Intervention					x
25. Withdrawal from Trial Form					x
**Total:**					

8.1.1 *Visit 1*

All forms for this visit will be completed. No forms should be left blank. Any missing data should be coded appropriately.

8.1.2 *Visit 2*

TRIAL STATUS FORM OPTION	FORMS LEFT BLANK	FORMS STILL COMPLETED
1 – Participating in trial	**None**	**All forms** for this visit
2 – Missed Visit	• Medical Research Council Dyspnoea Score<br>• Anthropometry<br>• Chronic Respiratory Questionnaire<br>• EQ-5D-5L<br>• Incremental Shuttle Walk Test<br>• Short Physical Performance Battery<br>• Quadriceps Maximum Voluntary Contraction<br>• Modified CSRI (part 1)<br>• Modified CSRI (part 2)<br>• Global Rating of Change Questionnaire<br>• Global Rating of Satisfaction	• Trial Status Form<br>• Rehab Completion
3 – Withdrawn from further data collection	• Medical Research Council Dyspnoea Score<br>• Anthropometry	• Trial Status Form<br>• Rehab Completion

	• Chronic Respiratory Questionnaire<br>• EQ-5D-5L<br>• Incremental Shuttle Walk Test<br>• Short Physical Performance Battery<br>• Quadriceps Maximum Voluntary Contraction<br>• Modified CSRI (part 1)<br>• Modified CSRI (part 2)<br>• Global Rating of Change Questionnaire<br>• Global Rating of Satisfaction	
4 - Deceased	• Medical Research Council Dyspnoea Score<br>• Anthropometry<br>• Chronic Respiratory Questionnaire<br>• EQ-5D-5L<br>• Incremental Shuttle Walk Test<br>• Short Physical Performance Battery<br>• Quadriceps Maximum Voluntary Contraction<br>• Modified CSRI (part 1)<br>• Modified CSRI (part 2)<br>• Global Rating of Change Questionnaire<br>• Global Rating of Satisfaction	• Trial Status Form<br>• Rehab Completion

Please note: The adverse events log, discontinuation of intervention form and withdrawal from trial form are all ongoing and not associated with a specific visit. These will be completed for each patient by the end of the trial and any missing data will be coded appropriately.

8.1.3 Visit 3

TRIAL STATUS FORM OPTION	FORMS LEFT BLANK	FORMS THAT ARE STILL COMPLETED
1 – Participating in trial	**None**	**All forms** for this visit
2 – Missed Visit	• Structured History<br>• Medical Research Council Dyspnoea Score<br>• Spirometry<br>• Anthropometry<br>• Chronic Respiratory Questionnaire<br>• EQ-5D-5L<br>• Incremental Shuttle Walk Test<br>• Short Physical Performance Battery<br>• Quadriceps Maximum Voluntary Contraction<br>• Modified CSRI (part 1)<br>• Modified CSRI (part 2)	• Trial Status Form
Withdrawn from further data collection	• Structured History<br>• Medical Research Council Dyspnoea Score<br>• Spirometry<br>• Anthropometry<br>• Chronic Respiratory Questionnaire<br>• EQ-5D-5L<br>• Incremental Shuttle Walk Test<br>• Short Physical Performance Battery<br>• Quadriceps Maximum Voluntary Contraction<br>• Modified CSRI (part 1)<br>• Modified CSRI (part 2)	• Trial Status Form

4 - Deceased	• Structured History<br>• Medical Research Council Dyspnoea Score<br>• Spirometry<br>• Anthropometry<br>• Chronic Respiratory Questionnaire<br>• EQ-5D-5L<br>• Incremental Shuttle Walk Test<br>• Short Physical Performance Battery<br>• Quadriceps Maximum Voluntary Contraction<br>• Modified CSRI (part 1)<br>• Modified CSRI (part 2)	• Trial Status Form
Please note: The adverse events log, discontinuation of intervention form and withdrawal from trial form are all ongoing and not associated with a specific visit. These will be completed for each patient by the end of the trial and any missing data will be coded appropriately.		

8.2 MISTER - Tables to be included in the statistical report

8.2.1 *Table A1 - Summary table of baseline characteristics by treatment arm*

Table A1 – Summary of the baseline characteristics by treatment group and overall. The summary is presented as mean 95% confidence interval unless otherwise stated, and units are stated where necessary.

Baseline characteristic	PR-min	PR-specialist	Overall
Age *(mean, sd)*			
Age *(median, IQR)*			
Gender *(n, %)*			
Male			
Female			
Ethnicity *(n, % of overall)*			
White			
Chinese			
Irish Traveller			
Indian			
Pakistani			
Bangladeshi			

Black African			
Black Other			
Mixed Ethnic Group			
Other Ethnic Group			

8.2.2 *Table A2 – Summary of baseline outcome measures*

Table A2 – Table of outcome measurements at baseline. The summary is presented as means and standard deviations unless otherwise stated, units are stated where necessary.

Baseline measurement<br><br>*(Mean, SD)*	PR-min	PR-specialist	Overall
Short Physical Performance Battery			
ISW (metres)			
CRQ-D			
CRQ-F			
CRQ-E			
CRQ-M			
CRQ-T			
QMVC			
FEV_1 (litres)			
FVC (litres)			

8.2.3 *Table A3 – Summary of baseline characteristics by the validity of ISW score at visit 2*

Table A3 – Summary of the baseline characteristics by the validity of ISW distance score at visit 2.

Baseline characteristic	Valid ISW Score at visit 2, PR-min	Valid ISW Score at visit 2, PR-specialist
Age *(mean, SD)*		
Age *(median, IQR)*		
Gender *(n, %)*		
Male		
Female		
Ethnicity *(n, % of overall)*		
White		
Chinese		
Irish Traveller		
Indian		
Pakistani		
Bangladeshi		
Black African		

Black Other		
Mixed Ethnic Group		
Other Ethnic Group		

8.2.4 Table A4 – Summary of baseline outcome measures by validity of ISW test score

Table A4 – Table of outcome measurements at baseline by validity of ISW test score at visit 2. The summary is presented as means and standard deviations, units are stated where necessary.

Baseline Measurement<br><br>*(Mean, SD)*	Valid ISW Score at visit 2, PR-min	Valid ISW Score at visit 2, PR-specialist
Short Physical Performance Battery		
ISW (metres)		
CRQ-D		
CRQ-F		
CRQ-E		
CRQ-M		
CRQ-T		
QMVC		
FEV_1 (litres)		
FVC (litres)		

8.2.5 Table A5 - Summary table of primary outcome statistics

Table A5 – Summary of the primary outcome measurements by treatment group and overall in metres.

Primary outcome	PR-min	PR-specialist	Overall
ISW, mean (SD)			
Visit 1			
Visit 2			
Visit 3			

8.2.6 Table A6 – Summary table of secondary outcome statistics

Table A6 – Summary of the secondary outcome measurements by treatment group and overall in metres.

Secondary outcome	PR-min	PR-max	Overall
CRQ-D			
Visit 1			
Visit 2			
Visit 3			
CRQ-F			
Visit 1			
Visit 2			
Visit 3			
CRQ-E			
Visit 1			
Visit 2			
Visit 3			

CRQ-M			
Visit 1			
Visit 2			
Visit 3			
CRQ-T			
Visit 1			
Visit 2			
Visit 3			
QMVC			
Visit 1			
Visit 2			
Visit 3			
Adherence, (n, %)			
Visit 1			

8.2.7 Table A7 - Primary outcome analysis

Table A7 – Results of the model for the primary outcome of ISW, with a one-sided significance of 0.025, using intention to treat analysis.

Parameter	Coefficient	95% CI	P value
Constant			
PR-min			
K Adjusting variables			

8.2.8 Table A8 – Primary outcome analysis – secondary analysis

Table A8 – Results of the mixed model secondary analysis for the primary outcome of ISW, with a one-sided significance of 0.025, using intention to treat analysis.

Parameter	Coefficient	95% CI	P value
Constant			
PR-min			
K Adjusting variables			

8.2.9 Table A9 – Secondary outcome analysis (pre- to post-PR)

Table A9 – Results of the models between pre- (visit 1) and post-PR(visit 2) for CRQ-D (model 1), CRQ-T (model 2) and QMVC (model 3), with a one sided significance of 0.025.

Parameter	Coefficient	95% CI	P value
Model 1			
Constant			
PR-min			
Adjusting variables			
Model 2			
Constant			
PR-min			
Adjusting variables			
Model 3			
Constant			
PR-min			
Adjusting variables			

8.2.10 Table A10 – Secondary outcome analysis (pre-PR to 12 months)

Table A10 – Results of the models between pre-PR and 12-month follow-up for CRQ-D (model 1), CRQ-T (model 2) and QMVC (model 3), with a one sided significance of 0.025.

Parameter	Coefficient	95% CI	P value
Model 1			
Constant			
PR-min			
Adjusting variables			
Model 2			
Constant			
PR-min			
Adjusting variables			
Model 3			
Constant			
PR-min			
Adjusting variables			

8.2.11 Table A11 – Compliance and Adherence table

Table A11 – Table classifying all participants as to their compliance and to their adherence throughout the study.

	Adherers	Non-adherers
Compliers		
Complete defiers		
Partial defiers		

8.2.12 Table A12 – Sensitivity analyses for primary outcome data

Table A12 – Results of the two-sample t test for the primary outcome of ISW, with a one-sided significance of 0.025, using alternative populations as sensitivity analyses for the primary analyses.

These tables will be the same as seen in table A7, section 8.2.7, one of each type for each of the sensitivity analyses proposed. These analyses are:

- Per protocol analysis
- Including only those who are in the upper quartile for baseline ISW test distance score
- Including only those who have a primary diagnosis of COPD
- A complete case analysis
- A complier average causal effects (CACE) analysis
- A generalised estimating equation analysis to estimate treatment effects, adjusting for smoking status, participant age and gender

8.2.13 Table A13 – PR completion rate and attendance

Table A13 – The percentage of participants in each arm who completed PR alongside the mean number of PR sessions attended in each arm. The tests for difference between arms are also shown.

	PR-Min	PR-Specialist	Test Statistic	P value
Completion rate *(n, %), chi squared test*				
Sessions attended *(Mean, SD), independent T test*				

8.2.14 Table A14 – Minimal clinically important difference – Primary Outcome

Table A14 – Percentage of participants in each arm that achieved the MCID for the primary outcome, ISW **at each visit (1, 2 and 3)**

Timeframe	PR-Min	PR-Specialist	P value *(chi squared test)*
Visit 1 – 2			
Visit 1 – 3			

8.2.15 Table A15 – Minimal Clinically Important Difference – Secondary outcomes

Table A15 – Percentage of participants in each arm that achieved the MCID for the secondary outcomes between visits 1 and 2, and between visits 1 and 3.

QMVC

Timeframe	PR-Min	PR-Specialist	P value *(chi squared test)*
Visit 1 – 2			
Visit 1 – 3			

CRQ-T

Timeframe	PR-Min	PR-Specialist	P value *(chi squared test)*
Visit 1 – 2			
Visit 1 – 3			

CRQ-D

Timeframe	PR-Min	PR-Specialist	P value *(chi squared test)*
Visit 1 – 2			
Visit 1 – 3			

8.3 MISTER - Tables to be included in the statistical report

8.3.1 Figure A1

Figure A1 – Graph of the recruitment of participants over time. Periods where the UK was in a lockdown are marked in [colour].

8.3.2 Figure A2

Figure A2 – Graph of mean scores of the primary outcome, ISW, over time in each arm.

8.3.3 Figure A3

Figure A3 – Graph of mean scores of secondary outcomes over time in each arm. (a) CRQ-D, (b) CRQ-T and (c) QMVC.

8.3.4 Figure A4

Figure A4 – Box plots showing the primary outcome (ISW) at (a) visit 1 and at (b) visit 2.

8.3.5 Figure A5

Figure A5 – Forest plot of primary outcome, showing the effect size and it's 95% confidence interval in each arm with the non-inferiority margin.

8.3.6 Figure A6

Figure A6 – Forest plot of secondary outcome measures showing the effect sizes and their 95% confidence intervals in each arm, alongside the non-inferiority margin for each measure. (a) QMVC. (b) CRQ-D, (c) CRQ-T.

8.3.7 Figure A7

Figure A7 – Kaplan Meier survival curve, where participants are censored at either death or last follow-up point, whichever event occurs first.

Signatures

Dr William Man

[signature]

Date 20/04/2023

Dr Matthew Maddocks

Matthew Maddocks Digitally signed by Matthew Maddocks
Date: 2023.04.17 15:42:20 +01'00'

Date

Dr Claire Nolan

Claire nolan Digitally signed by Claire nolan
Date: 2023.04.13 10:08:00 +01'00'

Date

Dr Francesca Fiorentino

Date

Mr Callum Glen

Callum Glen (k2149490) Digitally signed by Callum Glen (k2149490)
Date: 2023.04.13 09:34:01 +01'00'

Date
