# Supplementary material for: Minimal vs Specialized Exercise Equipment for Pulmonary Rehabilitation: A Randomized Clinical Trial
Source: JAMA Netw Open. 2025 Aug 12;8(8):e2526616. doi: 10.1001/jamanetworkopen.2025.26616 (PMC12344533; doi:10.1001/jamanetworkopen.2025.26616)
Supplement: Supplement 3. — eAppendix 1. Supplementary methods eTable 1. Study outcome measures eAppendix 2. Supplementary results eTable 2. Primary outcome (ISW): Linear mixed model adjusting for age, dyspnea and the stratification factors (previous PR completion, multiple deprivation index, frailty) eTable 3. Generalised estimating equation-based analysis for incremental shuttle walk test between visits 1 and 2 eFigure 1. Non-inferiority graph at visit 2 for Chronic Respiratory Questionnaire a) Total, b) Dyspnea domain, c) Fatigue domain, d) Emotion domain, e) Mastery domain, f) QMVC eFigure 2. Non-inferiority graph at visit 3 for Chronic Respiratory Questionnaire a) Total, b) Dyspnea domain, c) Fatigue domain, d) Emotion domain, e) Mastery domain, f) QMVC eFigure 3. Graph of data over time by trial arm for a) incremental shuttle walk test, b) Chronic Respiratory Questionnaire-Total, c) Chronic Respiratory Questionnaire-Total-Dyspnea, d) QMVC eAppendix 3. Economic Analysis eTable 4. Mean costs at each visit, (2022 GBP), prior to multiple imputation eTable 5. Mean HRQoL at each visit, prior to multiple imputation eTable 6. Bootstrapped treatment effect estimates, PR delivered using minimal equipment versus PR gym eTable 7. Compliance with allocated trial arm eTable 8. Reasons participants did not comply with allocated trial arm eTable 9. Baseline characteristics of participants who switched or did not switch from their allocated treatment arm eTable 10. Number of adverse events and serious adverse events related to the intervention, by study arm eFigure 4. Kaplan-Meier curve demonstrating time to all-cause mortality during the trial according to trial arm eTable 11. Number of deaths by study arm and body system code eReferences [file jamanetwopen-e2526616-s003.pdf]

Supplemental Online Content

Nolan CM, Glen C, Walsh JA, et al. Minimal vs specialized exercise equipment for pulmonary rehabilitation: a randomized clinical trial. *JAMA Netw Open*. 2025;8(8):e2526616. doi:10.1001/jamanetworkopen.2025.26616

eAppendix 1. Supplementary methods
eTable 1. Study outcome measures
eAppendix 2. Supplementary results
eTable 2. Primary outcome (ISW): Linear mixed model adjusting for age, dyspnea and the stratification factors (previous PR completion, multiple deprivation index, frailty)
eTable 3. Generalised estimating equation-based analysis for incremental shuttle walk test between visits 1 and 2
eFigure 1. Non-inferiority graph at visit 2 for Chronic Respiratory Questionnaire a) Total, b) Dyspnea domain, c) Fatigue domain, d) Emotion domain, e) Mastery domain, f) QMVC
eFigure 2. Non-inferiority graph at visit 3 for Chronic Respiratory Questionnaire a) Total, b) Dyspnea domain, c) Fatigue domain, d) Emotion domain, e) Mastery domain, f) QMVC
eFigure 3. Graph of data over time by trial arm for a) incremental shuttle walk test, b) Chronic Respiratory Questionnaire-Total, c) Chronic Respiratory Questionnaire-Total-Dyspnea, d) QMVC
eAppendix 3. Economic Analysis
eTable 4. Mean costs at each visit, (2022 GBP), prior to multiple imputation
eTable 5. Mean HRQoL at each visit, prior to multiple imputation
eTable 6. Bootstrapped treatment effect estimates, PR delivered using minimal equipment versus PR gym
eTable 7. Compliance with allocated trial arm
eTable 8. Reasons participants did not comply with allocated trial arm
eTable 9. Baseline characteristics of participants who switched or did not switch from their allocated treatment arm
eTable 10. Number of adverse events and serious adverse events related to the intervention, by study arm
eFigure 4. Kaplan-Meier curve demonstrating time to all-cause mortality during the trial according to trial arm
eTable 11. Number of deaths by study arm and body system code
eReferences

This supplemental material has been provided by the authors to give readers additional information about their work.

eAppendix 1. Supplementary Methods

Randomization and masking

Participants were randomised at the individual level with a 1:1 allocation ratio, using an independent web-based system provided by the UK Clinical Research Collaboration-registered King's Clinical Trials Unit (KCTU), to receive either intervention (PR-min) or control (PR-gym). Randomization by minimisation was used to balance the following characteristics: previous completion of PR (yes/no), multiple deprivation index (most deprived quintile of index: yes/no[1] and frailty status (Short Physical Performance Battery score: SPPB $<10/\geq10$).[2] The randomization system settings were established with a 90% likelihood of minimization and a 10% likelihood of simple randomization. Once randomised, the system automatically generated a full audit trail of the process and sent an email to relevant investigators in a blinded or unblinded format, depending on their role. For each arm, participants were provided with a choice of three sites in northwest London to undertake PR. Owing to the nature of the interventions, participants and providers of the intervention were not blinded. However, all post-PR assessments (visit 2, visit 3) were performed by a researcher blinded to group allocation and not involved in the delivery of either intervention arm. The trial statistician was also blinded to group allocation.

Trial oversight

The trial was overseen by a Data Monitoring and Ethics Committee which included an independent chair and statistician, and met annually. The committee reviewed adverse and serious adverse event data on 21/06/2019 and identified an imbalance in baseline characteristics, namely a higher number of participants with a primary respiratory diagnosis of COPD and exacerbations requiring medication change in the past year, in one of the trial arms. They thought this may be due to small group numbers (PR-min n=55, PR-gym n=56) and/or the imbalance in primary respiratory diagnosis. They reviewed the same data on 21/01/2020 and as there was an equal number of safety events in each arm, the committee recommended that the trial should continue.

Patient and public involvement (PPI)

People affected by chronic lung disease were involved throughout as members of the study team. The concept of the research project arose directly from PPI, with one PPI representative (NS, named co-author) involved in the study design and named as a co-applicant on the funding application. NS was involved in the management of the research through membership of the Trial Steering Group that met throughout the study. NS representative was joined by a second representative (JM) who met the trial co-ordinator for regular PPI meetings. In addition, the PPI representatives provided input into written material for participants, advice on participant recruitment, protocol amendments, supporting analysis interpretation, and contributing to this publication (NS). Following publication of the research, they will have a role in dissemination of research findings, particularly to lay audiences.

Study interventions

PR-gym (control group)

The control intervention was current gold-standard clinical practice.[3-5] PR-gym comprised an in-person 8-week outpatient exercise and multidisciplinary self-management education program, with two supervised and at least one additional home session each week, and delivered according to international guidelines for PR.[3-5] Each supervised session consisted of 1 hour of exercise (at least 30 minutes aerobic exercise) and 45 minutes of education. Supervising staff comprised specialist respiratory therapists with a minimum of two years' experience in PR.

Available specialist aerobic exercise equipment included treadmills, cycle ergometers and cross-trainers. machines. Initial walking speed prescription on the treadmill was 80% of predicted peak oxygen consumption based on baseline

© 2025 Nolan CM et al. *JAMA Network Open.*

ISW performance[6] whilst endurance cycling was initially set to achieve level 3 to 4 on the Borg CR-10 Dyspnea Scale[7] with the aim of patients completing 10 minutes of continuous training.

Available specialist lower limb resistance exercise equipment included leg press and knee extension weights. Lower limb resistance training comprised two to four sets of eight to 12 repetitions at an initial training load of 60% one repetition maximum, as per the American College of Sports Medicine guidelines, on a leg press and bilateral knee extension specialist weights machine with participants aiming to achieve a Borg Rating of Perceived Exertion score of 13–15 (on a scale of 6–20).[7] This was supplemented with sit-to-stand sets, plus knee lifts/extension and hip abduction with appropriate ankle weights up to 10 kg, as well as upper limb exercises including biceps curls, shoulder press and upright row with free weights or elastic resistance bands (red to black). For these resistance exercises, two to four sets of eight to 12 repetitions were prescribed and participants aimed to achieve a Borg Rating of Perceived Exertion score of 13–15 (on a scale of 6–20).[7]

Exercise training was individualised and regularly progressed according to standard operating procedures with targets reviewed at each session. Aerobic exercise was progressed by increasing exercise duration (aiming for 30 minutes exercise) or intensity e.g. treadmill: increase in speed and incline, cycle ergometer and cross-trainer: increase in watts. Resistance exercise was progressed by increasing the number of sets, repetitions and weight.

Education was delivered by a multidisciplinary team with topics chosen to develop participants' understanding and holistic management of their disease. Further details on the education program and safety measures for the rehabilitation program are described in subsequent sections of this file.

PR-min (intervention group)

PR-min also comprised an in-person 8-week outpatient exercise and multidisciplinary self-management education program, with two supervised and at least one additional home session each week, and delivered according to international guidelines for PR.[3-5] Similar to PR-gym, each supervised session consisted of 1 hour of exercise (at least 30 minutes aerobic exercise) and 45 minutes of education, and supervising staff comprised specialist respiratory therapists with at least two years' experience of in PR. The same staff delivered both PR-gym and PR-min.

There was no access to treadmills, cycle ergometers or specialist resistance equipment. Available aerobic exercise equipment included a walking circuit, portable steppers, portable pedals. Initial walking speed prescription was 80% of predicted peak oxygen consumption based on baseline ISW performance. Participants were provided with a stopwatch and given time targets to complete a walking course of known distance. Although the resistance of the portable pedals can be manually adjusted, this cannot be objectively quantified. Initial prescription was set at 'level 1' but individually adjusted to find an intensity where patients could complete 10 minutes of continuous training with a target modified Borg CR10-Dyspnea score of 3–4[7]

Available resistance exercise equipment included hand and ankle weights (up to 5 kg) and elastic resistance bands (red to black). Exercises included functional activities such as sit-to-stand and step-ups as well as elastic resistance band-based exercises such as sitting knee extension, leg press and hip flexion as well as standing hip extension, squats, chest press and lateral raise. Resistance training was prescribed as described in the PR-gym section, that is two to four sets of eight to 12 repetitions aiming to achieve a Borg Rating of Perceived Exertion score of 13–15 (on a scale of 6–20).[7]

Exercise training was individualised and regularly progressed according to standard operating procedures with targets reviewed at each session. Aerobic exercise was progressed by increasing exercise duration (aiming for 30 minutes exercise) or intensity e.g. walking course: increase in speed i.e number of circuits to complete in a specific time, portable pedals: increase in resistance. Resistance exercise was progressed by increasing the number of sets, repetitions and where relevant weight or elastic band resistance (from red to black).

Education was delivered by a multidisciplinary team as per PR-gym, and further details on this and the safety measures for the rehabilitation program are described in subsequent sections of this file.

© 2025 Nolan CM et al. *JAMA Network Open.*

Education component of PR

A multidisciplinary team, including physiotherapists, psychologists, dieticians, nurses, doctors, occupational therapists, social workers, speech and language therapists and expert patients, delivered the education sessions. The aim of the education program was to develop patients' understanding and holistic management of their disease, and topics included pacing strategies, breathing control exercises, physical activity and exercise, medication use, diet, smoking cessation, management of anxiety and low mood, managing infections through early recognition, rescue medication, appropriate general practice/hospital presentation and loving relationships. The sessions took place twice per week for 45 minutes and patients received a booklet of the topics covered in these sessions.

Safety measures for PR-min and PR-gym programs

- The program was delivered in a suitable time and in easily accessible buildings and locations with adequate parking, good transport links and suitable for people with disabilities;
- A minimum of two members of staff were required to supervise group exercise classes and education sessions, one of whom must be a registered allied health professional;
- There was a minimum of two members of staff supervising group exercise classes and education sessions in community settings. There was an absolute minimum of one staff to eight people for supervised group exercise. However, the ratio could be increased to one staff to four people depending on the complexity and severity of the participants' medical problems, and this was judged by the responsible member of the senior clinical staff;
- There were no more than two ambulatory or long-term oxygen users in any one exercise class;
- Prior to each session a risk and suitability assessment of the PR venue was undertaken;
- Unwell patients were managed using an emergency care protocol. In short, if urgent help was required in a community setting, an ambulance was called, and in a hospital setting, the crash team were called;
- Contraindications to exercise were screened at the PR assessment;
- Patients stopped exercising if $Sp0_2$ <80% and if they felt unwell e.g. chest pain, nausea or if a member of staff deemed this was necessary;
- The following monitoring equipment was required: pulse oximeters, blood pressure monitor;
- The following emergency equipment was required:
    - Community-based programs: Portable defibrillator and bag-valve mask;
    - Hospital-based programs: crash trolley
    - Both programs: Portable oxygen; tubing; face-mask; glucose kit; bio-hazard spill kit; first aid kit

Study outcome measures

A list of the scheduled outcomes is outlined in table E1.

eTable 1. Study Outcome Measures

	**Visit 1**	**Visit 2**	**Visit 3**
**Primary outcome measure**	X	X	X
Exercise capacity (Incremental Shuttle Walk Test)			
**Secondary outcome measures**			
Dyspnea (Chronic Respiratory Questionnaire – Dyspnea domain)	X	X	X
Health-related quality of life (Chronic Respiratory Questionnaire)	X	X	X

© 2025 Nolan CM et al. *JAMA Network Open.*

Isometric quadriceps strength (Isometric Quadriceps Maximum Voluntary Contraction)	X	X	X
**Miscellaneous outcomes measures e.g. to characterise patients at baseline, minimization criteria, health-economic data*, safety**			
Spirometry	X		
Frailty (Short Physical Performance Battery)	X	X	X
Charlson Comorbidity Index	X		
EQ5D-5 Levels*	X	X	X
Modified Client Service Receipt Inventory*	X	X	X
Participant self-reported change in condition (Global Rating of Change Questionnaire)		X	
Participant PR program satisfaction (Global Rating of Change Questionnaire)		X	
Safety and trial process evaluation	X	X	X

*Health economic data will be presented in a separate paper

Primary outcome measure

Exercise capacity: Visit 1, visit 2 and visit 3

The incremental shuttle walk test (ISW) is an incremental, externally paced, field walking test that involves participants walking around a 10-metre course in time to a series of progressively faster beeps played from a CD player. At visit 1**,** two tests (a practice test and then the formal test) were undertaken with at least 30 minutes rest between tests.[6] This is in accordance with international technical standards.[8] Measures of breathlessness, oxygen saturation levels and heart rate were recorded before and after the test. The distance completed in metres was also be recorded.

Secondary outcome measures

Dyspnea: Visit 1, visit 2 and visit 3

Dyspnea was measured using the Chronic Respiratory Questionnaire-Dyspnea domain (CRQ-D).[9] The dyspnea domain allows the participants to choose five activities that were limited by shortness of breath in the previous two weeks. Each item is scored on a seven-point Likert scale, with a lower score indicating a greater dyspnea.

Health-related quality of life: Visit 1, visit 2 and visit 3

Health-related quality of life was measured using the CRQ.[9] This 20-item questionnaire, is responsive to PR, and contains four domains; dyspnea (described above), fatigue, emotional function and mastery, and a total score. Each item is scored on a seven-point Likert scale, with a lower score indicating a higher symptom burden.

© 2025 Nolan CM et al. *JAMA Network Open.*

Isometric quadriceps strength: Visit 1, visit 2 and visit 3

Isometric quadriceps maximal voluntary contraction of the dominant leg was measured using specially designed chair and strain gauge. This test involves the participant pushing against an ankle strap with the knee positioned at 90^0.[10] A warm-up and six efforts were performed.

Miscellaneous outcome measures

Spirometry: Visit 1

All participants underwent spirometry performing the Forced Vital Capacity (FVC) / Forced Expiratory Volume in one second (FEV_1) manoeuvre, using an EasyOne™ diagnostic spirometer. As a minimum, three technically acceptable tests must be performed and must meet the Association of Respiratory Technology and Physiology reproducibility criteria [11] Results obtained included FVC, FEV_1 and the FEV_1/FVC ratio. Percent predicted reference equations from the European Respiratory Society/European Steel and Coal Society were used.[12]

Frailty: Visit 1, visit 2 and visit 3

All participants performed the Short Physical Performance Battery (SPPB) which is a simple test of lower limb functional performance and a marker of frailty.[2] It comprises an assessment of standing balance, usual walking speed and ability to stand from a chair. The SPPB is scored out of 12 with a higher score indicating better functional performance. A score of <10 indicates a frail status and this cut-off was used in the randomization procedure.

Charlson Comorbidity Index: Visit 1

The Charlson Comorbidity Index is a measure of disease burden. It consists of 17 comorbidities which are weighted from 1 to 6 for mortality risk and disease severity and summed to form the index score.[13]

EQ-5D-5 Levels: Visit 1, visit 2, visit 3

The EQ5D5L is a generic measure of health status that comprises a visual analogue scale and five-item questionnaire with the following domains: mobility; self-care; usual activities; pain/discomfort and anxiety/depression which is used to calculate a utility index.[14]

Modified Client Service Receipt Inventory: Visit 1, visit 2, visit 3

The Modified Client Service Receipt Inventory questionnaire will be used to record information on health care resource, medicine and equipment use, informal care provided by family members, time off work and costs borne by the patient and family relating to the chronic lung disease. Health economic data will be presented in a separate paper.

Participant self-reported change in condition: Visit 2

To measure self-reported change in condition, participants rated their response to the following questions on a five-point Global Rating of Change Questionnaire.[15]

- *"How do you feel your overall condition has changed after rehabilitation?"* The scale ranged from *"1: I feel much better"* to *"5: I feel much worse".*

Participant PR program satisfaction: Visit 2

© 2025 Nolan CM et al. *JAMA Network Open.*

To measure patient satisfaction, participants rated their response to the following questions on a five-point Global Rating of Change Questionnaire.[15]

- *"How satisfied are you with your rehabilitation program?"* The scale ranged from *"1: Very satisfied"* to *"5: Very dissatisfied".*

Safety and trial process evaluation: Visit 1, visit 2, visit 3

- Safety was assessed in real-time using adverse event reporting. A Serious Adverse Event (SAE) was defined as an untoward occurrence that a) resulted in death; b) was life-threatening; c) required hospitalisation or prolongation of existing hospitalisation; d) resulted in persistent or significant disability or incapacity or e) was otherwise considered medically significant by the investigator.
- PR uptake, adherence and completion were assessed objectively through PR attendance registers and training records. Reasons for non-completion of PR were assessed objectively through PR records.

Impact of the Covid-19 pandemic described in line with the CONSERVE statement[16]

Extenuating circumstance: COVID-19 resulted in the closure of the PR service and cessation of in-person research activity in line with Trust guidance.

Impact: Study recruitment and in-person assessment was suspended for 13 months between February 2020 and 2021. At the date of recruitment suspension, 74 recruited and randomised participants were waiting to start PR but were unable to receive either intervention due to the closure of the PR service or undergo in-person assessment due to the cessation of in-person research activity. This led to missing data. This was particularly relevant for the primary outcome, the incremental shuttle walk, which requires face to face assessment and supervision. This was a significant contributor to the disparity between the numbers providing incremental shuttle walk data and the numbers assessed at research visits (Figure 1).

When the study re-opened, the in-person assessments and PR interventions were conducted in line with infection control guidance, with no change in staff:participant ratio. Even when COVID-19 restrictions were formally lifted, some participants decided to remain shielded and declined in-person assessments but were happy to complete the questionnaire-based outcomes at home.

Mitigating strategies: The following amendments were submitted to the funders (National Institute for Health and Care Research), the Health Research Authority and research ethics committee for approval.

- A substantial amendment to increase the sample size from 362 to 436 and extend the study duration by one year to 31st March 2022 to ensure the study had sufficient power and enable completion of visit 2 and 3 assessments was approved in February 2021. The funder provided additional funds to undertake this work.
- A substantial amendment to permit participant consent via telephone-based assessments whilst in-person assessments were not permitted was approved in March 2021.
- A non-substantial amendment to extend the study duration by an additional year to 31st March 2023 to enable completion of visit 3 assessments was approved in May 2022. The funder did not provide additional funding to undertake this work.

Responsible parties: The non-substantial amendment was planned by the Trial Management Group, led by the Chief Investigator (WM). The substantial amendments were planned by the Trial Management Group, led by the Chief Investigator (WM). The Trial Steering Committee reviewed and approved the proposed plans to submit the substantial amendments. The funders (National Institute for Health and Care Research), the Health Research Authority and research ethics committee approved all amendments.

Interim data: The Trial Management and Steering Groups reviewed the number of recruited participants who did not receive PR because of the PR service closure and the number of participants who did not have primary outcome data collected at visit 2.

Statistical analysis

The statistical analysis plan has been published previously[17] and is available on the study's ISRCTN webpage: ISRCTN - ISRCTN16196765: Minimal versus specialist equipment in the delivery of pulmonary rehabilitation

For baseline data, continuous and categorical variables were summarized with descriptive statistics. No significance testing was undertaken. The outcome measures were described by trial arm and at visits 1, 2 and 3 using descriptive statistics.

The main statistical analyses estimated the difference in mean primary and secondary outcomes between participants randomised to PR-gym and PR-min by intention to treat principle from visit 1 to visit 2 (eight weeks following visit 1) and visit 1 to visit 3 (12 months following visit 1). Group difference estimates and associated one-sided 97.5% confidence intervals are reported. The group differences were compared using one-sided independent sample t-test or non-parametric equivalent and a linear mixed model adjusting for age, trial arm, baseline Medical Research Council (MRC) dyspnea score, previous PR completion, multiple deprivation index and SPPB score. The significance level was set at one-sided significance level of 0.025.

Regarding missing data, the number of baseline variables with complete data were reported and the primary and secondary outcomes were analysed as per the recommendations of White and colleagues,[18] to perform a main analysis on all observed data under a plausible missing assumption and then perform sensitivity analyses.

For compliance with the allocated trial arm, the following variables were recorded in a 3x2 table according to the allocated intervention: 1) compliers always receive the allocated intervention; 2) complete defiers do the opposite of the allocated intervention i.e. attend no sessions in allocated treatment; 3) partial defiers are all others not falling in the above groups. Dropout reason was recorded using the MORECARE classification of reason for attrition.[19] Intervention adherence was defined as the number of supervised sessions the participant attended irrespective of the assigned intervention (maximum 16). This was recorded in both intervention arms, as a dichotomous indicator with eight sessions as the cut off for adherence.

In addition to the primary intention-to-treat analysis, the effect of treatment receipt as defined in the protocol was also estimated (per-protocol analysis: participants complied with the protocol and did not switch trial arms at any point). If non-compliance rate with PR sessions was >10%, a Complier-Average Causal-Effect (CACE) was estimated (i.e. to provide causal intervention effect estimates).[19] The following sensitivity analyses were planned: 1) a generalised estimating equation-based analysis to estimate the treatment effects, adjusting for imbalance if the group difference in the percentage of smokers, participants age <70 years, or females is greater than 20% at baseline; 2) primary diagnosis of COPD; 3) participants in the upper quartile for baseline ISW and 4) complete case.

Similar to the primary outcome, the non-inferiority margin for secondary outcomes was defined as half the known minimal important difference (MID) using the fixed-margin method with a preserved effect of 50%.[20, 21] The MID and non-inferiority margin of the CRQ was 0.5 and -0.25 respectively.[22] The MID of QMVC has not been published, but local audit data demonstrated that the mean change in QMVC with PR was 2.5kg, therefore the non-inferiority margin was set at -1.25kg.

The statistical analysis plan has been published previously [17] and is available on the study's ISRCTN webpage: ISRCTN - ISRCTN16196765: Minimal versus specialist equipment in the delivery of pulmonary rehabilitation

© 2025 Nolan CM et al. *JAMA Network Open.*

eAppendix 2. Supplementary Results

eTable 2. Primary outcome (ISW): Linear mixed model adjusting for age, dyspnea and the stratification factors (previous PR completion, multiple deprivation index, frailty)

	Coefficient	Standard error	95% confidence interval	p-value
Constant*	-14.87	41.23	-95.68 to 65.93	
Age	-0.65	0.48	-1.59 to 0.30	0.182
Trial arm				
PR-gym	(Reference)			
PR-min	0.78	9.49	-17.83	0.005**
Difference between trial arms				
At visit 2	(Reference)			
At visit 3	3.07	16.07	-28.42	0.03**
MRC score				
1	(Reference)			
2	-0.92	28.39	-56.55 to 54.72	0.97
3	14.03	28.34	-41.51 to 69.56	0.62
4	4.94	28.61	-51.13 to 61.01	0.86
5	-6.24	32.12	-69.19 to 56.70	0.85
Previous PR completion				
Yes	(Reference)			
No	2.94	9.13	-14.97 to 20.84	0.75
Multiple Deprivation Index				
Lowest quintile	(Reference)			
Other quintile	34.80	30.22	-24.42 to 94.03	0.25
Short Physical Performance Battery				
≥10	(Reference)			
<10	-2.78	9.67	-21.74 to 16.18	0.78

* Taken as reference for this table, and therefore included in the constant at those in the control arm, of average age (70.03), with an MRC Dyspnea score of 1, who had completed PR previously, were in the lowest quintile of the Multiple Deprivation Index, and their SPPB score was more than or equal to 10, at 8 months.

** The p-value presented is for PR-Minimal and is from the one-sided t-test of the marginal estimate at that timepoint that the difference between treatments is not equal to or less than (one-sided) -24.0m, the non-inferiority margin. All other p-values are two-sided t-tests to 0.

Abbreviations: ISW: Incremental Shuttle Walk Test; MRC: Medical Research Council; PR: Pulmonary Rehabilitation; PR-Min: PR Delivered Using Minimal Equipment; SPPB: Short Physical Performance Battery.

© 2025 Nolan CM et al. *JAMA Network Open.*

eTable 3. Generalised estimating equation-based analysis for incremental shuttle walk test between visits 1 and 2

	Coefficient	Standard error	95% confidence interval	p-value
Constant	-14.69	38.72	-90.59 to 61.20	
Age	-0.75	0.46	-1.65 to 0.15	0.10
Trial arm				
PR-gym	(Reference)			
PR-min	1.76	9.48	-16.82	0.003*
Difference between trial arms and baseline at				
Visit 2	(Reference)			
Visit 3	3.19	18.01	-32.11	0.030*
MRC score				
1	(Reference)			
2	3.45	26.64	-48.75 to 55.66	0.90
3	20.87	26.68	-31.41 to 73.15	0.43
4	9.34	26.90	-43.38 to 62.05	0.73
5	-2.05	30.30	-61.43 to 57.33	0.95
Previous PR completion				
Yes	(Reference)			
No	3.97	8.53	-12.75 to 20.69	0.64
Multiple Deprivation Index				
Lowest quintile	(Reference)			
Other quintile	29.73	28.24	-25.62 to 85.07	0.29
SPPB				
≥10	(Reference)			
<10	-5.21	9.04	-22.94 to 12.51	0.56

Note: the group difference in the percentage of smokers, participants age <70 years, or females was less than 20% at baseline.

* The p-value presented is for PR-Minimal and is from the one-sided t-test of the marginal estimate at that timepoint that the difference between treatments is not equal to or less than (one-sided) -24m, the non-inferiority margin. All other p-values are t-tests to 0.

Abbreviations: ISW: Incremental Shuttle Walk Test; MRC: Medical Research Council; PR: Pulmonary Rehabilitation; PR-Min: PR Delivered Using Minimal Equipment; SPPB: Short Physical Performance Battery.

© 2025 Nolan CM et al. *JAMA Network Open.*

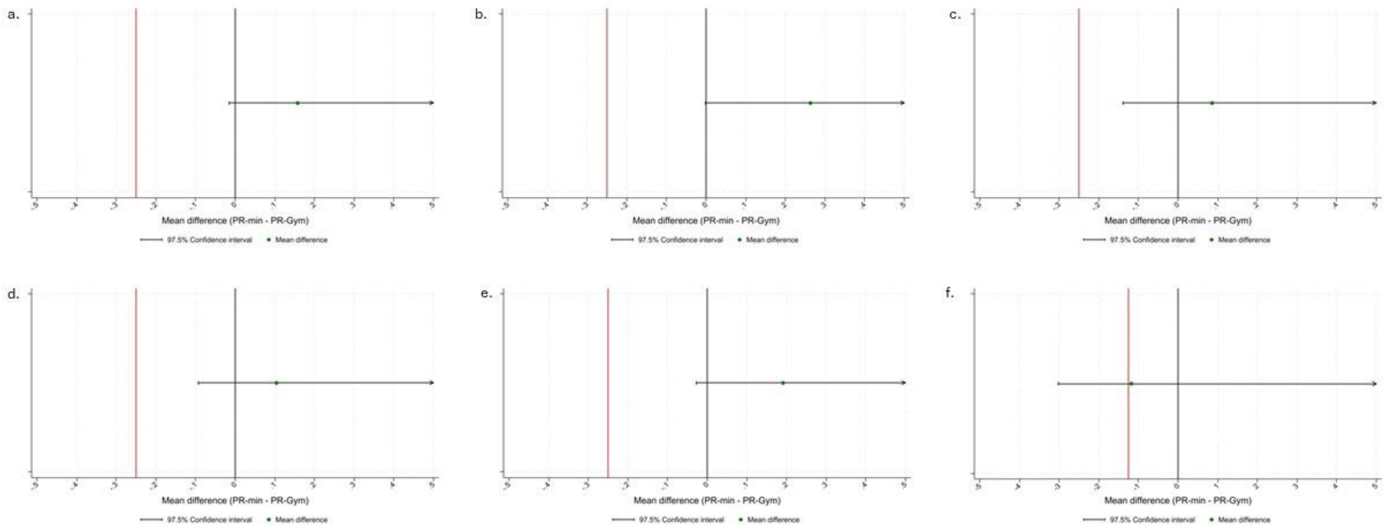

eFigure 1. Non-inferiority graph at visit 2 for Chronic Respiratory Questionnaire a) Total, b) Dyspnea domain, c) Fatigue domain, d) Emotion domain, e) Mastery domain, f) QMVC.

Abbreviations: Pulmonary Rehabilitation; PR-Min: PR Delivered Using Minimal Equipment; QMVC: Isometric Quadriceps Maximum Voluntary Contraction.

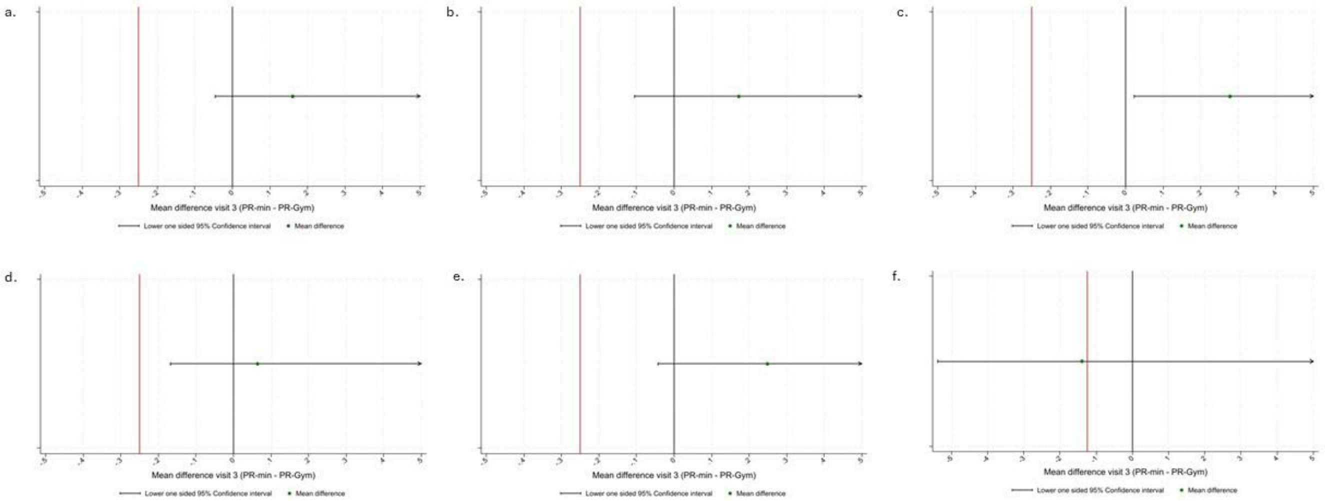

eFigure 2. Non-inferiority graph at visit 3 for Chronic Respiratory Questionnaire a) Total, b) Dyspnea domain, c) Fatigue domain, d) Emotion domain, e) Mastery domain, f) QMVC.

Abbreviations: Pulmonary Rehabilitation; PR-Min: PR Delivered Using Minimal Equipment; QMVC: Isometric Quadriceps Maximum Voluntary Contraction.

© 2025 Nolan CM et al. *JAMA Network Open.*

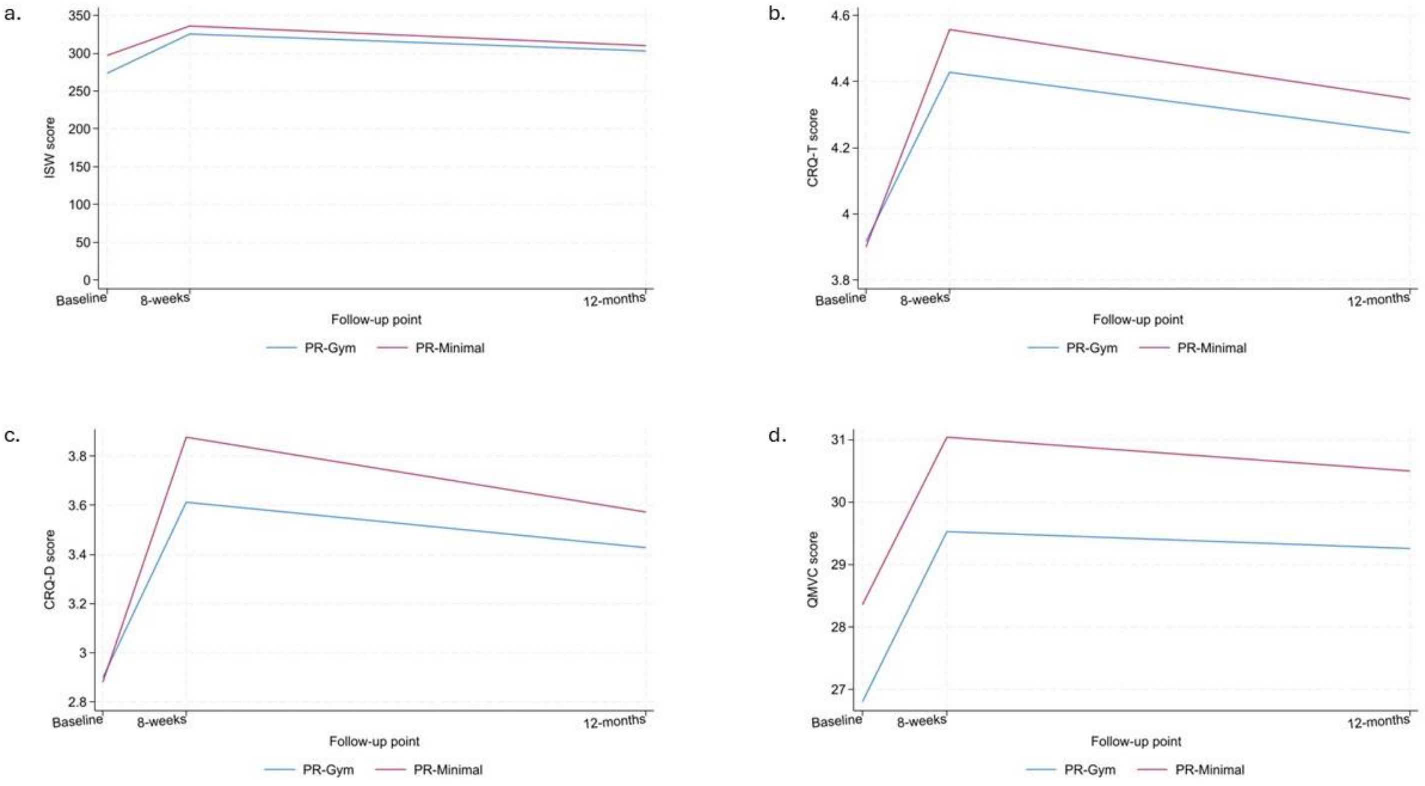

eFigure 3. Graph of data over time by trial arm for a) incremental shuttle walk test, b) Chronic Respiratory Questionnaire-Total, c) Chronic Respiratory Questionnaire-Total-Dyspnea, d) QMVC

Abbreviations: CRQ: Chronic Respiratory Questionnaire; CRQ-D: Dyspnea Domain; CRQ-T: CRQ-Total; ISW: Incremental Shuttle Walk Test; PR: Pulmonary Rehabilitation; QMVC: Isometric Quadriceps Maximum Voluntary Contraction.

© 2025 Nolan CM et al. *JAMA Network Open.*

eAppendix 3. Economic Analysis

Background, rationale and aim

MISTER was a trial to evaluate whether outpatient pulmonary rehabilitation delivered using minimal equipment is non-inferior to traditional pulmonary rehabilitation delivered using specialist exercise equipment. A health economic evaluation compares a treatment or intervention against one or more comparators in terms of their effects on costs and outcomes in order to inform stakeholders' decision-making.[23]

Our aim for the economic analysis was to evaluate and report the cost-effectiveness of outpatient pulmonary rehabilitation delivered using minimal equipment compared to traditional pulmonary rehabilitation using data collected as part of the MISTER trial. We conducted a standard economic evaluation of the two programs under consideration, consistent with the study protocol.[17]

Methods

Economic evaluation framework

Perspective, time horizon and discount rate

Both utilisation and EuroQoL EQ-5D-5L data were collected at baseline (visit 1), eight weeks from baseline (visit 2) and one year from baseline (visit 3). We report formal costs from the system perspective in all analyses. The primary endpoint was visit 2, at the end of the eight-week programme. We conducted sensitivity analysis from baseline to visit 3. In all analyses we utilise the patient perspective, in line with NICE guidance. No discount rate was used since the trial entry and outcomes were within a year.

Selection, measurement and valuation of outcomes

We combined EuroQoL EQ-5D-5L.[24] responses into quality-adjust life years (QALYs) using the recommended mapping model for UK value sets under extant guidance.[25, 26] We combined EQ-5D-5L responses from three timepoints, adjusting for time between data collection using linear interpolation.

Selection, measurement and valuation of resources and costs

We collected frequency of health care use using the modified Client Services Receipt Inventory (CSRI).[27] The modified CSRI covered relevant categories of care including hospital care (emergency department, inpatient admissions, outpatient appointments), community-based services (e.g., primary care physician, public health/community nursing, allied health), residential care and equipment. We estimated costs by combining reported frequency in the modified CSRI with unit costs for each service, using a combination of National Health Service tariffs and Personal Social Services Research Unit databases.[28, 29] The NHS tariff for each intervention is the same and this was therefore treated as ignorable.

In primary analysis, modified CSRI responses covered the entirety of the period (visit 1 to visit 2). In secondary analysis, we addressed the unobserved period for healthcare use between day 57 and day 275 using linear interpolation.

Statistical methods

Missing data due to attrition or mortality

For each of the three visits, if a participant answered any modified CSRI question but left others blank then we assumed this blank was zero. If the participant did not answer any modified CSRI questions then their healthcare use was deemed missing at random.

Missing data at visit 1 was very low (<1% of participants). We addressed missing baseline outcomes by predicting for all participants EQ-5D-5L score and log-transformed costs in cross-sectional ordinary least squares (OLS) regressions

© 2025 Nolan CM et al. *JAMA Network Open.*

where predictors were age, sex, Charlson comorbidity score and any EQ-5D-5L domains with non-missingness; for participants missing an outcome variable at baseline, we imputed the predicted value.

To account for missing outcome data at visits 2 and 3, both among participants who did not answer the modified CSRI and/or EQ-5D-5L questions and non-participants who were still alive, we estimated costs and HRQoL outcome variables using multiple imputation by chained equations (MICE; 50 imputations), assuming missing data were missing at random (MAR).[28] We used as independent predictors age, sex, Charlson comorbidity score, baseline EQ-5D-5L and baseline log-transformed costs.[30, 31] Where a participant's death was reported between two data collection points, we adjusted costs and QALYs assuming death occurred at the midpoint using linear interpolation.

Analytics and sampling uncertainty

We entered unit costs and adjusted to the year 2022 using the health component of the consumer price index in Microsoft Excel.[32] We performed all other data processing and analyses in Stata (version 17).[33] We estimated effects on costs and QALYs using seemingly unrelated regressions (SUR), with Stata code provided by Mutubuki et al.[34] In all regressions predictors were the same as in MICE: age, sex, Charlson comorbidity score, baseline EQ-5D-5L and baseline log-transformed costs. In each analysis we used non-parametric bootstrapping with 1000 replications in each of the 50 MICE-generated datasets and then combined the 50,000 bootstrapped estimates.[35] We estimated incremental cost-effectiveness ratios (ICER) and the probability that the intervention was cost-effective for willingness to pay thresholds from £20,000 to £40,000 per QALY.[36]

Results

Study parameters

Mean healthcare costs prior to multiple imputation are presented by study arm in table E4. There was no missingness at baseline (n=436). Of those enrolled, 355 (81%) answered the modified CSRI at Visit 1 and 334 (77%) at Visit 2. Mean costs for the three months prior to baseline were £2849 (standard deviation (SD): 9084), for the first two months of the study were £1588 (SD: 6911) and for the last three months of the study were £2362 (SD: 12976). Different arms had higher mean costs at different timepoints.

eTable 4. Mean costs at each visit, (2022 GBP), prior to multiple imputation

	PR-min		PR-gym		All	
	**n**	**Mean (SD)**	**n**	**Mean (SD)**	**N**	**Mean (SD)**
**Visit 0 (baseline)**	218	1981 (5219)	218	3717 (11691)	436	2849 (9084)
**Visit 1 (56 days)**	179	1541 (7350)	176	1635 (6455)	355	1588 (6911)
**Visit 2 (365 days)**	168	2886 (16329)	166	1831 (8328)	334	2362 (12976)

Mean HRQoL score (where 1 = full health, 0 = dead and negative scores are possible), prior to multiple imputation, are presented by study arm in table E4. Sample retention was very similar to the modified CSRI response rate. Mean HRQoL score at baseline was 67% full health, rising to 70% at Visit 1 but regressing to 65% at Visit 2. Scores were very similar across arms.

© 2025 Nolan CM et al. *JAMA Network Open.*

eTable 5. Mean HRQoL at each visit, prior to multiple imputation

	PR-min		PR-gym		All	
	**n**	**Mean (SD)**	**n**	**Mean (SD)**	**N**	**Mean (SD)**
**Visit 0 (baseline)**	218	0.67 (0.24)	218	0.66 (0.26)	436	0.67 (0.25)
**Visit 1 (56 days)**	181	0.70 (0.24)	172	0.70 (0.23)	353	0.70 (0.23)
**Visit 2 (365 days)**	169	0.66 (0.25)	163	0.65 (0.25)	332	0.65 (0.25)

Summary of main results

Incremental treatment effects are presented in table E6. Minimal equipment was associated with lower costs and better HRQoL at Visit 2, but both estimates had a high level of uncertainty associated. At Visit 3, participants in the minimal equipment arm had higher costs and better QALYs, again with a high level of uncertainty. No result was close to statistical significance.

© 2025 Nolan CM et al. *JAMA Network Open.*

eTable 6. Bootstrapped treatment effect estimates, PR delivered using minimal equipment versus PR gym

	Incremental cost (95% CI)	Incremental QALYs (95% CI)	ICER (95% CI)	$p_{£20,000}$	$p_{£30,000}$	$p_{£40,000}$
Primary analysis						
To day 56	-218 (-1640 to 1205)	0.002 (-0.005 to 0.009)	-108790 (n.d.)	65%	65%	66%
Sensitivity analysis						
To day 365	1399 (-5913 to 8710)	0.003 (-0.040 to 0.046)	507280 (n.d.)	37%	37%	38%

Incremental estimates derived from 50,000 regression outputs (1000 bootstrap replications x 50 MICE imputations). **CI**=confidence interval. **ICER**=incremental cost-effectiveness ratio. **$p_{£y}$**= probability cost-effective if willing to pay £*y* per QALY. **SUR**=seemingly unrelated regressions. **n/a**: incremental QALYs estimated in primary analysis. **n/d**: 95% CI is not defined.

Conclusion

The economic evaluation found no evidence that one intervention was comparatively more cost-effective.

© 2025 Nolan CM et al. *JAMA Network Open.*

Compliance and adherence

Mean (standard deviation) distance from participants' homes to allocated PR site was 4.5 (0.3) miles for PR-min and 5.3 (0.4) miles for PR-gym. A higher proportion of participants complied with allocation to PR-gym (n=146, 67.0%) than PR-min (n=96, 44.0%). The most common reason for non-compliance with PR-gym was preference to attend PR at a specific location (n=24, 67.7%) and for PR-min, preference to do the non-allocated intervention (n=29, 39.2%).

A total of 242 participants complied with the allocated treatment arm: PR-min n=96 (44.0%); PR-gym n=146 (67.0%). Regarding non-compliance with the allocated treatment arm, 111 participants were classified as completed defiers (PR-min n=74 (66.7%); PR-gym n=37 (33.3%)) with preference to attend PR at a specific location as the most commonly reported reason (whole cohort: n=48; PR-min n=24 (50.0%); PR-gym n=24 (50.0%)). There were 83 partial defiers (PR-min n=48 (57.8%); PR-gym n=35 (42.2%)) with 'other reason' the most commonly cited explanation (whole cohort: n=25; PR-min n=16 (64.0%); PR-gym n=9 (36.0%)). Additional information is provided in tables E3-E4.

eTable 7. Compliance with allocated trial arm

	PR-min (n=218)	PR-gym (n=218)
Compliers	96 (44.0%)	146 (67.0%)
Complete defiers	74 (33.9%)	37 (17.0%)
Partial defiers	48 (22.0%)	35 (16.1%)

Data reported as number (percentage).

Note: Compliers always receive the allocated treatment; complete defiers do the opposite of allocated treatment; partial defiers are all others not falling into the other groups.

eTable 8. Reasons participants did not comply with allocated trial arm

**Complete defiers**	**Overall (n=111)**	**PR-min (n=74)**	**PR-gym (n=37)**
Preference to attend PR at a specific location	48 (43.2%)	24 (32.4%)	24 (64.9%)
Preference to do the non-allocated intervention	31 (27.9%)	29 (39.2%)	2 (5.4%)
Schedule of allocated intervention did not suit participant	16 (14.4%)	14 (18.9%)	2 (5.4%)
Difficulty travelling to allocated PR site	9 (8.1%)	1 (1.4%)	8 (21.6%)
Reason not recorded	4 (3.6%)	3 (4.1%)	1 (2.7%)
Other	3 (2.7%)	3 (4.1%)	0 (0.0%)
**Partial defiers**	**Overall (n=83)**	**PR-Min (n=48)**	**PR-Gym (n=35)**
Other	25 (30.1%)	16 (33.3%)	9 (25.7%)
Reason not recorded / unable to contact participant	18 (21.7%)	12 (25.0%)	6 (17.1%)
Preference to attend PR at a specific location	11 (13.3%)	5 (10.4%)	6 (17.1%)
Unwell (non-lung condition, not admitted to hospital)	8 (9.6%)	5 (10.4%)	3 (8.6%)
Declined PR	4 (4.8%)	2 (4.2%)	2 (5.7%)
Preference to do the non-allocated intervention	4 (4.8%)	3 (6.3%)	1 (2.9%)

Exacerbation of underlying lung condition (not admitted to hospital)	3 (3.6%)	2 (4.2%)	1 (2.9%)
Difficulty travelling to allocated PR site	2 (2.4%)	0 (0.0%)	2 (5.7%)
Admitted to hospital (underlying lung condition)	2 (2.4%)	1 (2.1%)	1 (2.9%)
Admitted to hospital (non-lung condition)	2 (2.4%)	0 (0.0%)	2 (5.7%)
Family commitments	2 (2.4%)	0 (0.0%)	2 (5.7%)
Schedule of allocated intervention did not suit	1 (1.2%)	1 (2.1%)	0 0 (0.0%)
Withdrew before intervention started	1 (1.2%)	1 (2.1%)	0 0 (0.0%)

Data reported as number (percentage).

Note: Complete defiers do the opposite of allocated treatment; partial defiers are all others not falling into the other groups.

eTable 9. Baseline characteristics of participants who switched or did not switch from their allocated treatment arm

	Baseline demographics, by arm and treatment switch status				
	**PR-Min (non-switcher)**	**PR-Min (switcher)**	**PR-Gym (non-switcher)**	**PR-Gym (switcher)**	**Total**
n (%)	144 (33.0%)	74 (17.0%)	181 (41.5%)	37 (8.5%)	436 (100.0%)
**Minimisation criteria**					
Previous PR completion	50 (34.7%)	39 (52.7%)	73 (40.3%)	15 (40.5%)	177 (40.6%)
Multiple Deprivation Index decile*	6.6 (2.5)	6.8 (2.2)	6.6 (2.5)	5.7 (2.1)	6.5 (2.4)
Frail (SPPB < 10)	80 (55.6%)	57 (77.0%)	116 (64.1%)	21 (56.8%)	274 (62.8%)
Sex					
Male	87 (60.4%)	39 (52.7%)	98 (54.1%)	15 (40.5%)	239 (54.8%)
Female	57 (39.6%)	35 (47.3%)	83 (45.9%)	22 (59.5%)	197 (45.2%)
Gender					
Male	87 (60.4%)	39 (52.7%)	98 (54.1%)	15 (40.5%)	239 (54.8%)
Female	57 (39.6%)	35 (47.3%)	83 (45.9%)	22 (59.5%)	197 (45.2%)
Age (years)	70.7 (10.1)	67.5 (12.3)	70.2 (10.7)	71.9 (9.5)	70.0 (10.7)
Weight (kg)	81.8 (71.2)	76.8 (17.5)	77.4 (21.2)	75.257 (17.63)	78.6 (44.0)
BMI (kg/m^2)	27.3 (7.3)	28.5 (6.3)	28.4 (7.0)	28.3 (6.2)	28.0 (6.9)
Smoking status					
Current	25 (17.4%)	9 (12.2%)	20 (11.0%)	3 (8.1%)	57 (13.1%)
Previous	91 (63.2%)	47 (63.5%)	120 (66.3%)	28 (75.7%)	286 (65.6%)
Never	28 (19.4%)	18 (24.3%)	41 (22.7%)	6 (16.2%)	93 (21.3%)
Pack year history	33.0 (33.6)	25.2 (27.0)	30.8 (31.3)	29.8 (24.2)	30.5 (30.9)

Primary respiratory diagnosis					
COPD	96 (66.7%)	41 (55.4%)	108 (59.7%)	28 (75.7%)	273 (62.6%)
Asthma	18 (12.5%)	15 (20.3%)	25 (13.8%)	3 (8.1%)	61 (14.0%)
Bronchiectasis	9 (6.2%)	5 (6.8%)	18 (9.9%)	4 (10.8%)	36 (8.3%)
ILD	21 (14.6%)	13 (17.6%)	30 (16.6%)	2 (5.4%)	66 (15.1%)
Spirometry					
FEV_1 (L)	1.40 (0.61)	1.55 (0.67)	1.48 (0.65)	1.35 (0.53)	1.47 (0.64)
FEV_1 (% predicted)	59.4 (21.9)	61.9 (25.8)	61.2 (25.8)	62.4 (20.1)	60.9 (24.2)
FVC (L)	2.53 (0.87)	2.65 (0.94)	2.53 (0.82)	2.40 (0.71)	2.54 (0.85)
FVC (% predicted)	83.6 (22.5)	86.6 (26.0)	83.0 (26.62)	88.2 (21.5)	84.4 (24.9)
Long term oxygen therapy	6 (4.2%)	5 (6.8%)	8 (4.4%)	1 (2.7%)	20 (4.6%)
Ambulatory oxygen therapy	9 (6.2%)	6 (8.1%)	17 (9.4%)	6 (16.2%)	38 (8.7%)
Exacerbations requiring medication change in the past year	1.4 (0.5)	1.4 (0.5)	1.4 (0.5)	1.4 (0.5)	1.4 (0.5)
Number of previous respiratory hospitalisations in the past 12 months	28 (19.4%)	20 (27.0%)	50 (27.6%)	16 (43.2%)	114 (26.1%)
Charlson Comorbidity Index score†	1.0 (1.0, 2.0)	1.0 (0.0, 1.0)	1.0 (1.0, 2.0)	1.0 (1.0, 2.0)	1.0 (1.0, 2.0)
MRC Dyspnea Scale†	3.2 (1.0)	3.0 (0.9)	3.1 (1.0)	3.2 (1.0)	3.2 (1.0)
1	2 (1.4%)	2 (2.7%)	5 (2.8%)	0 (0.0%)	9 (2.1%)
2	38 (26.4%)	21 (28.4%)	49 (27.1%)	9 (24.3%)	117 (26.8%)

3	41 (28.5%)	28 (37.8%)	66 (36.5%)	16 (43.2%)	151 (34.6%)
4	49 (34.0%)	21 (28.4%)	40 (22.1%)	9 (24.3%)	119 (27.3%)
5	14 (9.7%)	2 (2.7%)	21 (11.6%)	3 (8.1%)	40 (9.2%)
SPPB	9.4 (2.5)	10.7 (1.8)	9.9 (2.5)	8.3 (3.8)	9.7 (2.6)
ISW distance (m)	270.9 (171.5)	349.1 (213.5)	278.2 (168.6)	253.5 (187.8)	285.7 (181.3)
CRQ-Total*	3.8 (1.0)	4.1 (1.0)	3.9 (1.1)	3.9 (1.1)	3.9 (1.0)
CRQ-Dyspnea*	2.8 (1.1)	3.0 (1.0)	2.9 (1.1)	2.7 (1.0)	2.9 (1.1)
CRQ-Fatigue*	3.4 (1.2)	3.6 (1.3)	3.5 (1.3)	3.3 (1.3)	3.4 (1.3)
CRQ-Emotion*	4.4 (1.3)	4.7 (1.3)	4.4 (1.3)	4.5 (1.3)	4.5 (1.3)
CRQ-Mastery*	4.5 (1.4)	4.7 (1.3)	4.6 (1.4)	4.8 (1.5)	4.6 (1.4)
QMVC (kg)*	27.2 (10.0)	30.2 (13.9)	26.8 (9.3)	26.5 (12.6)	27.6 (10.8)
EQ5D-5 Levels Utility Index*	0.2 (0.2)	0.3 (0.2)	0.2 (0.2)	0.3 (0.3)	0.3 (0.2)
EQ5D-5Levels Visual Analogue Scale*	63.4 (20.4)	64.3 (18.8)	60.9 (19.0)	63.1 (20.9)	62.5 (19.6)

Data reported as mean (standard deviation), median (25th, 75th centile) or number (percentage).

*Scale interpretation: Higher score is better

†Scale interpretation: Higher score is worse

Abbreviations: BMI: Body Mass Index; COPD: Chronic Obstructive Pulmonary Disease; CRQ: Chronic Respiratory Questionnaire; FEV_1: Forced Expiratory Volume in One Second; FVC: Forced Vital Capacity; ILD: Interstitial Lung Disease; ISW: Incremental Shuttle Walk Test; MRC: Medical Research Council; PR: Pulmonary Rehabilitation; PR-Min: PR Delivered Using Minimal Equipment; QMVC: Isometric Quadriceps Maximum Voluntary Contraction; SPPB: Short Physical Performance Battery.

eTable 10. Number of adverse events and serious adverse events related to the intervention, by study arm

**Adverse event**	**PR-min (number)**	**PR-gym (number)**
**Musculoskeletal**	2	6
Other cardiovascular	0	1
Gastrointestinal	0	1
**Serious adverse events**	**PR-min (number)**	**PR-gym (number)**
Other respiratory	0	1

Data reported as number.

Abbreviations: PR: Pulmonary Rehabilitation; PR-Min: PR Delivered Using Minimal Equipment.

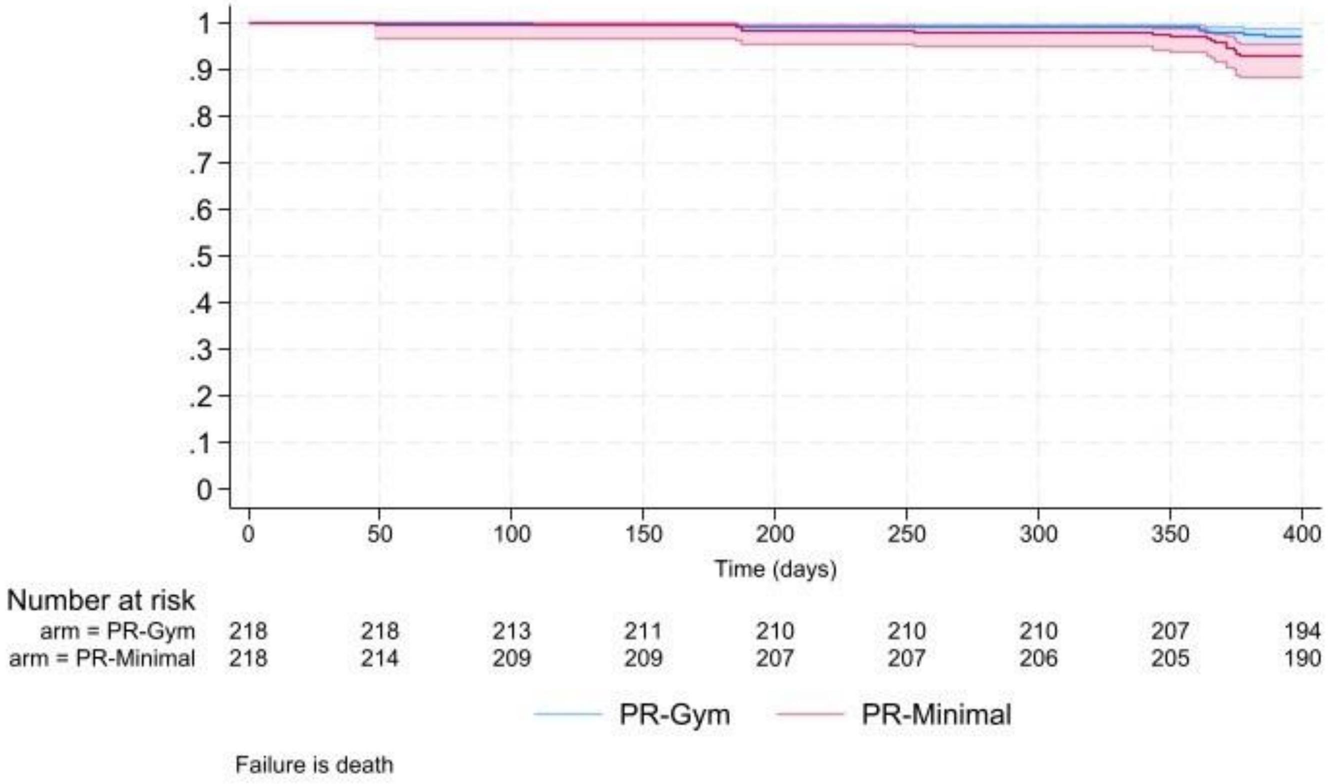

eFigure 4. Kaplan-Meier curve demonstrating time to all-cause mortality during the trial according to trial arm

Abbreviations: KM: Kaplan-Meier; PR: Pulmonary Rehabilitation; PR-Minimal: PR Delivered Using Minimal Equipment.

eTable 11. Number of deaths by study arm and body system code

Body system code	PR-min (number)	PR-gym (number)
Other respiratory	12	3
Falls requiring medical / nursing attention	1	0
Neurological	0	1
Other	2	2

Data reported as number.

Abbreviations: PR: Pulmonary Rehabilitation; PR-Min: PR Delivered Using Minimal Equipment.

eReferences

1. Ministry of Housing, Communities and Local Government. National statistics: English indices of deprivation 2015. https://www.gov.uk/government/statistics/english-indices-of-deprivation-2015. Accessed 4th July, 2023.

2. European Medicines Agency. Reflection paper on physical frailty: instruments for baseline characterisation of older populations in clinical trials. https://www.ema.europa.eu/en/documents/scientific-guideline/reflection-paper-physical-frailty-instruments-baseline-characterisation-older-populations-clinical_en.pdf. Accessed 5th July, 2023.

3. Alison JA, McKeough ZJ, Johnston K, et al. Australian and New Zealand Pulmonary Rehabilitation Guidelines. *Respirology*. 2017;22(4):800–819.

4. Rochester CL, Alison JA, Carlin B, et al. Pulmonary rehabilitation for adults with chronic respiratory disease: an official American Thoracic Society clinical practice guideline. *American Journal of Respiratory and Critical Care Medicine*. 2023;208(4):e7–e26.

5. Man W, Chaplin E, Daynes E, et al. British Thoracic society clinical statement on pulmonary rehabilitation. *Thorax*. 2023;78(Suppl 5):s2–s15.

6. Singh SJ, Morgan MD, Scott S, Walters D, Hardman AE. Development of a shuttle walking test of disability in patients with chronic airways obstruction. *Thorax*. 1992;47(12):1019–1024.

7. Borg G. *Borg's perceived exertion and pain scales.* Human kinetics; 1998.

8. Holland AE, Spruit MA, Troosters T, et al. An official European Respiratory Society/American Thoracic Society technical standard: field walking tests in chronic respiratory disease. *European Respiratory Journal*. 2014;44(6):1428–1446.

9. Williams JE, Singh SJ, Sewell L, Guyatt GH, Morgan MD. Development of a self-reported Chronic Respiratory Questionnaire (CRQ-SR). *Thorax*. 2001;56(12):954–959.

10. Swallow EB, Reyes D, Hopkinson NS, et al. Quadriceps strength predicts mortality in patients with moderate to severe chronic obstructive pulmonary disease. *Thorax*. 2007;62(2):115–120.

11. Sylvester KP, Clayton N, Cliff I, et al. ARTP statement on pulmonary function testing 2020. *BMJ Open Respiratory Research*. 2020;7(1):e000575.

12. Quanjer PH, Tammeling GJ, Cotes JE, Pedersen OF, Peslin R, Yernault JC. Lung volumes and forced ventilatory flows. *European respiratory journal*. 1993;6(Suppl 16):5–40.

13. Charlson ME, Pompei P, Ales KL, MacKenzie CR. A new method of classifying prognostic comorbidity in longitudinal studies: development and validation. *J Chronic Dis*. 1987;40(5):373–383.

14. Nolan CM, Longworth L, Lord J, et al. The EQ-5D-5L health status questionnaire in COPD: validity, responsiveness and minimum important difference. *Thorax*. 2016;71(6):493–500.

15. Patel S, Palmer MD, Nolan CM, et al. Supervised pulmonary rehabilitation using minimal or specialist exercise equipment in COPD: a propensity-matched analysis. *Thorax*. 2021;76(3):264–271.

16. Orkin AM, Gill PJ, Ghersi D, et al. Guidelines for reporting trial protocols and completed trials modified due to the COVID-19 pandemic and other extenuating circumstances: the CONSERVE 2021 statement. *JAMA*. 2021;326(3):257–265.

17. Nolan CM, Walsh JA, Patel S, et al. Protocol: Minimal versus specialist equipment in the delivery of pulmonary rehabilitation: protocol for a non-inferiority randomised controlled trial. *BMJ Open*. 2021;11(10).

18. White IR, Horton NJ, Carpenter J, Pocock SJ. Strategy for intention to treat analysis in randomised trials with missing outcome data. *BMJ*. 2011;342.

19. Higginson IJ, Evans CJ, Grande G, et al. Evaluating complex interventions in end of life care: the MORECare statement on good practice generated by a synthesis of transparent expert consultations and systematic reviews. *BMC medicine*. 2013;11:1–11.

20. Wangge G, Putzeist M, Knol MJ, et al. Regulatory scientific advice on non-inferiority drug trials. *PLoS One*. 2013;8(9):e74818.

21. Piaggio G, Elbourne DR, Pocock SJ, Evans SJ, Altman DG, CONSORT Group ft. Reporting of noninferiority and equivalence randomized trials: extension of the CONSORT 2010 statement. *JAMA*. 2012;308(24):2594–2604.

22. Schünemann HJ, Puhan M, Goldstein R, Jaeschke R, Guyatt GH. Measurement properties and interpretability of the Chronic respiratory disease questionnaire (CRQ). *COPD: journal of chronic obstructive pulmonary disease*. 2005;2(1):81–89.

23. Husereau D, Drummond M, Augustovski F, et al. Consolidated Health Economic Evaluation Reporting Standards 2022 (CHEERS 2022) statement: updated reporting guidance for health economic evaluations. *MDM Policy & Practice*. 2022;7(1):23814683211061097.

24. Rai M, Goyal R. Pharmacoeconomics in healthcare. In: *Pharmaceutical medicine and translational clinical research.* Elsevier; 2018:465–472.

25. Hernández Alava M, Pudney S, Wailoo A. Estimating the relationship between EQ-5D-5L and EQ-5D-3L: results from a UK population study. *Pharmacoeconomics*. 2023;41(2):199–207.

26. Devlin NJ, Shah KK, Feng Y, Mulhern B, Van Hout B. Valuing health-related quality of life: An EQ-5 D-5 L value set for E ngland. *Health Econ*. 2018;27(1):7–22.

27. Higginson IJ, Bausewein C, Reilly CC, et al. An integrated palliative and respiratory care service for patients with advanced disease and refractory breathlessness: a randomised controlled trial. *The Lancet Respiratory Medicine*. 2014;2(12):979–987.

28. Personal Social Services Research Unit, University of Kent. Unit Costs of Health and Social Care programme. https://www.pssru.ac.uk/unitcostsreport/.

29. National Health Service (England). Data from: National Cost Collection for the NHS. 2024.

30. Higginson IJ, Evans CJ, Grande G, et al. Evaluating complex interventions in end of life care: the MORECare statement on good practice generated by a synthesis of transparent expert consultations and systematic reviews. *BMC medicine*. 2013;11:1–11.

31. Van Asselt AD, Van Mastrigt GA, Dirksen CD, Arntz A, Severens JL, Kessels AG. How to deal with cost differences at baseline*. Pharmacoeconomics*. 2009;27:519–528.

32. Microsoft Excel. https://office.microsoft.com/excel.

33. StataCorp LLC. Stata Statistical Software: Release 17.

34. Mutubuki EN, El Alili M, Bosmans JE, et al. The statistical approach in trial-based economic evaluations matters: get your statistics together!*. BMC health services research*. 2021;21(1):475.

35. Glick H, Doshi JA, Sonnad SS, Polsky D. *Economic evaluation in clinical trials. Second edition. ed. Handbooks in health economic evaluation series.* 2nd ed. Oxford University Press; 2015.

36. Petrou S, Gray A. Economic evaluation alongside randomised controlled trials: design, conduct, analysis, and reporting*. BMJ*. 2011;342.
