# Supplementary material for: Minimal vs Specialized Exercise Equipment for Pulmonary Rehabilitation: A Randomized Clinical Trial
Source: JAMA Netw Open. 2025 Aug 12;8(8):e2526616. doi: 10.1001/jamanetworkopen.2025.26616 (PMC12344533; doi:10.1001/jamanetworkopen.2025.26616)
Supplement: Supplement 4. — Data Sharing Statement [file jamanetwopen-e2526616-s004.pdf]

Data Sharing Statement

Nolan. Minimal vs Specialized Exercise Equipment for Pulmonary Rehabilitation. *JAMA Netw Open*. Published August 12, 2025. doi:10.1001/jamanetworkopen.2025.26616

Data
Additional Information: ISRCTN:16196765 https://www.isrctn.com/ISRCTN16196765?q=minimal%20versus%20specialist%20equipment&filters=&sort=&offset=1&totalResults=1&page=1&pageSize=10
Data available: No

Additional Information
Explanation for why data not available: Consent to share participant data was not obtained
